# Enabling nucleophilic reactivity in molecular calcium fluoride complexes

**DOI:** 10.1038/s41557-024-01524-x

**Published:** 2024-05-14

**Authors:** Job J. C. Struijs, Mathias A. Ellwanger, Agamemnon E. Crumpton, Véronique Gouverneur, Simon Aldridge

**Affiliations:** Chemistry Research Laboratory, Department of Chemistry, University of Oxford, Oxford, UK

**Keywords:** Synthetic chemistry methodology, Chemical bonding, Chemical bonding

## Abstract

Calcium fluoride is the ultimate source of all fluorochemicals. Current synthetic approaches rely on the use of HF, generated from naturally occurring fluorspar and sulfuric acid. Methods for constructing E–F bonds directly from CaF_2_ have long been frustrated by its high lattice energy, low solubility and impaired fluoride ion nucleophilicity. Little fundamental understanding of the reactivity of Ca–F moieties is available to guide methodology development; well-defined molecular species containing Ca–F bonds are extremely rare, and existing examples are strongly aggregated and evidence no nucleophilic fluoride delivery. Here, by contrast, we show that by targeting anionic systems of the type [L_*n*_(X)_2_CaF]^−^, monomeric calcium fluoride complexes containing single Ca–F bonds can be synthesized, including via routes involving fluoride abstraction from existing C–F bonds. Comparative structural and spectroscopic studies of mono- and dinuclear systems allow us to define structure–activity relationships for E–F bond formation from molecular calcium fluorides.

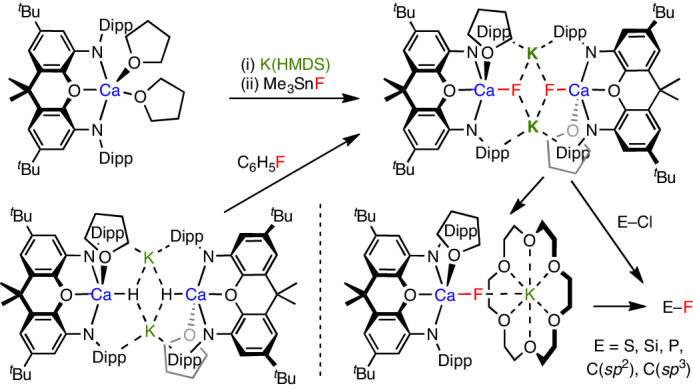

## Abstract (web summary)

Calcium difluoride is a source of fluorochemicals, but the reactivity of Ca–F moieties is not well understood. Now a library of molecular Ca–F complexes featuring unique structural motifs has been synthesized, including via fluorochemical defluorination. Studies of mono- and dinuclear systems provided structure–activity relationships for E–F bond formation.

## Main

Fluorine-containing molecules and materials play central roles in applications as diverse as lithium ion batteries, refrigerants, agrochemicals and pharmaceuticals^1,2^. Currently, all such compounds are ultimately derived commercially from the ore fluorspar (calcium difluoride) through the intermediacy or direct use of toxic and corrosive hydrogen fluoride (HF)^3–6^. The conversion of fluorspar into HF relies on the harsh and energetically intensive reaction with sulfuric acid, and hydrogen fluoride itself has been the cause of a number of high-profile fatal accidents^7^. The development of direct methods for the construction of E–F bonds from calcium fluoride derivatives, therefore, has clear scientific, environmental and commercial benefits. However, these attempts have long been frustrated by the strong tendency of such systems to aggregate, yielding materials with very low solubility and impaired fluoride ion nucleophilicity (Δ*H*_L_(CaF_2_) = +2,360 kJ mol^−^^1^)^8–13^. Very recently, we reported a method for the direct incorporation of fluoride from CaF_2_ into organic molecules through a solid-state process, which exploits the conversion of K_2_HPO_4_ into K_3_(HPO_4_)F under mechanochemical conditions^14^.

**Fig. 1:**
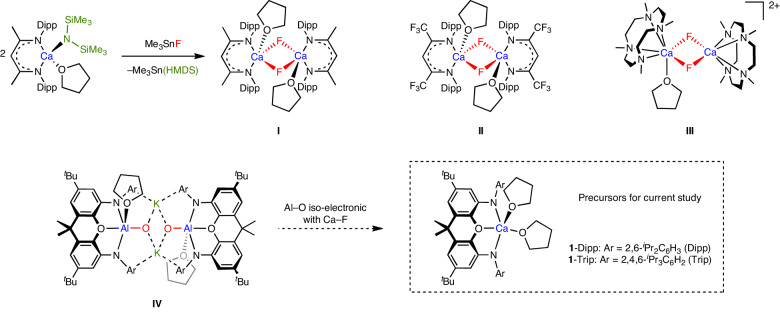
Relevant previous work and design rational behind current study. **I**–**IV**: dimeric calcium fluoride and related complexes. **1**-Dipp and **1**-Trip are calcium-containing precursors to fluoride complexes used in the current study, offering variation in ligand steric bulk.

Despite this advance, the scope for methodology development (particularly in the solution phase) is impaired by the lack of fundamental understanding of the reactivity of the Ca–F unit. Molecular species containing calcium fluoride fragments are very rare (approximately ten crystallographically characterized examples; for example, Fig. 1, **I**–**III**)^15–23^, and such moieties typically form part of polymetallic clusters (containing μ_2_-, μ_3_- and even μ_4_-fluoride) and rely on multidentate ligands at calcium to restrict further aggregation in solution and the solid state^18,19,23^. Furthermore, to our knowledge, there have been no reported examples of the nucleophilic delivery of F^−^ from such species. In a broader context, the recycling of fluorochemicals towards a circular fluorine economy is a key sustainability goal. Fluorochemicals are commonly treated as ‘single use’ and disposed of after their lifespan^24^; methods for the recycling of waste E–F bonds into new substrates through metal-mediated approaches are, therefore, of clear fundamental benefit.

We hypothesized that anionic calcium fluorides of the type [L_*n*_(X)_2_CaF]^−^ might represent more effective sources of nucleophilic fluoride than neutral or cationic counterparts (for example, **I**–**III**), based on electrostatic charge. Sterically encumbered multidentate L_*n*_X_2_ ligand scaffolds were also deemed essential to minimize aggregation. With this in mind, we drew on our recent work on isoelectronic molecular aluminium oxides (for example, **IV**)^25^, which face problems similar to calcium fluoride, such as a high lattice energy and the propensity to aggregate (Δ*H*_L_(Al_2_O_3_) = +15,920 kJ mol^−^^1^)^8^. Here we set out to explore the use of analogous calcium complexes featuring dianionic NON-donor ligands for the generation of molecular fluorides (**1**; Fig. 1). At the outset, we desired (1) to develop synthetic methodologies for the isolation of hitherto unknown monomeric calcium fluoride complexes (that is, those containing a single Ca–F bond), (2) to explore the formation of these species from recycled fluorine sources containing C–F bonds and (3) to define structure–activity relationships for the nucleophilic fluoride delivery into E–F bonds from these complexes.

## Results and discussion

### Synthesis of dinuclear calcium fluorides

**Fig. 2:**
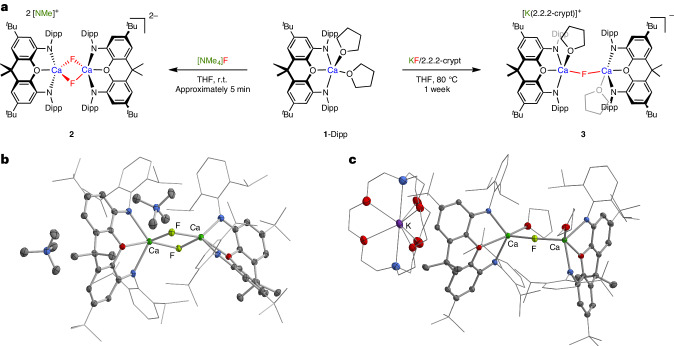
Synthesis and molecular structures of dinuclear calcium fluorides. **a**, Reaction of (^Dipp^NON)Ca(THF)_2_ (**1**-Dipp) with [NMe_4_]F in THF to generate [NMe_4_]_2_[{(^Dipp^NON)Ca(μ_2_-F)}_2_] (**2**) or with KF/2.2.2-crypt to yield [K(2.2.2-crypt)][{(^Dipp^NON) Ca(THF)}_2_(μ_2_-F)] (**3**) featuring Ca(μ_2_-F)_2_Ca and Ca(μ_2_-F)Ca cores, respectively. **b**,**c**, Molecular structures in the solid state of **2** (**b**) and **3** (**c**), respectively, as determined by X-ray crystallography. The thermal ellipsoids are set at the 30% level. Hydrogen atoms, second disorder components and solvate molecules are omitted, and selected fragments are shown in wireframe format for clarity. Structure **2** is displayed for connectivity purposes only. The selected bond lengths and angles for **3** are: Ca–F 2.182(1) and 2.187(1) Å and Ca–F–Ca 166.9(1)°.

NON-stabilized calcium systems (^Ar^NON)Ca(THF)_2_ (**1**, where ^Ar^NON is a chelating tridentate ligand of the type 4,5-bis(anilido)-2,7-di-*tert*-butyl-9,9-dimethylxanthene) are readily accessible from Ca(HMDS)_2_(THF)_2_ (where HMDS^−^ is {N(SiMe_3_)_2_}^−^) and the corresponding diprotio ligand (Fig. 1 and Supplementary Figs. 1 and 2)^26^. Complexes **1**-Dipp (Ar = Dipp, where Dipp is 2,6-^*i*^Pr_2_C_6_H_3_) and **1**-Trip (Ar = Trip, where Trip is 2,4,6-^*i*^Pr_3_C_6_H_2_) offer variation in the steric profile of the ligand scaffold and can be employed in the synthesis of CaF-containing complexes from a range of fluoride sources in organic media. For example, **1**-Dipp reacts with dry [NMe_4_]F in THF at room temperature over several minutes to generate one major product with a signal at *δ*_F_ = −70.4 ppm (Fig. 2a). The chemical shift is reminiscent of previously reported dimeric complexes featuring a Ca(μ_2_-F)_2_Ca core (for example, *δ*_F_ = −78 and −74 ppm for **I** and **II**, respectively)^18,19^ and the presence of a similar unit in the solid state structure of the product, [NMe_4_]_2_[{(^Dipp^NON)Ca(μ_2_-F)}_2_] (**2**), has been confirmed by X-ray crystallography (Fig. 2b).

The dimeric structure of **2** is based around a pair of five-coordinate calcium centres linked via two bridging fluoride ligands. The geometries at calcium are in between square pyramidal (SP) and trigonal bipyramidal (TBP) (τ = 0.59 and 0.61, respectively)^27^ with the two fluorides occupying the axial and one basal site in the SP limit. In the solid state, the [NMe_4_]^+^ cations are situated close to the [{(^Dipp^NON)Ca(μ_2_-F)}_2_]^2−^ units and are partially encapsulated between the flanking Dipp groups of opposing NON ligands, presumably to provide additional thermodynamic stabilization of the dimeric dianion. Attempts to cleave the dinuclear structure of **2** to generate monomeric systems of the type [(^Dipp^NON)Ca(L)F)]^−^ by adding a strong donor (for example, carbene or pyridine ligands) proved unsuccessful (Supplementary Fig. 3).

To probe alternative sources of fluoride, we also examined the reactivity of **1**-Dipp towards potassium fluoride in THF (in the presence of one equivalent of the K^+^ sequestering agent 2.2.2-cryptand; Fig. 2a). The reaction proceeds slowly over approximately 1 week at reflux to generate a single fluorine-containing product characterized by a higher field signal at *δ*_F_ = −87.3 ppm. Crystallization from benzene yields [K(2.2.2-crypt)][{(^Dipp^NON)Ca(THF)}_2_(μ_2_-F)] (**3**), as evidenced by single crystal X-ray diffraction (Fig. 2c). In contrast to **2**, **3** features a single bridging fluoride ligand between two calcium centres. The much lower solubility of the fluoride ion source presumably prevents the formation of the Ca(μ_2_-F)_2_Ca motif via the uptake of a second equivalent of F^−^. A THF molecule at each metal centre completes the five-coordinate geometry situated between SP and TBP (*τ* = 0.36 and 0.54, respectively). The Ca–F bond lengths (2.182(1) and 2.187(1) Å) are not significantly different from those found in Ca(μ_2_-F)_2_Ca units (for example, 2.170(2) and 2.189(2) Å for **I**)^18^; although, the geometry at the bridging fluoride ligand is much closer to linear (∠(Ca–F–Ca) = 166.9(1)°, cf. 76.7(1)° in **2**). The combination of a single bridging fluoride and a near linear geometry allows for rotational freedom of the bulky NON ligands, as evidenced by variable temperature nuclear magnetic resonance (VT-NMR) measurements (Supplementary Figs. 4 and 5).

### Synthesis of mononuclear calcium fluorides

**Fig. 3:**
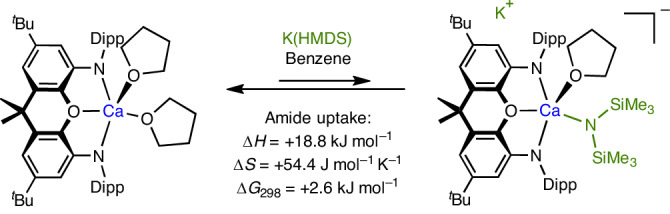
Equilibrium between 1-Dipp, K(HMDS) and the calcium amide complex K[(^Dipp^NON)Ca(THF)_*n*_(HMDS)] in benzene. The thermodynamic parameters (Δ*H*, Δ*S* and Δ*G*_298K_) associated with amide uptake are derived from a Van’t Hoff analysis of integrated ^1^H NMR signal intensities (in the temperature range of 25–70 °C).

Roesky and co-workers have previously shown that amide/fluoride metathesis represents a viable synthetic route for the formation of Ca–F bonds; the dimeric fluoride compound **I** was formed from {HC(MeCDippN)_2_}Ca(THF)(HMDS) and Me_3_SnF (with accompanying generation of Me_3_Sn(HMDS); Fig. 1)^18^. As such, we examined the reactivity of **1**-Dipp towards K(HMDS), as a possible route for the formation of an analogous (in this case anionic) calcium amide of the type [(^Dipp^NON)Ca(THF)_*n*_(HMDS)]^−^ (*n* = 0, 1). This reaction, carried out in benzene(-d_6_), leads to partial conversion to the desired amide complex K[(^Dipp^NON)Ca(THF)_*n*_(HMDS)] (Fig. 3a and Supplementary Fig. 6). Analysis of the equilibrium mixture as a function of temperature (25–70 °C) allows the thermodynamic parameters associated with substitution of THF by the HMDS anion to be evaluated via a Van’t Hoff plot (Supplementary Fig. 7). These data (Δ*H* = +18.8 kJ mol^−^^1^, Δ*S* = +54.4 J mol^−^^1^ K^−^^1^, Δ*G*_298_ = +2.6 kJ mol^−^^1^) are consistent with the position of equilibrium lying predominantly to the left at room temperature (approximately 25% adduct). In the case of Cs(HMDS), by contrast, the equilibrium lies much further to the right at room temperature (approximately 80% for 1 equiv. of Cs(HMDS), Supplementary Fig. 8) allowing for crystallization of the product. The obtained crystal structure (Supplementary Fig. 9) confirms the formation of the targeted amide adduct and features the Cs^+^ counterions sandwiched between the π systems of the NON ligand and the Dipp group of a second molecule.

**Fig. 4:**
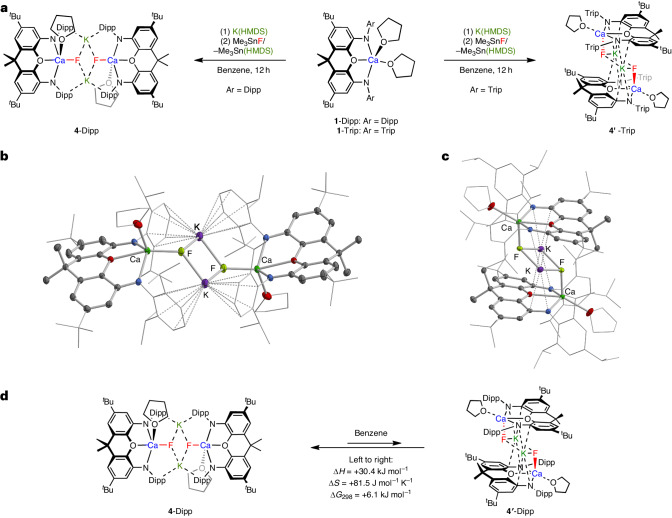
Synthesis and molecular structures of mononuclear calcium fluorides 4-Dipp and 4-Trip and how they are structurally connected. **a**, Syntheses of [K(^Dipp^NON)Ca(THF)F]_2_ (**4**-Dipp) and [K(^Trip^NON)Ca(THF)F]_2_ (**4′**-Trip) from **1**-Dipp or **1**-Trip via amide uptake and subsequent metathesis with Me_3_SnF. **b**,**c**, Centrosymmetric molecular structures in the solid state of **4**-Dipp (**b**) and **4′**-Trip (**c**), as determined by X-ray crystallography. The thermal ellipsoids are set at the 30% level. Hydrogen atoms, second disorder component and solvate molecule omitted and selected fragments are shown in the wireframe format for clarity. Selected bond lengths for **4**-Dipp are: Ca–F 2.151, K···F 2.560(1) and 2.634(1) Å and for **4′**-Trip are: Ca–F 2.143(1), K···F 2.526(2) and 2.532(1) Å. **d**, Equilibrium between **4**-Dipp and **4′**-Dipp in benzene solution; the thermodynamic parameters associated with the formation of the ‘stacked’ isomer **4′**-Dipp are derived from a Van’t Hoff analysis of integrated ^19^F NMR signal intensities (in the temperature range of 25–70 °C).

Despite the relatively low proportion of K[(^Dipp^NON)Ca(THF)_*n*_(HMDS)] present in solution at equilibrium, this system proves to be effective for the generation of Ca–F bonds from Me_3_SnF (Fig. 4a). The calcium-containing product formed from this sequential reaction at room temperature gives rise to one major NON environment (by ^1^H NMR) and a ^19^F NMR resonance at an even more upfield shift (*δ*_F_ = −97.8 ppm) than for **2** and **3**. The crystallized product (Fig. 4b) defines a dimeric entity [K(^Dipp^NON)Ca(THF)F]_2_ (**4**-Dipp), in which two formally anionic [(^Dipp^NON)Ca(THF)F]^−^ units are held together by potassium cations. The K^+^ ions interact both with the calcium-bound fluoride and the π-systems of the flanking Dipp groups (*d*(K···F) = 2.560(1) and 2.634(1) Å; *d*(K···C) = 3.335(2)–3.505(2) Å). This structural motif is very similar to those observed both for the valence-isoelectronic aluminium oxide system [K(^Dipp^NON)Al(THF)O)]_2_ reported previously by us and the calcium hydride complex [K(^Dipp^NON)Ca(OEt_2_)H)]_2_ synthesized by Hicks and co-workers^25,26^.

The smaller size of the fluoride ligand (compared with HMDS) presumably accounts for the more robust nature of the [(^Dipp^NON)Ca(THF)F]^−^ unit in **4**-Dipp compared with the amide complex [(^Dipp^NON)Ca(THF)(HMDS)]^−^. Moreover, the amide/fluoride metathesis approach utilizing Me_3_SnF appears to generate (in effect) a higher local concentration of fluoride in the vicinity of the calcium centre than the use of KF/2.2.2-cryptand. As a result, a 1:1 ratio of Ca^2+^ to F^−^ is achieved, in contrast to the 2:1 ratio in complex **3**. Although still dimeric in nature, **4**-Dipp represents the first example avoiding the formation of a Ca–F–Ca bridging unit. A comparison of the Ca–F (2.151(1) Å) and K···F (2.560(1) and 2.634(1) Å) distances in the solid state with the respective covalent radii (*r*_cov_(K) = 2.03 Å and *r*_cov_(Ca) = 1.76 Å) shows that **4**-Dipp features predominantly a Ca–F interaction supported by weaker K···F contacts^28^. This comparison is supported by quantum theory of atoms in molecules (QTAIM) analysis, demonstrating a higher charge density at the bond critical point along the Ca–F bond path compared with its K–F counterparts (Supplementary Fig. 10). Consistent with this hypothesis, the isostructural caesium compound [Cs(^Dipp^NON)Ca(THF)F]_2_ (**5**; Supplementary Fig. 11) contains only a marginally shortened Ca–F bond (2.137(2) Å), despite having markedly elongated Cs···F interactions (2.845(3) and 2.908(2) Å) compared with the potassium analogue.

The close approach of the CH groups in the *para* positions of the flanking Dipp groups in the solid-state structure of **4**-Dipp (*d*(C···C) = 3.376(3) Å; Fig. 4b) prompted us to examine the related complex **1**-Trip. We hypothesized that the additional ^*i*^Pr group in the *para* position would offer a steric impediment to the formation of the centrosymmetric ‘head-to-head’ dimer, thereby providing access to an alternative calcium fluoride motif. With this in mind, we exposed **1**-Trip to the same combination of K(HMDS) and Me_3_SnF in benzene, finding that the product is characterized by a more downfield ^19^F signal (*δ*_F_ = −87.5). Interestingly, while **4′**-Trip is shown crystallographically to possess a dimeric structure with similar Ca-F and K···F distances (*d*(Ca–F) = 2.143(1) Å, *d*(K···F) = 2.526(2) and 2.532(1) Å), the mode of aggregation is different to that in **4**-Dipp (Fig. 4c). Consistent with the expected effects of increased steric bulk in the NON-substituents, head-to-head dimerization is prevented, and **4′**-Trip features an alternative motif constructed by stacking two [(^Trip^NON)Ca(THF)F]^−^ fragments. The geometry at Ca in both **4**-Dipp and **4′**-Trip is intermediate between SP and TBP (τ = 0.54, 0.37, respectively) as for **2** and **3**; the contrasting structural motifs result in the occupation of different ligand sites by F^−^ and THF. Assuming the SP limit, F^−^ resides in a basal position in **4**-Dipp, allowing for ‘head-to-head’ dimerization with the K^+^ ions located close to the basal plane. In **4′**-Trip, by contrast, the fluoride resides in the axial position, facilitating a ‘stacked’ dimerization motif in which the K^+^ ions are sandwiched between two [(NON)Ca(THF)F]^−^ units.

When single crystalline samples of **4**-Dipp are dissolved in the benzene two ^19^F NMR resonances are observed—a major signal at *δ*_F_ = −97.8 ppm and a minor one (approximately 10% at 25 °C) at *δ*_F_ = −87 ppm. Given the similarity in chemical shift to **4′**-Trip, we hypothesize that the minor component in solution is the corresponding ‘stacked’ dimeric isomer of **4**-Dipp (that is, **4′**-Dipp; Fig. 4d). In line with this assertion, variable temperature and exchange spectroscopy (EXSY) NMR studies (Supplementary Figs. 12–15) demonstrate that the two species are in equilibrium, with thermodynamic parameters (Supplementary Fig. 13; Δ*H* = +30.4 kJ mol^−^^1^, ΔS = +81.5 J mol^−^^1^ K^−^^1^, Δ*G*_298_ = +6.1 kJ mol^−^^1^) consistent with an increasing proportion of **4′**-Dipp at elevated temperatures. This equilibrium is shifted markedly in favour of the ‘stacked’ isomer upon replacing benzene with THF (approximately 80% ‘stacked’).

**Fig. 5:**
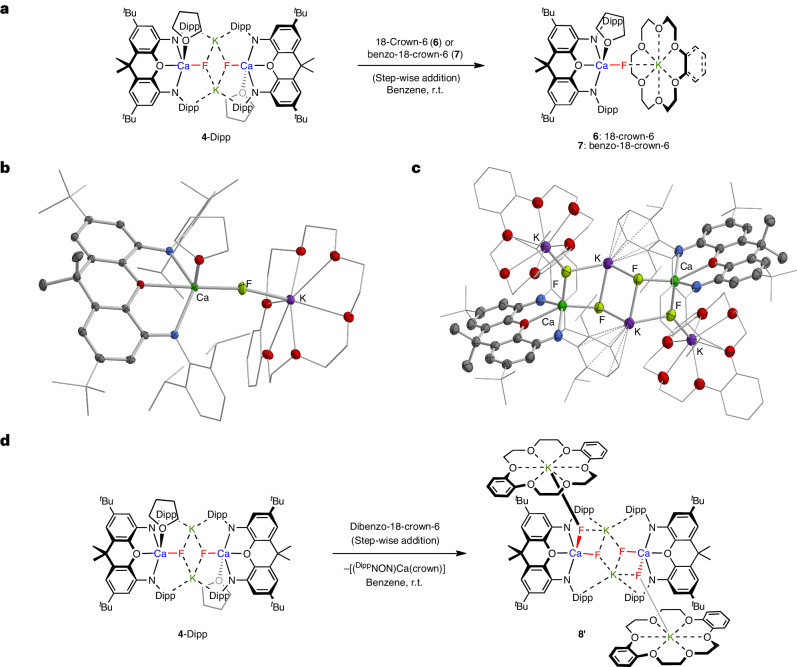
Synthesis and molecular structures of mononuclear calcium mono- (6 and 7) and difluorides (8′). **a**, Syntheses of monomeric calcium fluoride complexes **6** and **7** via the reactions of dimeric **4**-Dipp with 18-crown-6 or benzo-18-crown-6, respectively. **b**,**c**, Molecular structures in the solid state of one of the two independent molecules in the asymmetric unit of **7** (**b**) (see Supplementary Fig. 16 for **6**) and [K_2_(dibenzo-18-crown-6)(^Dipp^NON)CaF_2_]_2_ (**8′**) (**c**), as determined by X-ray crystallography. The thermal ellipsoids are set at the 30% level. Hydrogen atoms, solvate molecules and second disorder component are omitted, and selected fragments are shown in wireframe format for clarity. Selected bond lengths and angles for **7**: Ca–F 2.097(1) Å, K–F 2.434(1) Å and Ca–F–K 153.9(1)° (for the second molecule in the asymmetric unit: Ca–F 2.100(1) Å, K–F 2.425(1) Å and Ca–F–K 161.3(1)°); and for **8′**: Ca–F 2.149(3) and 2.177(3) Å, K···F 2.488(3) Å, 2.529(3) Å, 2.602(3) Å, 2.646(3) Å and F–Ca–F 91.9(1)°. **d**, Syntheses of dimeric calcium difluoride complex **8′** via the reaction of **4**-Dipp with dibenzo-18-crown-6.

To access calcium fluoride species of reduced nuclearity (that is, monomeric systems), abstraction of the K^+^ cation in **4**-Dipp was targeted. Attempts with the completely encapsulating cryptand 2.2.2-crypt were unsuccessful, leading to decomposition of the calcium fluoride complex. Hypothesizing that a degree of residual interaction with the K^+^ Lewis acid needs to be retained to prevent aggregation, the reactions of **4**-Dipp with the crown ethers 18-crown-6, benzo-18-crown-6 and dibenzo-18-crown-6 were investigated (Fig. 5a). All three systems give rise to one (similar) ^19^F NMR chemical shift in benzene (*δ*_F_ = −90.5, −88.8 and −89.3 ppm, respectively). Crystallography reveals very similar geometries for the former two products [K(18-crown-6)FCa(THF)(^Dipp^NON)] (**6**) and [K(benzo-18-crown-6)FCa(THF)(^Dipp^NON)] (**7**) (Fig. 5b and Supplementary Fig. 16). In each case, a monomeric [(^Dipp^NON)Ca(THF)F^−^ unit is linked to a [K(18-crown-6)]^+^/[K(benzo-18-crown-6)]^+^ cation through a single K···F interaction (again in line with QTAIM analysis; Supplementary Fig. 17). The K···F distances (for example, 2.434(1) Å in **7**) are shorter than in **4**-Dipp (2.560(1) and 2.634(1) Å), presumably because each fluoride ion is associated with only one (captured) K^+^ ion. Similarly, the distance between the calcium and fluoride ion in both **6** and **7** is markedly contracted compared with **4**-Dipp (for example, 2.100(1) and 2.097(1) Å in **7**).

For dibenzo-18-crown-6, the very similar ^19^F NMR shift and diffusion coefficient (diffusion ordered NMR spectroscopy, DOSY NMR) measured in solution (Supplementary Figs. 18 and 19) suggest a mononuclear product analogous to **6** and **7** (that is, [K(dibenzo-18-crown-6)FCa(THF)(^Dipp^NON)], **8**). By contrast, single crystalline material features the centrosymmetric dimer [K_2_(dibenzo-18-crown-6)(^Dipp^NON)CaF_2_]_2_ (**8′**; Fig. 5c,d) containing two formally dianionic [(^Dipp^NON)CaF_2_]^2−^ units (featuring a 1:2 Ca:F ratio). The basal fluorides engage in a bifurcated interaction with two arene-supported K^+^ counterions reminiscent of **4**-Dipp itself (*d*(Ca–F) = 2.149(3) Å, *d*(K···F) = 2.488(3) and 2.602 Å), while the apical fluorides each interact with [K(dibenzo-18-crown-6)]^+^ units in a manner analogous to that found in **6** and **7** ((*d*(Ca–F) = 2.177(3) Å, *d*(K···F) = 2.529(3) Å). A longer additional K···F contact (2.646(3) Å) completes a ladder-like Ca/F/K motif. The formation of this structural entity by fluoride redistribution between calcium centres upon crystallization is presumably driven by issues of (in)solubility, with **8′** resisting re-dissolution in benzene.

To our knowledge, **6** and **7** represent the first examples of mononuclear calcium fluoride complexes (that is, systems featuring a single Ca–F bond). Accordingly, their Ca–F bond lengths (for example, 2.100(1)/2.097(1) Å in **7**) are significantly shorter than any previously reported (approximately 2.17 Å for **I** and **II**, all containing Ca(μ_2_-F)_2_Ca cores; Supplementary Fig. 20)^18,19^. The Ca–F distances measured for **4**-Dipp and **4′**-Trip are also relatively short (2.151(1) and 2.143(1) Å) being significantly smaller than the sum of the respective covalent radii (1.76 + 0.57 = 2.33 Å)^28^. **8′** represents the first example of a molecular calcium difluoride complex containing a (five-coordinate) calcium centre with two fluorides (Ca–F lengths: 2.149(3) and 2.177(3) Å) in a *cis* configuration (F–Ca–F angle 91.9(1)°).

Spectroscopic solution data are also informative with respect to the nature of aggregation of calcium fluoride entities (Supplementary Fig. 21). Ca(μ_2_-F)_2_Ca systems possess ^19^F signals between −70 and −80 ppm (for example, −78, −74 and −70 ppm for **I,** **II** and **2**)^18,19^ while a singly bridged Ca(μ_2_-F)Ca motif (**3**) results in a more upfield shift (−87 ppm). Complexes featuring Ca–F···K_*n*_ units (rather than Ca–F–Ca) also induce higher field ^19^F NMR signals, be they monomeric (for example, −90 ppm for **5**) or dimeric (for example, −98 ppm for the ‘head-to-head’ dimer **4**-Dipp), although the precise geometry around the fluoride ion clearly exerts a secondary influence (for example, −87.5 ppm for the ‘stacked’ isomers **4′**-Trip and **4′**-Dipp).

### Calcium fluoride complexes from C–F bonds

While mononuclear calcium fluoride systems are now accessible, our synthetic route requires a non-renewable source of fluoride (that is, Me_3_SnF). Sourcing fluoride instead from C–F bonds offers potential sustainability benefits. Okuda and co-workers have previously shown that [(Me_4_TACD)_2_Ca_2_(μ-F)_2_(THF)][BAr_4_]_2_ can be synthesized from the analogous hydride and fluorobenzene at 60 °C in THF^22^. Given its previous identification as a challenging substrate^29^ and the abundance of fluoroarenes in pharmaceuticals and agrochemicals^3^, we attempted to exploit fluorobenzene in similar fashion as the source of fluoride in our calcium complexes. Accordingly, using hydride precursors of the type [K(^Dipp^NON)Ca(L)H]_2_ (L = Et_2_O or THF), **4**-Dipp can be synthesized selectively by the defluorination of fluorobenzene under very mild conditions (room temperature, benzene; Supplementary Figs. 22–25), thereby removing the dependency on non-sustainable fluoride sources.

### Reactivity studies

**Fig. 6:**
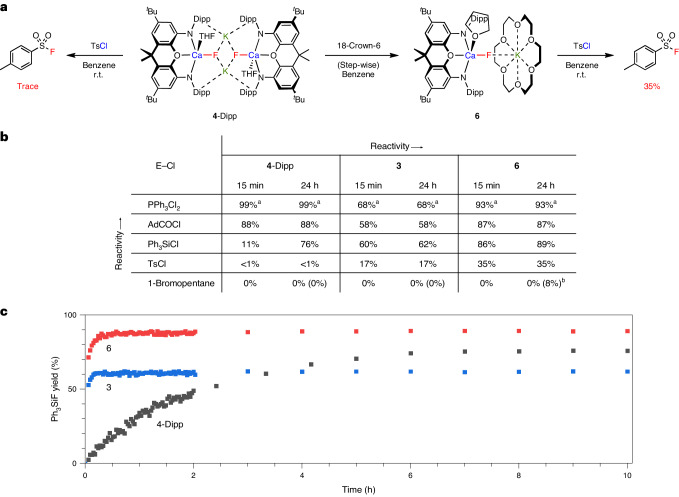
Comparative reactivity studies of 3, 4-Dipp and 6 with several electrophiles. **a**, Reactivity towards TsCl. **b**, Fluorination of C-, Si- and P-centred electrophiles. **c**, Temporal plot showing the conversion of Ph_3_SiCl to Ph_3_SiF by **3,** **4**-Dipp and **6**. All the yields were determined by quantitative ^19^F NMR spectroscopy with PhF as internal standard. The controls with KF or Me_3_SnF showed no appreciable product formation within the studied timeframe. The selected spectra can be found in Supplementary Figs. 26–40. ^a^The total amount of F in P–F bonds from a mixture of PPh_3_F_2_ and monofluorinated product. ^b^The yield in parentheses achieved at 80 °C.

The viability of calcium fluoride complexes as nucleophilic fluoride transfer agents has not previously been demonstrated. For example, while Okuda and co-workers demonstrated the abstraction of fluoride from fluorochemicals, no subsequent fluoride delivery was achieved^22^. With the idea of demonstrating fluorine repurposing, we therefore aim to deliver fluoride from complexes such as **4**-Dipp that can ultimately be synthesized by C–F defluorination. Additionally, given the several different structural motifs in hand, we set out to understand the influence of the fluoride environment on its reactivity towards E–F bond formation (Fig. 6). Given the insolubility of **2** in benzene, we focussed our comparison on the dinuclear Ca–F–Ca and di- and mononuclear Ca–F···K_*n*_-containing complexes (that is, **3**, **4**-Dipp and **6**, respectively). We initially focussed on the electrophile 4-toluenesulfonyl chloride (TsCl) considering the strong thermodynamic driver towards S–F bond formation (for example, approximately 380 versus 190 kJ mol^−^^1^ for S–X in SO_2_F_2_ and SO_2_Cl_2_, respectively)^27^, and their widespread synthetic utility^30,31^. Accordingly, the room temperature reaction of TsCl with **4**-Dipp resulted in only trace quantities of TsF, while the corresponding reaction with **3** and **6** led to the rapid formation of TsF in 17% and 35% yield over 15 min (Fig. 6a). With this particular electrophile, S–F bond formation competes with the reaction of TsCl with the ancillary NON ligand leading to decomposition of the complex, and no yield increase is observed at prolonged reaction times.

To explore the broader scope of E–F bond formation, fluoride transfer to C(sp^2^)-, C(sp^3^)-, Si- and P-based electrophiles was investigated (Fig. 6b). The more reactive PPh_3_Cl_2_ and AdCOCl result in high yields immediately after addition of either **3** (68% and 58%), **4**-Dipp (99% and 88%) or **6** (93% and 87% after 15 min, respectively). Over a longer period (24 h), the reaction with Ph_3_SiCl affords Ph_3_SiF in good yields (62%, 76% and 89%, respectively), offering a convenient timeframe for reaction rate comparison via in situ ^19^F NMR. The resulting temporal plot (Fig. 6c) shows that the reaction is largely complete after 15 min using either **3** or **6** as the F^–^ source (in 60 and 86% yield), whereas with **4**-Dipp the reaction takes more than 10 h to complete. The rapid conversion for complexes **3** and **6** compared with **4**-Dipp can be rationalized by the sterically accessible environment of the fluoride, having two metal interactions in a close to linear geometry (Ca–F–Ca and Ca–F···[K-18-crown-6]). In terms of the differing yields with **3** and **6**, competing reactions at the NON ligand (as observed explicitly with TsCl) are statistically more likely in the case of **3** (which has a higher (NON)Ca:F ratio). Finally, the much less reactive electrophile 1-bromopentane was explored in nucleophilic fluorination. While no reaction was observed with **3** and **4**-Dipp under any conditions examined, the formation of 1-fluoropentane from **6** was found to be feasible, albeit in low yield (8%) after heating to 80 °C for 24 h.

In summary, we have shown that anionic systems of the type [L_*n*_(X)_2_CaF]^−^ (in combination with weakly polarizing counterions) can be accessed via defluorination of fluorochemicals, and that these systems are competent for the delivery of fluoride to a range of electrophilic substrates. The monomeric calcium fluoride complexes, containing a single Ca–F bond, can be accessed using crown-ether co-ligands. A two coordinate, close to linear fluoride environment enables fast kinetics for nucleophilic fluoride transfer. When combined with a weak secondary K···F interaction, the optimal combination of both rate and yield is achieved. These synthetic approaches provide fundamental understanding of the molecular design features intrinsic in a calcium complex capable of abstracting and delivering F^−^, showing in principle how the F content of fluorochemicals can be repurposed to deliver a range of new E–F bonded products.

## Methods

All general considerations and synthetic procedures, along with compound characterization data, can be found in Supplementary Information.

## Online content

Any methods, additional references, Nature Portfolio reporting summaries, source data, extended data, supplementary information, acknowledgements, peer review information; details of author contributions and competing interests; and statements of data and code availability are available at 10.1038/s41557-024-01524-x.

## Supplementary information

Supplementary Information Materials and methods, Supplementary Figs. 1–81, X-ray crystallographic data and density functional theory data.

Supplementary Data 1 Crystallographic data for compound **1**-Dipp; CCDC reference 2282778.

Supplementary Data 2 Structure factor file for compound **1**-Dipp; CCDC reference 2282778.

Supplementary Data 3 Crystallographic data for compound **1**-Trip; CCDC reference 2282783.

Supplementary Data 4 Structure factor file for compound **1**-Trip; CCDC reference 2282783.

Supplementary Data 5 Crystallographic data for compound **2**; CCDC reference 2283075.

Supplementary Data 6 Structure factor file for compound **2**; CCDC reference 2283075.

Supplementary Data 7 Crystallographic data for compound **3**; CCDC reference 2282786.

Supplementary Data 8 Structure factor file for compound **3**; CCDC reference 2282786.

Supplementary Data 9 Crystallographic data for compound **4**-Dipp; CCDC reference 2282794.

Supplementary Data 10 Structure factor file for compound **4**-Dipp; CCDC reference 2282794.

Supplementary Data 11 Crystallographic data for compound **4**-Trip; CCDC reference 2282801.

Supplementary Data 12 Structure factor file for compound **4**-Trip; CCDC reference 2282801.

Supplementary Data 13 Crystallographic data for compound Cs[(^Dipp^NON)Ca(HMDS)]; CCDC reference 2282803.

Supplementary Data 14 Structure factor file for compound Cs[(^Dipp^NON)Ca(HMDS)]; CCDC reference 2282803.

Supplementary Data 15 Crystallographic data for compound **5**; CCDC reference 2282807.

Supplementary Data 16 Structure factor file for compound **5**; CCDC reference 2282807.

Supplementary Data 17 Crystallographic data for compound **6**; CCDC reference 2283071.

Supplementary Data 18 Structure factor file for compound **6**; CCDC reference 2283071.

Supplementary Data 19 Crystallographic data for compound **7**; CCDC reference 2282808.

Supplementary Data 20 Structure factor file for compound **7**; CCDC reference 2282808.

Supplementary Data 21 Crystallographic data for compound **8**; CCDC reference 2282811.

Supplementary Data 22 Structure factor file for compound **8**; CCDC reference 2282811.

Supplementary Data 23 Crystallographic data for compound **9**-THF; CCDC reference 2312195.

Supplementary Data 24 Structure factor file for compound **9**-THF; CCDC reference 2312195.

Supplementary Data 25 Crystallographic data for compound NONH_2_-IMe_4_; CCDC reference 2283072.

Supplementary Data 26 Structure factor file for compound NONH_2_-IMe_4_; CCDC reference 2283072.

Supplementary Data 27 XYZ files for compounds **4**-Dipp and **6**.

## Data Availability

The crystallographic data for the structures reported in this article have been deposited at the Cambridge Crystallographic Data Centre (CDCC), under deposition numbers CCDC 2282778 (compound **1**-Dipp), 2282783 (compound **1**-Trip), 2282786 (compound **3**), 2282794 (compound **4**-Dipp), 2282801 (compound **4**-Trip), 2282803 (compound Cs[(DippNON)Ca(HMDS)]), 2282807 (compound **5**), 2282808 (compound **7**), 2282811 (compound **8**), 2283071 (compound **6**), 2283072 (compound NONH_2_–IMe_4_), 2283075 (compound **2**) and 2312195 (compound **9**-THF). Copies of the data can be obtained free of charge from www.ccdc.cam.ac.uk/structures/. All other data supporting the findings of this study are available within the article and its Supplementary Information, at the Oxford University Research Archive (https://ora.ox.ac.uk), and from the corresponding authors upon reasonable request.
