# Supplementary material for: Enabling nucleophilic reactivity in molecular calcium fluoride complexes
Source: Nat Chem. 2024 May 14;16(9):1473–80. doi: 10.1038/s41557-024-01524-x (PMC11375610; doi:10.1038/s41557-024-01524-x)
Supplement: Supplementary file 1 — Materials and methods, Supplementary Figs. 1–81, X-ray crystallographic data and density functional theory data. [file 41557_2024_1524_MOESM1_ESM.pdf]

nature chemistry

Article

https://doi.org/10.1038/s41557-024-01524-x

Enabling nucleophilic reactivity in molecular calcium fluoride complexes

In the format provided by the authors and unedited

Table of Contents

Materials and methods 3

General considerations 3

Synthetic, spectroscopic, and analytical data 4

Synthesis of Me_3SnF 4

Synthesis of $Ca(HMDS)_2(thf)_2$ 4

Synthesis of Cs(HMDS) 4

Synthesis of (^{Dipp}NON)$Ca(thf)_2$ (1-Dipp) 5

Synthesis of (^{Trip}NON)$Ca(thf)_2$ (1-Trip) 5

Synthesis of $[NMe_4]_2$ [{(^{Dipp}NON)Ca(μ_2-F)}$_2$] (2) 6

Synthesis of [K(2.2.2-crypt)] [{(^{Dipp}NON)Ca(thf)}$_2$(μ_2-F)] (3) 6

Synthesis of [K(^{Dipp}NON)CaF(thf)]$_2$ (4-Dipp) 6

Synthesis of [K(^{Trip}NON)CaF(thf)]$_2$ (4'-Trip) 7

Synthesis of [Cs(^{Dipp}NON)CaF(thf)]$_2$ (5) 7

Synthesis of (^{Dipp}NON)CaF(thf)(K 18-crown-6) (6) 8

Synthesis of (^{Dipp}NON)CaF(thf)(K benzo-18-crown-6) (7) 8

Synthesis of [(K dibenzo-18-crown-6)K(^{Dipp}NON)CaF_2]$_2$ (8) 9

Supplementary data 10

Reactivity studies 21

Defluorination of fluorobenzene 21

Nucleophilic fluoride transfer 24

NMR spectra of synthesized compounds 34

Me_3SnF 34

$Ca(HMDS)_2(thf)_2$ 36

Cs(HMDS) 38

1-Dipp 41

1-Trip 43

2 45

3 48

4-Dipp and 4'-Dipp 51

4'-Trip 54

5 57

6 60
7 63
8 66
X-ray crystallographic data 71
References 77

Materials and methods

General considerations

All manipulations were carried out using standard Schlenk line or dry-box techniques under an atmosphere of argon or dinitrogen. Solvents were degassed by sparging with argon and dried by passing through a column of the appropriate drying agent and stored over potassium. NMR spectra were measured in benzene-d_6, which was dried over potassium, distilled under reduced pressure and stored over potassium in Teflon valve ampoules. NMR samples were prepared under argon or dinitrogen in 5 mm Wilmad 507-PP tubes fitted with J. Young Teflon valves. ^{1}H, ^{19}F, $^{13}C\{^{1}H\}$ and ^{133}Cs NMR spectra were recorded on Bruker Avance III HD nanobay 400 MHz, Bruker Avance III 500 MHz or Bruker Avance III HD 600 MHz spectrometer at ambient temperature unless stated otherwise and referenced internally to residual protio-solvent (^{1}H) or solvent (^{13}C) resonances and are reported relative to tetramethylsilane (δ = 0 ppm). ^{19}F resonances are referenced externally to $CFCl_3$. Assignments were confirmed using ^{1}H-^{1}H COSY and NOESY, and ^{13}C-^{1}H HSQC and HMBC NMR correlation experiments. Chemical shifts are quoted in δ (ppm) and coupling constants in Hz. Elemental analyses were carried out by London Metropolitan University. $^{Dipp}NONH_2$[1], $^{Trip}NONH_2$[2], $[K(^{Dipp}NON)CaH(thf)]_2$[3] and $[K(^{Dipp}NON)CaH(OEt)]_2$[3] were prepared according to (slightly modified) literature procedures. All other reagents were used as received.

Single-crystal X-ray diffraction data were collected using either using a Rigaku Supernova dual-source diffractometer (compounds 1-Dipp, 1-Trip, 2, 3, 4-Dipp, 8') or a Rigaku Xcaliber single source diffractometer (compounds 4-Trip, 5, 6, 7). Crystals were selected under Paratone-N oil, mounted on Micromount loops and quench-cooled using an Oxford Cryosystems open flow N_2 cooling device. Data were collected at 150 K (or 100 K for 1-Trip, 4-Trip, 5, 7 or 200 K for 6) using mirror monochromated Cu Kα (λ = 1.5418 Å) or Mo Kα (λ = 0.71073 Å) radiation. Structure **6** was collected at a higher temperature (200 K) as the onset of a phase transition occurs proceeding to lower temperatures (at ca. 190 K). Data collected were processed using the CrysAlisPro package, including unit cell parameter refinement and inter-frame scaling (which was carried out using SCALE3 ABSPACK within CrysAlisPro). Equivalent reflections were merged and diffraction patterns processed with the CrysAlisPro suite. Structures were subsequently solved using SHELXT-2018 and refined on F^2 using the SHELXL 2018 package and the graphical interface Olex2. The refraction data for structure **2** contains a modulated component leading to poor refinement parameters (see Figure S59); **2** is displayed for connectivity purposes only. Finalized CIFs for all X-ray diffraction structures (2282778, 2282783, 2282786, 2282794, 2282801, 2282803, 2282807, 2282808, 2282811, 2283071, 2283072, 2283075, 2312195) have been deposited at the Cambridge Crystallographic Data Centre. These can be obtained free-of-charge via www.ccdc.cam.ac.uk/data_request/cif, by emailing data_request@ccdc.cam.ac.uk, or by contacting The Cambridge Crystallographic Data Centre, 12 Union Road, Cambridge CB2 1EZ, UK; fax: +44 1223 336033.

All computational work reported here was performed using ORCA (Revision 5.0.4)[4–6]. The meta-generalized-gradient approximation (mGGA) functional R^2-SCAN[7,8] was employed in

conjunction with the Def2-TZVPPm[9] basis set with the D4 dispersion correction[10,11], and employing the geometrical counterpoise correction gCP[12] (together known as the R^2-SCAN-3c method)[9]. The nature of the stationary points (minima) was confirmed by full frequency calculations and are characterized by zero imaginary frequencies. Wavefunction analysis was performed using NBO7, Multiwfn and AIMALL[13–15].

Synthetic, spectroscopic, and analytical data

Synthesis of Me_3SnF
A solution of KF (4.36 g, 75.0 mmol) in H_2O (10 ml) was added to a stirred solution of Me_3SnCl (9.96 g, 50 mmol). The resulting precipitation was filtered off and dried under reduced pressure. The crude product was subsequently purified by sublimation (~170 °C) to result in Me_3SnF (7.32 g, 80%)[16].

^{1}H NMR (400 MHz, CD_3OD, 298 K) δ 0.45 (s, 3H) ppm.

^{19}F NMR (377 MHz, CD_3OD, 298 K) δ -165.71 (s, 1F) ppm.

Spectroscopic data are in accordance with those in literature (^{1}H NMR)[17].

Synthesis of $Ca(HMDS)_2(thf)_2$
CaI_2 (11.76 g, 40.0 mmol) and K(HMDS) (15.96, 80.0 mmol) were charged to a Schlenk flask with stirrer bar. After the addition of thf (200 ml), the solution was stirred overnight (~15h) at room temperature. After evaporation of the solvent under reduced pressure, the solids were extracted with 4 portions of hexane (250 ml total). The obtained solution was reduced in volume (~60 ml) from which crystals of the product were grown, filtered and dried under reduced pressure. The remaining solution was subjected to further crystallizations to result in a 86% total yield of $Ca(HMDS)_2(thf)_2$ (17.3 g)[18].

^{1}H NMR (400 MHz, C_6D_6, 298 K) δ 3.62 – 3.52 (m, 8H), 1.26 (m, 8H), 0.36 (s, 36H) ppm.

^{13}C NMR (101 MHz, C_6D_6, 298 K) δ 69.8, 25.0, 5.9 ppm.

Spectroscopic data are in accordance with those in literature[18].

Synthesis of Cs(HMDS)
To an ampoule with stirrer bar were added CsF (3.04 g, 20.0 mmol) and Li(HMDS) (3.35 g, 20.0 mmol). After the ampoule was cooled by a dry ice/acetone bath, hexane (40 ml) was slowly added. The suspension was stirrer overnight (~17 h) at reflux. The solvent was removed under reduced pressure. The remaining solids were extracted with toluene (3x30 ml) and filtered into another Schlenk flask. The toluene of combined extracts was subsequently removed resulting in the crude product. The crude solid was further purified by sublimation (200 °C, ~3 h) resulting in pure Cs(HMDS) (3.91 g, 75%).

^{1}H NMR (400 MHz, C_6D_6, 298 K) δ 0.22 (s, 18H) ppm.

^{13}C NMR (101 MHz, C_6D_6, 298 K) δ 7.3 ppm.

^{133}Cs NMR (65.6 MHz, C_6D_6, 298 K) δ 125.7 ppm.

Spectroscopic data are in accordance with those in literature[19].

Synthesis of (^{Dipp}NON)Ca(thf)$_2$ (1-Dipp)

To a Schlenk flask equipped with stirrer bar was added ^{Dipp}NONH$_2$ (2.02 g, 3.0 mmol) and Ca(HMDS)$_2$(thf)$_2$ (1.52 g, 3.0 mmol) and 40 ml of thf. The resulting solution was stirred at 50 °C overnight (17 h). After partial evaporation of the solvent under reduced pressure, crystals of the product were grown at 4 °C, filtered and dried under reduced pressure. The remaining solution was subjected to further crystallizations to result in an 81% total yield of **1-Dipp** (2.08 g).

^{1}H NMR (400 MHz, C_6D_6, 298 K) δ 7.28 (d, *J* = 7.5 Hz, 4H), 7.16 (t, *J* = 7.6 Hz, 2H), 6.65 (d, *J* = 2.2 Hz, 2H), 6.22 (d, *J* = 2.2 Hz, 2H), 3.61 (hept, *J* = 6.9 Hz, 4H), 3.24 – 3.11 (m, 8H), 1.80 (s, 6H), 1.38 (s, 18H), 1.35 (d, *J* = 6.8 Hz, 12H), 1.12 (d, *J* = 6.9 Hz, 12H), 1.10 – 1.05 (m, 8H) ppm.

^{13}C NMR (101 MHz, C_6D_6, 298 K) δ 149.7, 148.9, 146.6, 145.4, 139.1, 129.5, 123.7, 122.6, 107.6, 103.3, 69.1, 35.4, 35.0, 32.2, 31.9, 28.1, 25.5, 25.4, 25.2 ppm.

Elemental analysis: anal. calc. for $C_{55}H_{78}CaN_2O_3$: C 77.24%, H 9.19%, N 3.28%, found: C 77.32%, H 9.20%, N 3.45%.

Spectroscopic data are in accordance with those in literature[3].

Synthesis of (^{Trip}NON)Ca(thf)$_2$ (1-Trip)

To a Schlenk flask equipped with stirrer bar was added ^{Trip}NONH$_2$ (757 mg, 1.0 mmol) and Ca(HMDS)$_2$(thf)$_2$ (505 mg, 1.0 mmol) and 20 ml of thf. The resulting solution was stirred at 50 °C overnight (18 h). After removal of the solvent under reduced pressure, the solid was dissolved in benzene and the solution was filtered through a glass fibre cannula filter into a Y-shaped Young tube. The solution was degassed via dynamic reduced pressure and crystals of the product were grown by slow condensation of solvent into the side arm at RT. The liquid in the side arm was removed by cannula after which the crystals of **1-Trip** (70%, 661 mg) were dried under reduced pressure and transferred to the glovebox.

^{1}H NMR (400 MHz, C_6D_6, 298 K) δ 7.20 (s, 4H), 6.64 (d, *J* = 2.2 Hz, 2H), 6.25 (d, *J* = 2.2 Hz, 2H), 3.63 (hept, *J* = 6.9 Hz, 4H), 3.16 (s, 8H), 2.91 (hept, *J* = 7.0 Hz, 2H), 1.80 (s, 6H), 1.41 (d, *J* = 6.8 Hz, 12H), 1.37 (s, 18H), 1.28 (d, *J* = 6.9 Hz, 12H), 1.16 (d, *J* = 7.0 Hz, 12H), 1.14 – 1.09 (m, 8H) ppm.

^{13}C NMR (101 MHz, C_6D_6, 298 K) δ 149.0, 147.2, 146.5, 145.1, 142.1, 139.2, 129.3, 121.3, 107.5, 103.1, 69.1, 35.3, 35.0, 34.6, 32.2, 31.9, 28.1, 25.7, 25.5, 25.2, 24.8 ppm.

Elemental analysis: anal. calc. for $C_{61}H_{90}CaN_2O_3$: C 77.99%, H 9.66%, N 2.98%, found: C 78.15%, H 9.70%, N 2.77%.

Synthesis of [NMe$_4$]$_2$ [{(^{Dipp}NON)Ca(μ$_2$-F)}$_2$] (2)
1-Dipp (25.7 mg, 0.030 mmol), TMAF (2.8 mg, 0.030 mmol), and 0.5 ml thf-d$_8$ were added to an J. Young NMR tube. The solution was sonicated to dissolve all reactants and let to react for 4 h after which quantitative conversion to product **2** was observed. Single crystals of **2** for XRD were grown by filtering the solution through a syringe filter into a Y-shaped Young tube, degassing the solution via dynamic reduced pressure and slow condensation of solvent into the side arm at RT.

^{1}H NMR (500 MHz, thf-d$_8$, 298 K) δ 6.97 (d, *J* = 7.6 Hz, 8H), 6.78 (t, *J* = 7.6 Hz, 4H), 6.08 (d, *J* = 2.2 Hz, 4H), 5.55 (d, *J* = 2.3 Hz, 4H), 3.49 (hept, *J* = 6.9 Hz, 8H), 2.89 (s, 24H), 1.55 (s, 12H), 1.07 (d, *J* = 7.1 Hz, 24H), 1.05 (s, 36H), 1.00 (d, *J* = 7.0 Hz, 24H) ppm.

^{19}F NMR (377 MHz, thf-d$_8$, 298 K) δ -70.41 ppm.

^{13}C NMR (126 MHz, thf-d$_8$, 298 K) δ 152.0, 150.2, 146.0, 145.6, 140.2, 129.2, 123.6, 121.6, 107.1, 100.6, 55.4, 35.6, 35.3, 32.5, 32.5, 31.7, 28.1, 25.7 ppm.

Synthesis of [K(2.2.2-crypt)] [{(^{Dipp}NON)Ca(thf)}$_2$(μ$_2$-F)] (3)
1-Dipp (25.7 mg, 0.030 mmol), KF (0.9 mg, 0.015 mmol), 2.2.2-Cryptand (5.6 mg, 0.015 mmol), and 0.5 ml thf-d$_8$ were added to an J. Young NMR tube. The solution was heated at reflux to reach full conversion to **3** as indicated by ^{1}H NMR (ca. 1 week). Single crystals of **3** for XRD were grown by filtering the solution through a syringe filter into a Y-shaped Young tube, degassing the solution via dynamic reduced pressure, further concentrating the solution, and slow condensation of solvent into the side arm at RT at 4 °C.

^{1}H NMR (400 MHz, thf-d$_8$, 333 K) δ 6.91 (d, *J* = 7.6 Hz, 8H), 6.75 (t, *J* = 7.6 Hz, 4H), 6.10 (d, *J* = 2.2 Hz, 4H), 5.56 (d, *J* = 2.2 Hz, 4H), 3.53 (s, 12H), 3.49 (t, J = 4.6 Hz, 12H), 3.35 (hept, *J* = 6.9 Hz, 8H), 2.52 (t, *J* = 4.7 Hz, 12H), 1.54 (s, 12H), 1.04 (s, 36H), 0.95 (d, *J* = 6.9 Hz, 24H), 0.89 (d, *J* = 6.8 Hz, 24H) ppm.

^{19}F NMR (377 MHz, thf-d$_8$, 298 K) δ -87.32 ppm.

^{13}C NMR (101 MHz, thf-d$_8$, 298 K) δ 152.3, 150.1, 145.8, 145.1, 140.9, 130.2, 123.6, 121.6, 108.2, 100.9, 71.4, 68.5, 54.8, 35.7, 35.1, 32.4, 31,9, 28.4, 25.6, 25.5 ppm.

Synthesis of [K(^{Dipp}NON)CaF(thf)]$_2$ (4-Dipp)
1-Dipp (342 mg, 0.4 mmol), K(HMDS) (79.8 mg, 0.4 mmol), and Me_3SnF (73.1 mg, 0.4 mmol) were added to a Schlenk flask with stirrer bar. After the addition of 20 ml benzene, the mixture was stirred overnight (13 h). The mixture was filtered through a glass fibre cannula filter into a Y-shaped Young tube. The solution was degassed via dynamic reduced pressure and crystals of the product were grown by slow condensation of solvent into the side arm at RT. The colourless crystals were washed by condensing back small amounts of benzene into

the main arm and decanting of the green-yellowish solution until colourless crystals were obtained. The liquid in the side arm was removed by cannula after which the crystals of **4-Dipp** (96%, 324 mg) were transferred to the glovebox (removal of all volatile components under reduced pressure leads to decomposition).

^{1}H NMR (400 MHz, C_6D_6, 298 K) 7.16 (d, *J* = 7.5 Hz, 8H) δ 7.08 (t, *J* = 7.5 Hz, 4H), 6.66 (d, *J* = 2.2 Hz, 4H), 6.57 (d, *J* = 2.2 Hz, **4'-Dipp**), 6.16 (d, *J* = 2.1 Hz, 4H), 6.08 (br s, **4'-Dipp**), 3.69 – 3.62 (m, 8H), 3.59 (hept, *J* = 7.0 Hz, 8H), 1.79 (s, 12H), 1.36 (s, 36H), 1.33 – 1.27 (m, 24H), 1.25 – 1.20 (m, 8H), 1.08 – 0.74 (m, 24H) ppm.

^{19}F NMR (377 MHz, C_6D_6, 298 K) δ -87.51 (**4'-Dipp**), -97.76 (**4-Dipp**) ppm.

^{13}C NMR (126 MHz, C_6D_6, 298 K) δ 151.4, 148.4, 146.5, 145.7, 139.3, 129.6, 123.7, 122.2, 106.8, 103.5, 69.2, 35.5, 35.0, 32.2, 32.1, 27.7, 25.4, 25.1 ppm.

Elemental analysis: anal. calc. for $C_{102}H_{140}Ca_2F_2K_2N_4O_4$: C 72.81%, H 8.39%, N 3.33%, found: C 72.70%, H 8.58%, N 3.59%.

Synthesis of [K(^{Trip}NON)CaF(thf)]$_2$ (4'-Trip)

1-Trip (14.1 mg, 0.015 mmol), K(HMDS) (3.0 mg, 0.015 mmol), and Me_3SnF (2.7 mg, 0.015 mmol) were added to a to a J. Young NMR tube. All attempts towards (large scale) isolation for characterisation were unsuccessful. For NMR characterisation, spectra of the *in situ* reaction performed in 0.5 ml thf, resulting in a clean conversion to **4'-Trip** after 24 h, are provided. To obtain single crystalline material of **4'-Trip** for XRD, 0.5 ml benzene and 3 drops of thf (instead of 0.5 ml thf) were added and the mixture was monitored over time by ^{19}F NMR. The reaction was stopped when broad signals other than the product (δ_F = -87.5) started to grow in (ca. 30% yield of product). The solution was then filtered into a Y-shaped Young tube and crystals were obtained by slow evaporation of the solvent.

^{1}H NMR (400 MHz, thf-d_8, 298 K) δ 6.86 (s, 8H), 6.07 (d, J = 2.2 Hz, 4H), 5.61 (d, J = 2.1 Hz, 4H), 3.54 (m, 8H), 3.47 (hept, J = 6.8 Hz, 8H), 2.77 (hept, J = 6.8 Hz, 4H), 1.68 (m, 8H), 1.56 (s, 12H), 1.17 (d, J = 6.9 Hz, 24H), 1.07 (s, 36H), 1.07 (d, J = 6.8 Hz, 24H), 1.00 (d, J = 6.8 Hz, 24H).

^{19}F NMR (377 MHz, thf-d_8, 298 K) δ -87.32 ppm.

^{19}F NMR (377 MHz, C_6D_6, 298 K) δ -87.51 ppm.

^{13}C NMR (101 MHz, thf-d_8, 298 K) δ 149.8, 149.1, 145.5, 145.4, 141.0, 139.8, 128.4, 121.2, 106.8, 100.8, 68.3, 35.4, 35.2, 35.1, 32.4, 32.1, 28.0, 26.4, 25.9, 25.4, 25.0 ppm.

Synthesis of [Cs(^{Dipp}NON)CaF(thf)]$_2$ (5)

1-Dipp (12.8 mg, 15 µmol), Cs(HMDS) (3.9 mg, 15 µmol), and Me_3SnF (2.7 mg, 15 µmol) were added to a J. Young NMR tube. After the addition of 0.5 ml C_6D_6, the mixture was shaken and reacted over the course of 2 days at RT in which crystalline colourless blocks of **5** formed (used for single crystal XRD). No signals for the product in both ^{1}H NMR and ^{19}F NMR

were observed, signalling its insolubility in C_6D_6. After removal of the solvent, the solids were extracted with thf-d_8 to result in clean **5**.

^{1}H NMR (400 MHz, thf-d_8, 298 K) δ 7.06 (d, *J* = 7.5 Hz, 8H), 6.95 (t, *J* = 7.5 Hz, 4H), 6.13 (d, *J* = 2.2 Hz, 4H), 5.52 (d, *J* = 2.2 Hz, 4H), 3.54 (m, 8H), 3.35 (hept, *J* = 6.7 Hz, 8H), 1.68 (m, 8H), 1.53 (s, 12H), 1.05 (s, 36H), 1.03 (d, *J* = 6.8 Hz, 12H), 1.00 (d, *J* = 6.8 Hz, 12H) ppm.

^{19}F NMR (377 MHz, thf-d_8, 298 K) δ -51.75 ppm.

^{13}C NMR (151 MHz, thf-d_8, 298 K) δ 153.2, 149.6, 146.7, 145.4, 140.1, 129.3, 123.9, 122.0, 107.0, 100.8, 68.3, 35.6, 35.1, 32.4, 31.2, 27.9, 26.4, 25.8 ppm.

Synthesis of (^{Dipp}NON)CaF(thf)(K 18-crown-6) (6)

In a J. Young NMR tube, 4-Dipp (9.0 mg, 5.4 µmol) was dissolved in 0.5 ml C_6D_6, followed by the stepwise addition and dissolving of 18-crown-6 (2.9 mg, 10.8 µmol). Crystals suitable for single crystals X-ray diffraction of **6** are formed upon standing after several hours. The liquid was removed and the crystals were washed with benzene. Once formed, crystals of **6** are only sparingly soluble in benzene (note: still resulting in the same ^{19}F NMR shift as before crystallization) and slightly more soluble in thf-d_8. The formation of **6** was also observed in the direct reaction of **1-Dipp** with KF and 18-crown-6 in benzene-d_6 (identified by the fluorine signal at δ_F = -90.5 ppm), albeit in low yields (ca. 4%) and requiring prolonged heating at reflux (ca. 3 weeks).

^{1}H NMR (400 MHz, thf-d_8, 298 K) δ 6.99 (d, *J* = 7.6 Hz, 4H), 6.79 (t, *J* = 7.5 Hz, 2H), 6.10 (d, *J* = 2.2 Hz, 2H), 5.50 (d, *J* = 2.2 Hz, 2H), 3.55 (m, 4H), 3.49 (hept, *J* = 6.9 Hz, 4H), 3.28 (s, 24H), 1.68 (m, 4H), 1.55 (s, 6H), 1.12 (d, *J* = 6.9 Hz, 12H), 1.06 (s, 18H), 1.03 (d, *J* = 6.9 Hz, 12H) ppm.

^{19}F NMR (377 MHz, thf-d_8, 298 K) δ -88.94 ppm.

^{19}F NMR (377 MHz, C_6D_6, 298 K) δ -90.53 ppm.

^{13}C NMR (101 MHz, thf-d_8, 298 K) δ 152.4, 150.2, 146.1, 145.4, 139.9, 129.0, 123.4, 121.5, 107.1, 100.5, 71.0, 68.3, 35.5, 35.1, 32.4, 31.7, 28.0, 26.4, 25.9, 25.5 ppm.

Elemental analysis: anal. calc. for $C_{63}H_{94}CaFKN_2O_8$: C 68.44%, H 8.57%, N 2.53%, found: C 68.84%, H 8.46%, N 2.32%.

Synthesis of (^{Dipp}NON)CaF(thf)(K benzo-18-crown-6) (7)

In a J. Young NMR tube, 4-Dipp (9.0 mg, 5.4 µmol) was dissolved in 0.5 ml C_6D_6, followed by the stepwise addition and dissolving of benzo-18-crown-6 (3.4 mg, 10.8 µmol). Crystals suitable for single crystals X-ray diffraction of **7** are formed upon standing after several hours. The liquid was removed and the crystals were washed with benzene. Once formed, crystals of **7** are only sparingly soluble in benzene (note: still resulting in the same ^{19}F NMR shift as before crystallization) and slightly more soluble in thf-d_8.

^{1}H NMR (500 MHz, thf-d_8, 298 K) δ 6.95 (d, *J* = 7.6 Hz, 4H), 6.87 – 6.79 (m, 2H), 6.83 (d, *J* = 3.1 Hz, 2H), 6.79 (t, *J* = 7.6 Hz, 2H), 6.07 (d, *J* = 2.3 Hz, 2H), 5.46 (d, *J* = 2.2 Hz, 2H), 3.92 – 3.88 (m, 4H), 3.55 – 3.52 (m, 4H), 3.47 (hept, *J* = 6.9 Hz, 4H), 3.46 – 3.40 (m, 4H), 3.39 – 3.36 (m, 4H), 3.36 – 3.34 (m, 4H), 3.34 (s, 4H), 1.70 – 1.67 (m, 4H), 1.52 (s, 6H), 1.05 (d, *J* = 6.9 Hz, 12H), 1.02 (s, 18H), 0.98 (d, *J* = 6.8 Hz, 12H) ppm.

^{19}F NMR (377 MHz, thf-d_8, 298 K) δ -88.15 ppm.

^{19}F NMR (377 MHz, C_6D_6, 298 K) δ -88.81 ppm.

^{13}C NMR (126 MHz, thf-d_8, 298 K) δ 152.4, 150.1, 148.5, 146.2, 145.4, 140.0, 129.2, 123.5, 121.8, 121.5, 112.3, 107.2, 100.5, 71.1, 70.8, 70.7, 69.6, 68.3, 68.2, 35.4, 35.1, 32.4, 31.5, 28.1, 26.4, 25.9, 25.4 ppm.

Synthesis of [(K dibenzo-18-crown-6)K(^{Dipp}NON)CaF$_2$]$_2$ (8)

In a J. Young NMR tube, **4-Dipp** (4.5 mg, 2.7 µmol) was dissolved in 0.5 ml C_6D_6 (5.4 µmol/ml), followed by the stepwise addition and dissolving of dibenzo-18-crown-6 (2.0 mg, 5.4 µmol). When more concentrated solutions (e.g. 10 µmol/ml) are used, crystals of **8'** are formed upon standing after several hours and were suitable for X-ray diffraction. The title compound (**8**) undergoes a structural change upon crystallization forming **8'** which is insoluble in benzene. Consequently, only NMR spectroscopic data of **8** could be acquired (on a dilute, non-isolated reaction mixture to prevent crystallization).

^{1}H NMR (500 MHz, C_6D_6, 298 K) δ 7.21 (d, J = 7.6 Hz, 4H), 7.03 (t, J = 7.5 Hz, 2H), 6.63 (br s, 4H), 6.60 (d, J = 2.3 Hz, 2H), 6.41 (br s, 4H), 6.08 (d, J = 2.2 Hz, 2H), 3.84 (hept, J = 6.9 Hz, 4H), 3.69 (m, 4H), 3.43 (br s, 8H), 3.17 (br s, 8H), 1.88 (s, 6H), 1.42 (s, 18H), 1.32 (d, J = 7.0 Hz, 12H), 1.31 – 1.27 (m, 4H), 1.15 (d, J = 7.0 Hz, 12H) ppm.

^{19}F NMR (470 MHz, C_6D_6, 298 K) δ 89.29 ppm.

^{13}C NMR (101 MHz, C_6D_6, 298 K) δ 152.4, 149.6, 147.3, 146.2, 145.8, 139.0, 128.6, 123.2, 121.3, 113.8, 110.9, 107.2, 102.1, 68.7, 66.8, 64.4, 35.2, 35.0, 32.3, 32.3, 27.8, 25.6, 25.4, 25.3 ppm.

Supplementary data

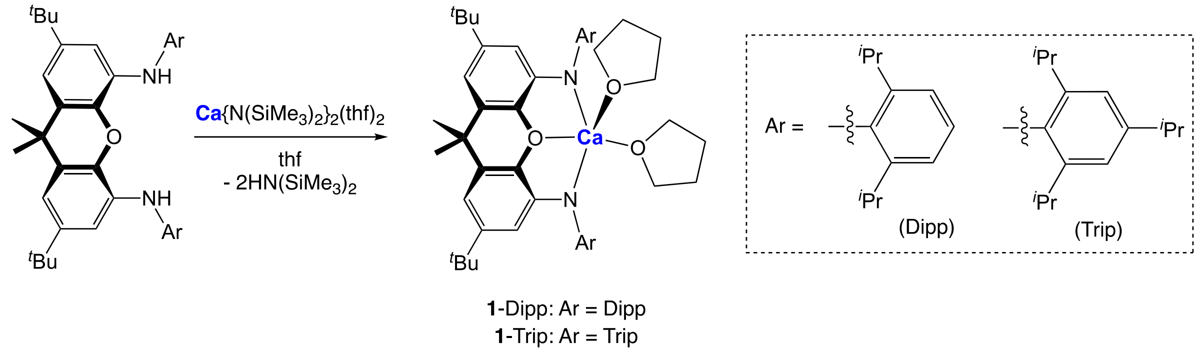

***Figure S 1.** Syntheses of (^{Dipp}NON)Ca(thf)$_2$ (**1-Dipp**) and (^{Trip}NON)Ca(thf)$_2$ (**1-Trip**) via the reactions of Ca(HMDS)$_2$(thf)$_2$ with the respective protio ligands (NON)H$_2$.*

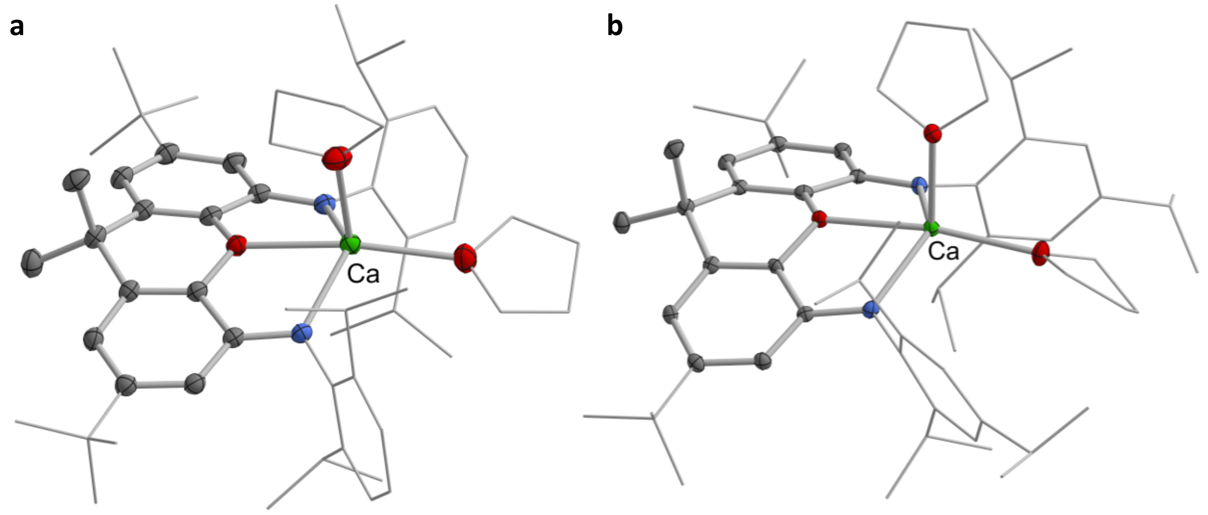

***Figure S 2**. Molecular structures of (^{Dipp}NON)Ca(thf)$_2$ (**1**-Dipp, a) and of one of the two independent molecules in the asymmetric unit of (^{Trip}NON)Ca(thf)$_2$ (**1**-Trip, b) in the solid state, as determined by X-ray crystallography. Thermal ellipsoids set at the 30% level. Hydrogen atoms, solvate molecule and second disorder component omitted, and selected fragments shown in wireframe format for clarity.*

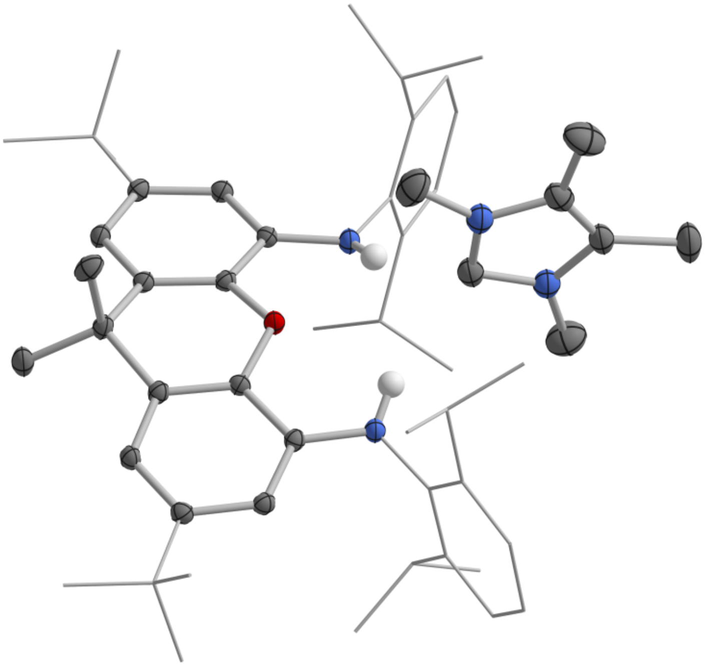

***Figure S 3**. Molecular structure of (^{Dipp}NON)H_2·IMe_4 in the solid state, as determined by X-ray crystallography. Thermal ellipsoids set at the 30% level. Most hydrogen atoms omitted and selected fragments shown in wireframe format for clarity. CCDC reference: 2283072.*

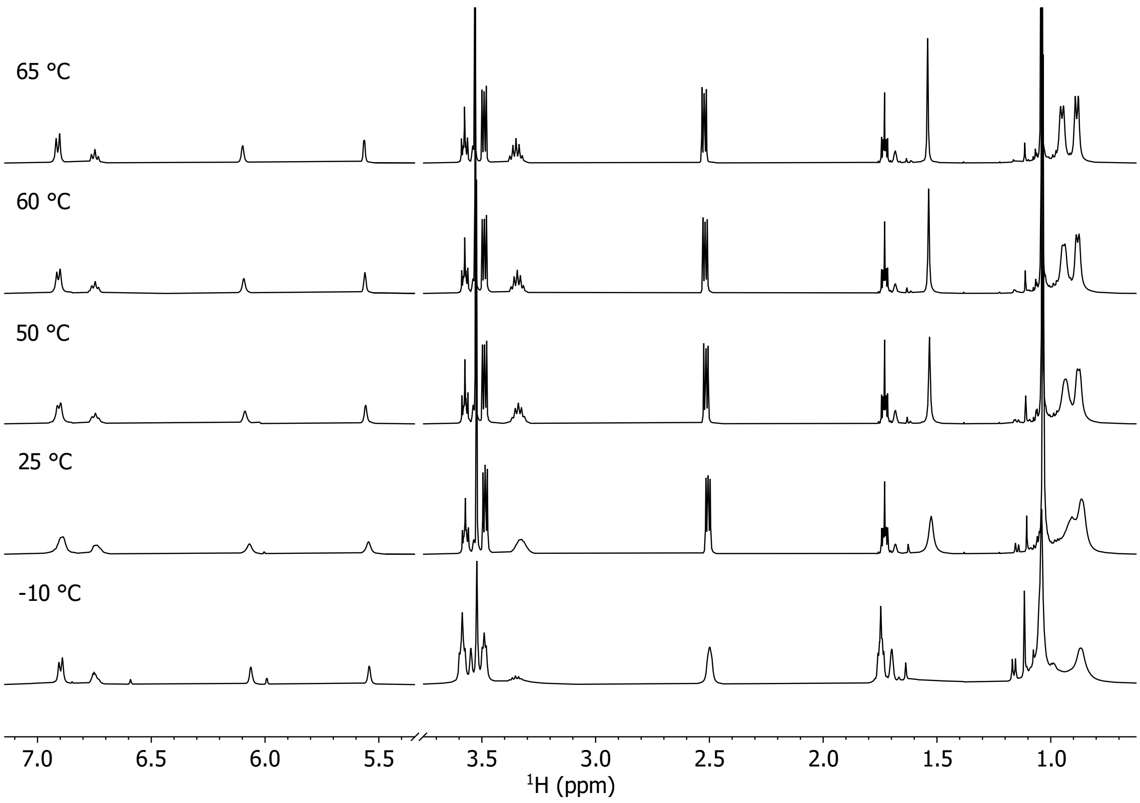

Figure S 4. *Variable temperature (-10 - 65 °C) ¹H NMR spectrum of **3** in thf-d_8. The rotational barrier was determined to be 55 ± 1 kJ/mol (calculated based on the coalescence of the CH_3 groups at 0.80-1.00 ppm).*

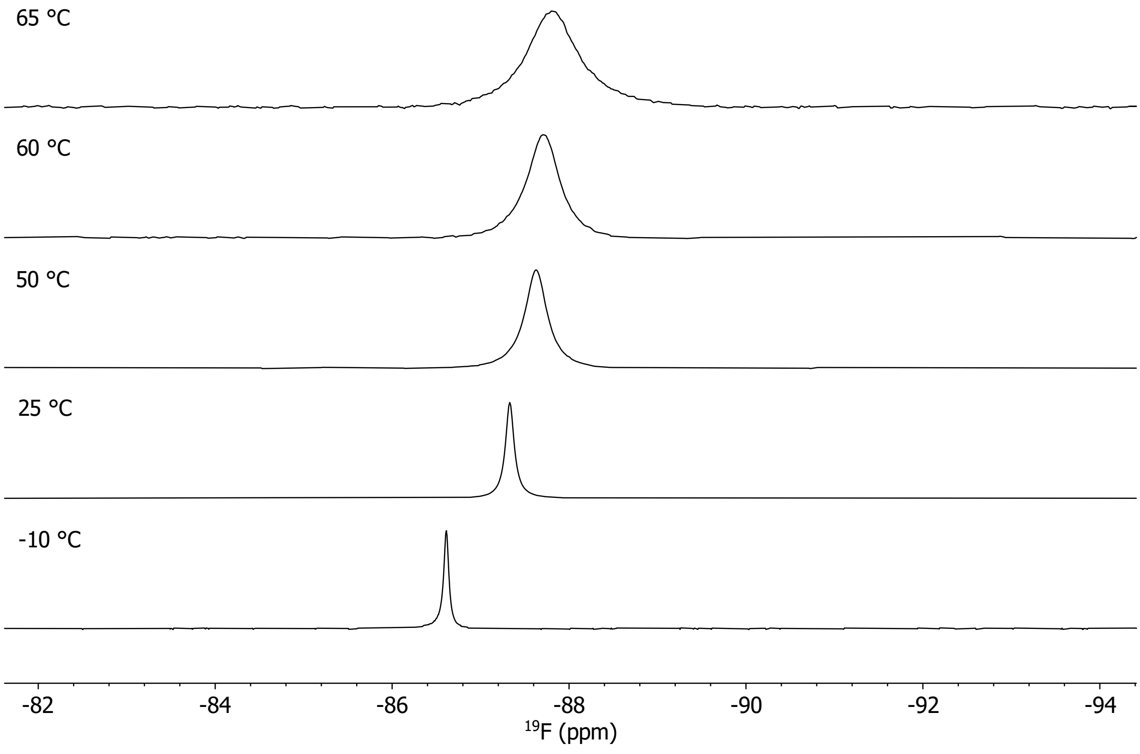

Figure S 5. *Variable temperature (-10 - 65 °C) ¹⁹F NMR spectrum of **3** in thf-d_8.*

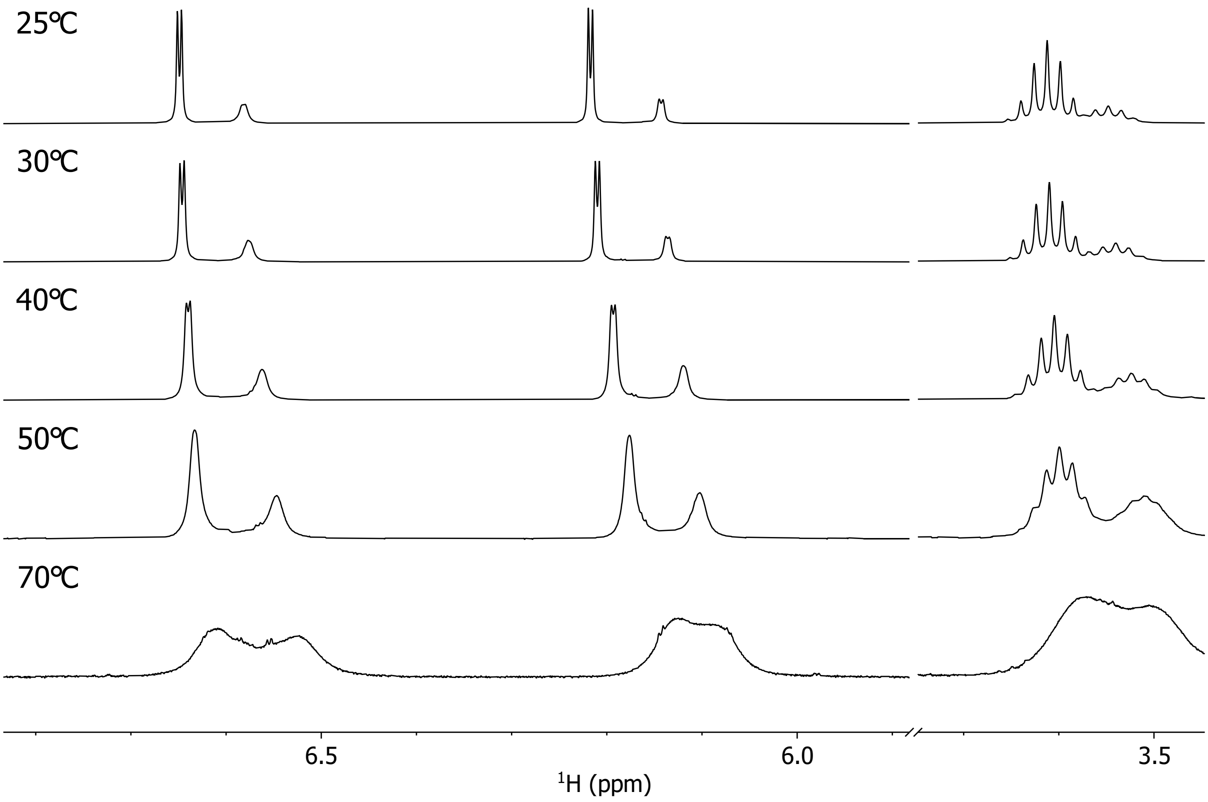

Figure S 6. *Variable temperature (25 - 70 °C)* ^{1}*H NMR spectrum of* ***4-Dipp*** *with K(HMDS) (1 equiv.) in* C_6D_6.

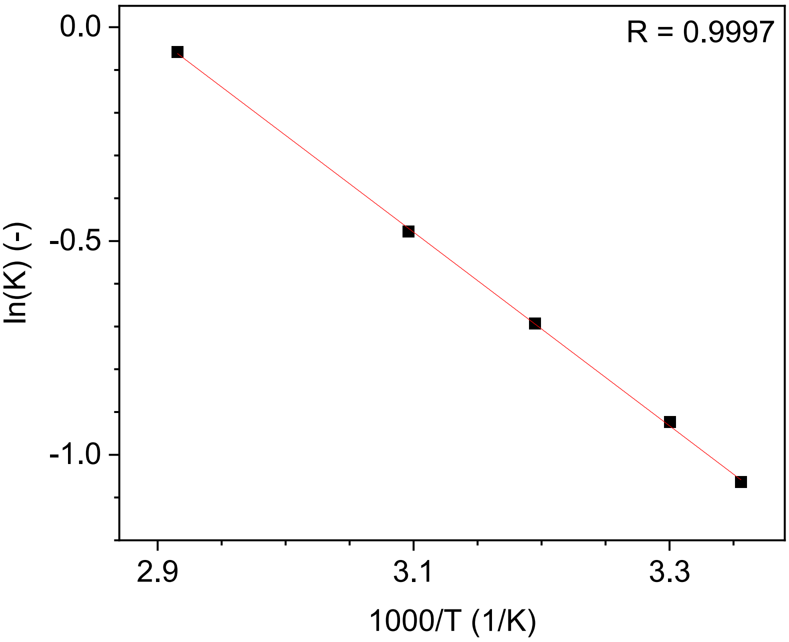

Figure S 7. *VT NMR and van't Hoff plot for HMDS complexation with* ***1****-Dipp derived from integrated* ^{1}*H NMR signal intensities.*

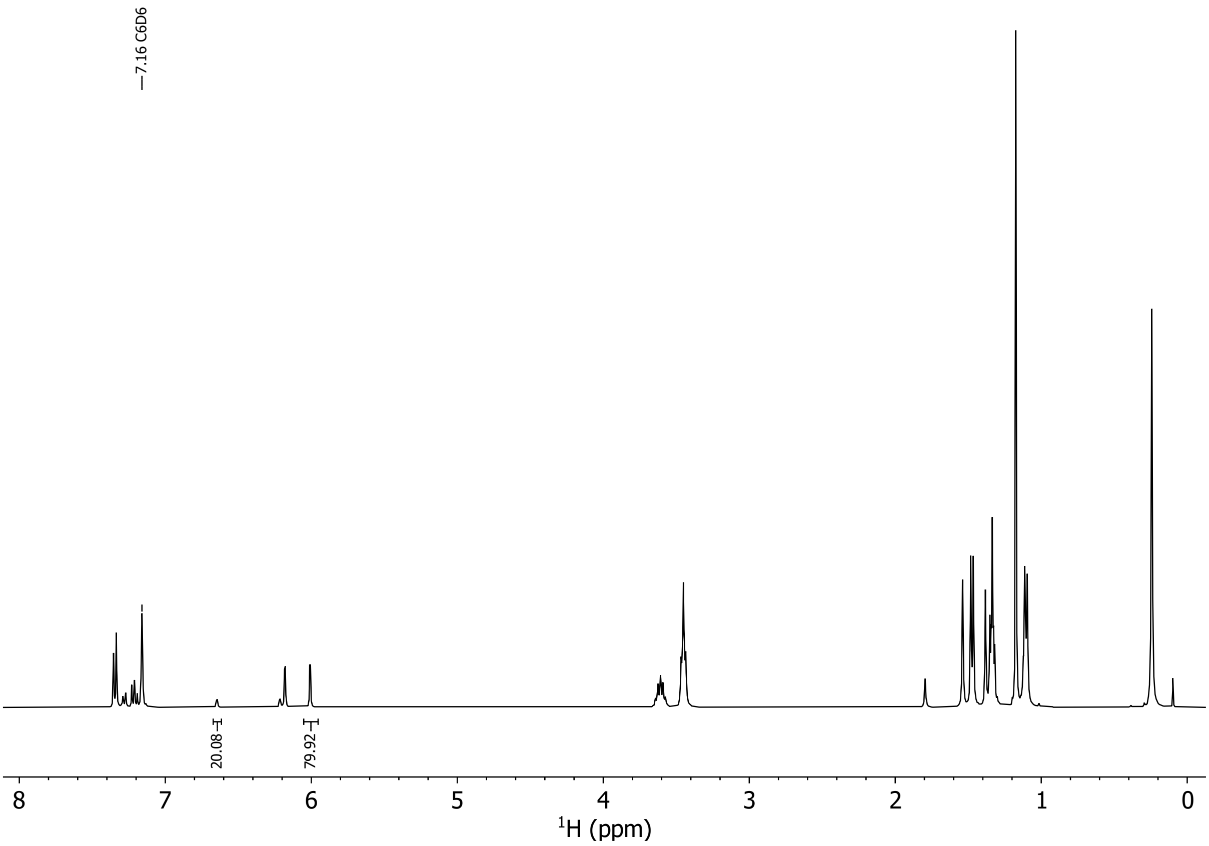

Figure S 8. *^{1}H NMR spectrum of **4-Dipp** with Cs(HMDS) (1 equiv.) in C_6D_6.*

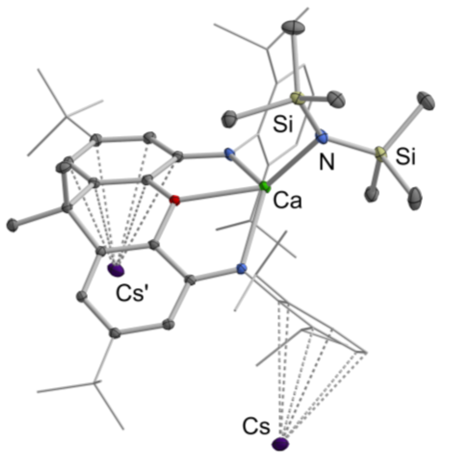

Figure S 9. *Molecular structure of Cs[(^{Dipp}NON)Ca(HMDS)] prepared from **1**-Dipp and Cs(HMDS) in the solid state as determined by X-ray crystallography. Thermal ellipsoids set at the 30% level. Hydrogen atoms and solvate molecules omitted, and selected fragments shown in wireframe format for clarity.*

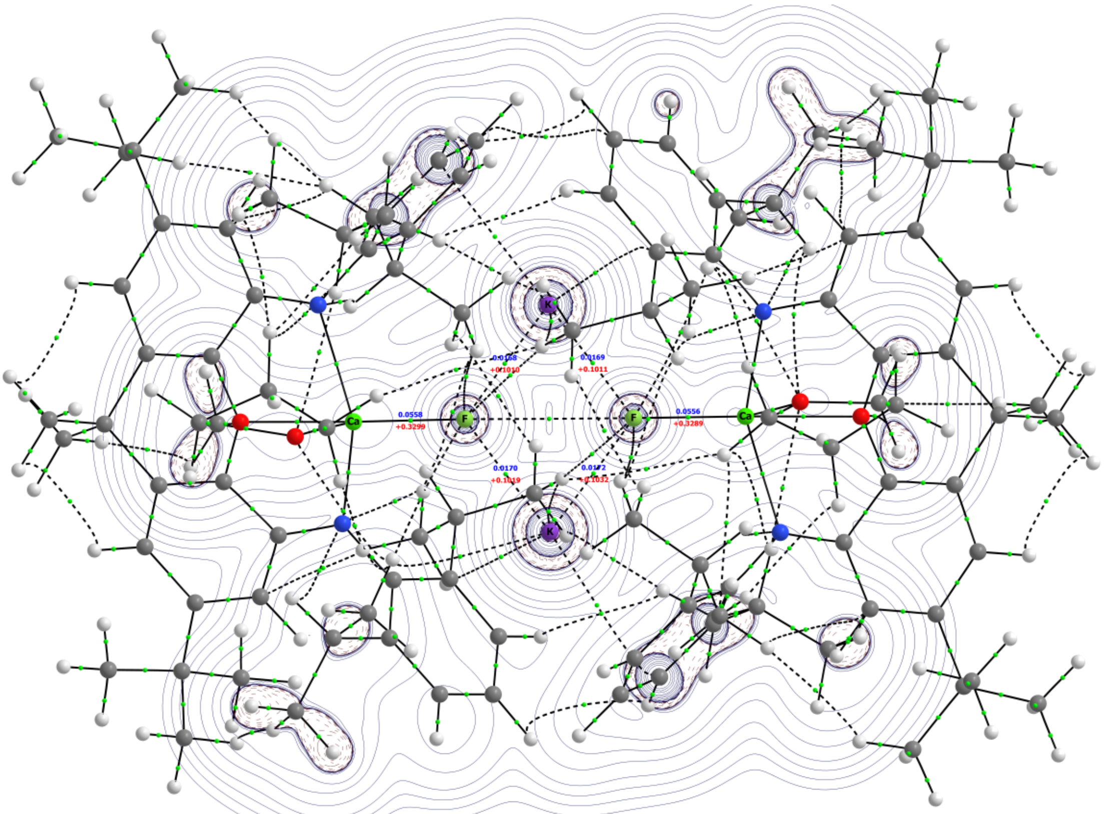

Figure S 10 *QTAIM analysis of* ***4****-Dipp. Bond critical points (BCP) shown in green were located between Ca-F and K-F, although the Rho values (in blue and its derivative in red) are low as befitting the predominantly ionic nature of the interactions. However, the value found for the Ca-F BCP (0.056 e/bohr^3) is significantly higher than its K-F counterpart (0.017 e/bohr^3).*

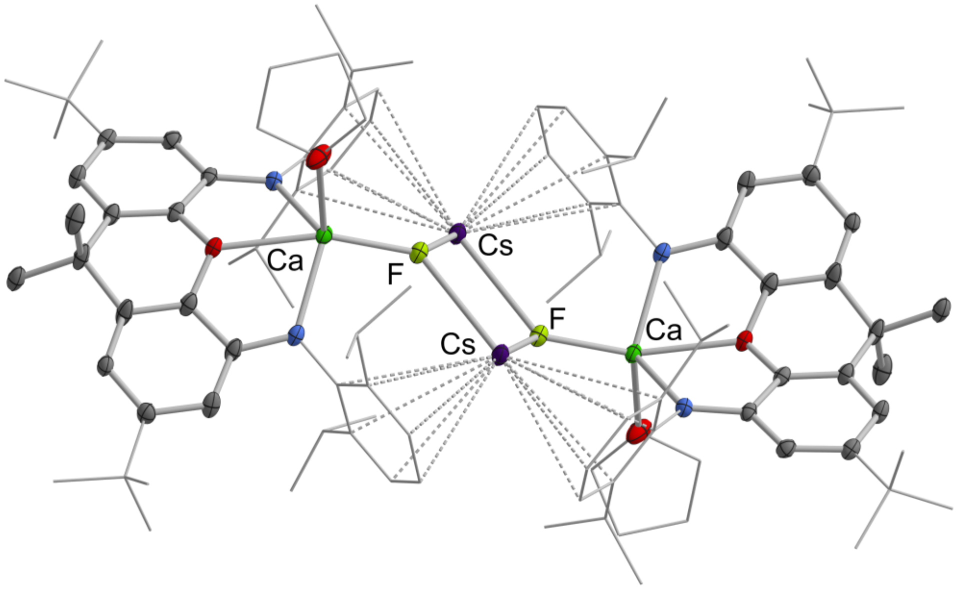

Figure S 11*. Centrosymmetric molecular structure of [Cs(^{Dipp}NON)Ca(thf)F]$_2$ (**5**) in the solid state, as determined by X-ray crystallography. Thermal ellipsoids set at the 30% level. Hydrogen atoms, second disorder component and solvate molecule omitted, and selected fragments shown in wireframe format for clarity. Selected bond lengths (Å): Ca–F 2.137(2), Cs···F 2.845(3), 2.908(2).*

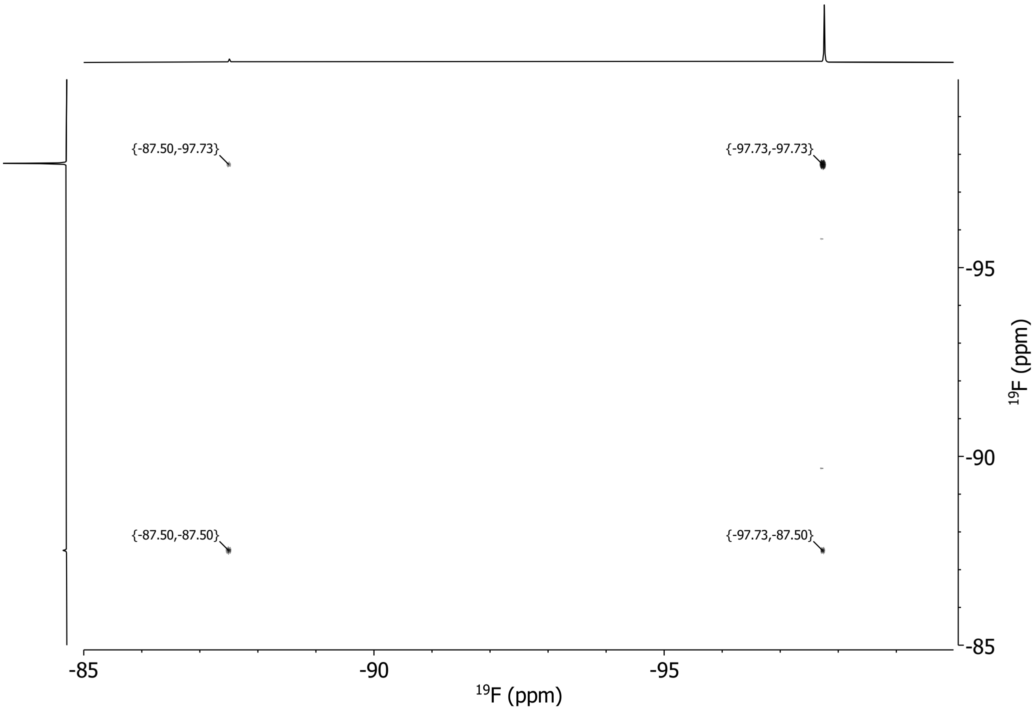

Figure S 12. *^{19}F-^{19}F EXSY NMR spectrum of **4-Dipp** (major) and **4'-Dipp** (minor) in C_6D_6.*

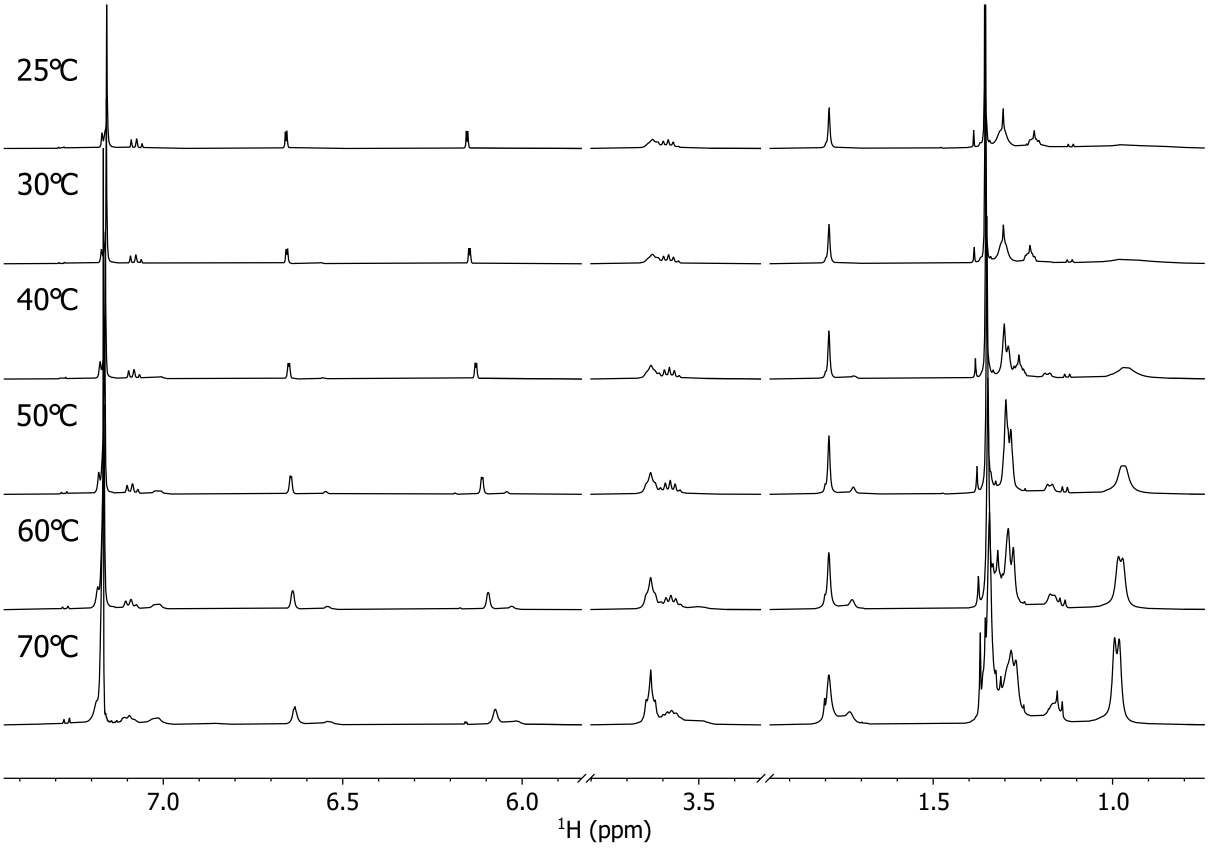

Figure S 13. *Variable temperature (25 - 70 °C) ^{1}H NMR spectrum of **4-Dipp** (major) and **4'-Dipp** (minor) in C_6D_6.*

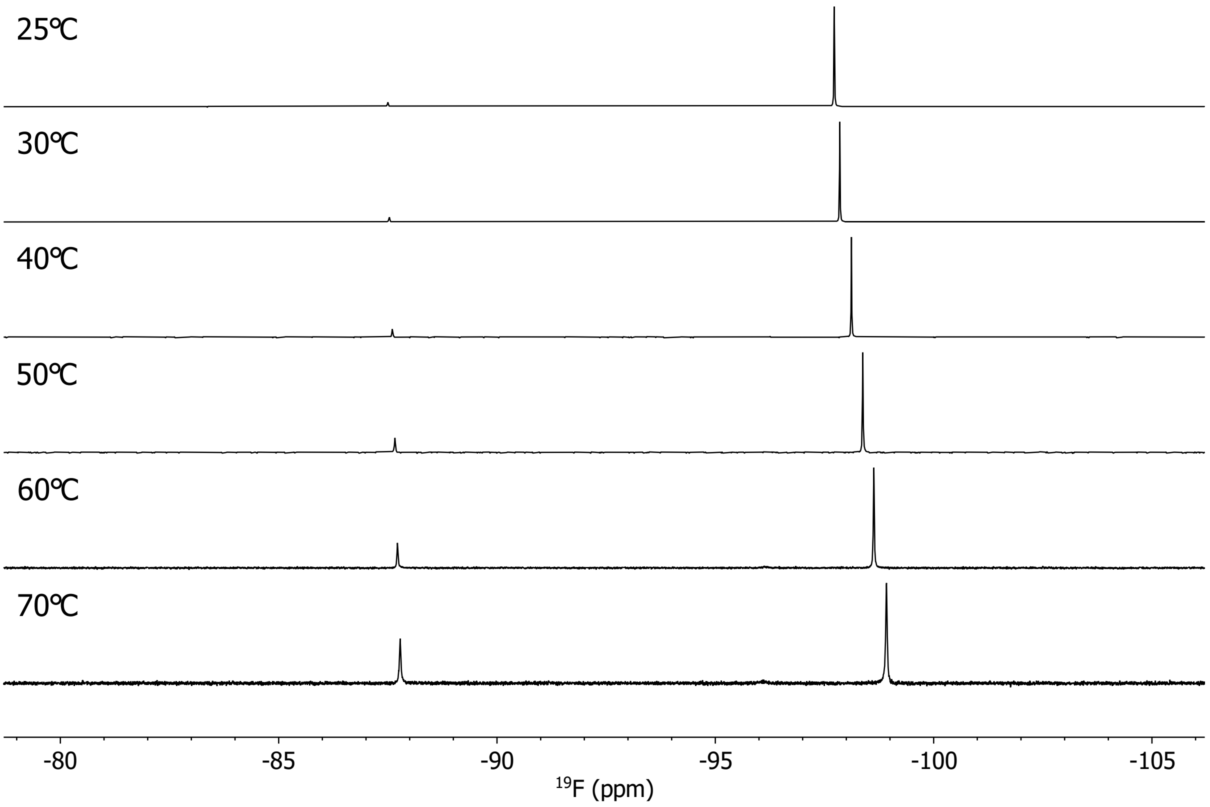

Figure S 14*. Variable temperature (25 - 70 °C) ^{19}F NMR spectrum of* ***4-Dipp*** *(major) and* ***4'-Dipp*** *(minor) in C_6D_6.*

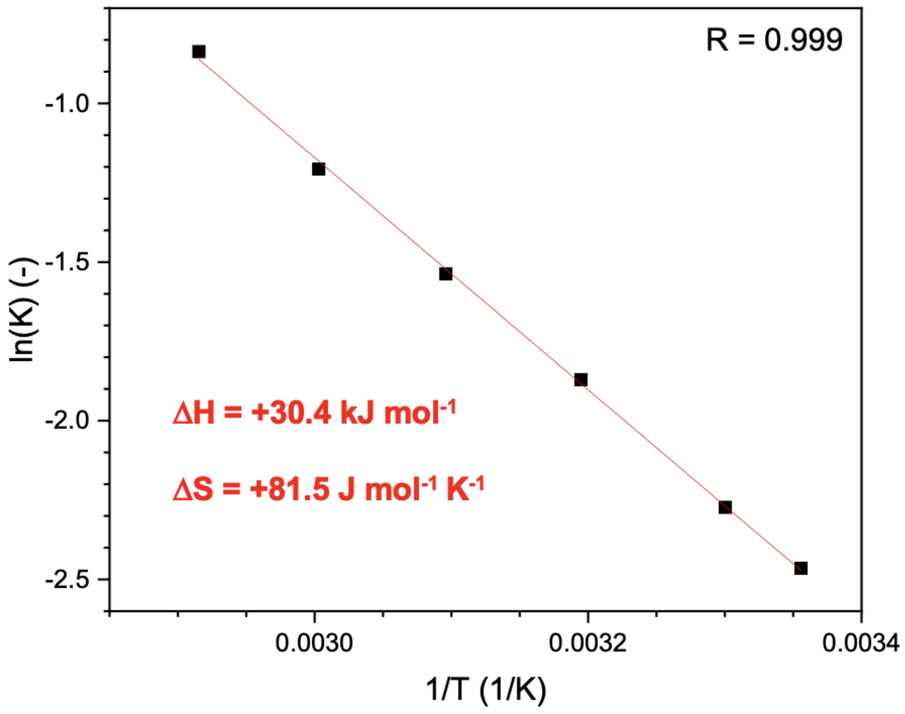

Figure S 15*. Van't Hoff plot for interconversion between* ***4****-Dipp and* ***4'****-Dipp derived from integrated ^{19}F NMR signal intensities.*

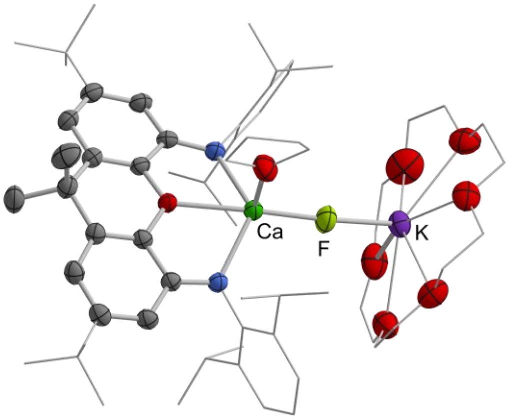

Figure S 16. *Molecular structure of [K(18-crown-6)FCa(thf)(*[Dipp]*NON)] (**6**) in the solid state, as determined by X-ray crystallography. Thermal ellipsoids set at the 30% level. Hydrogen atoms, solvate molecules and second disorder component omitted, and selected fragments shown in wireframe format for clarity. Selected bond lengths (Å) and angles (°): Ca–F 2.085(8), K–F 2.472(9), Ca–F–K 160.9(4).*

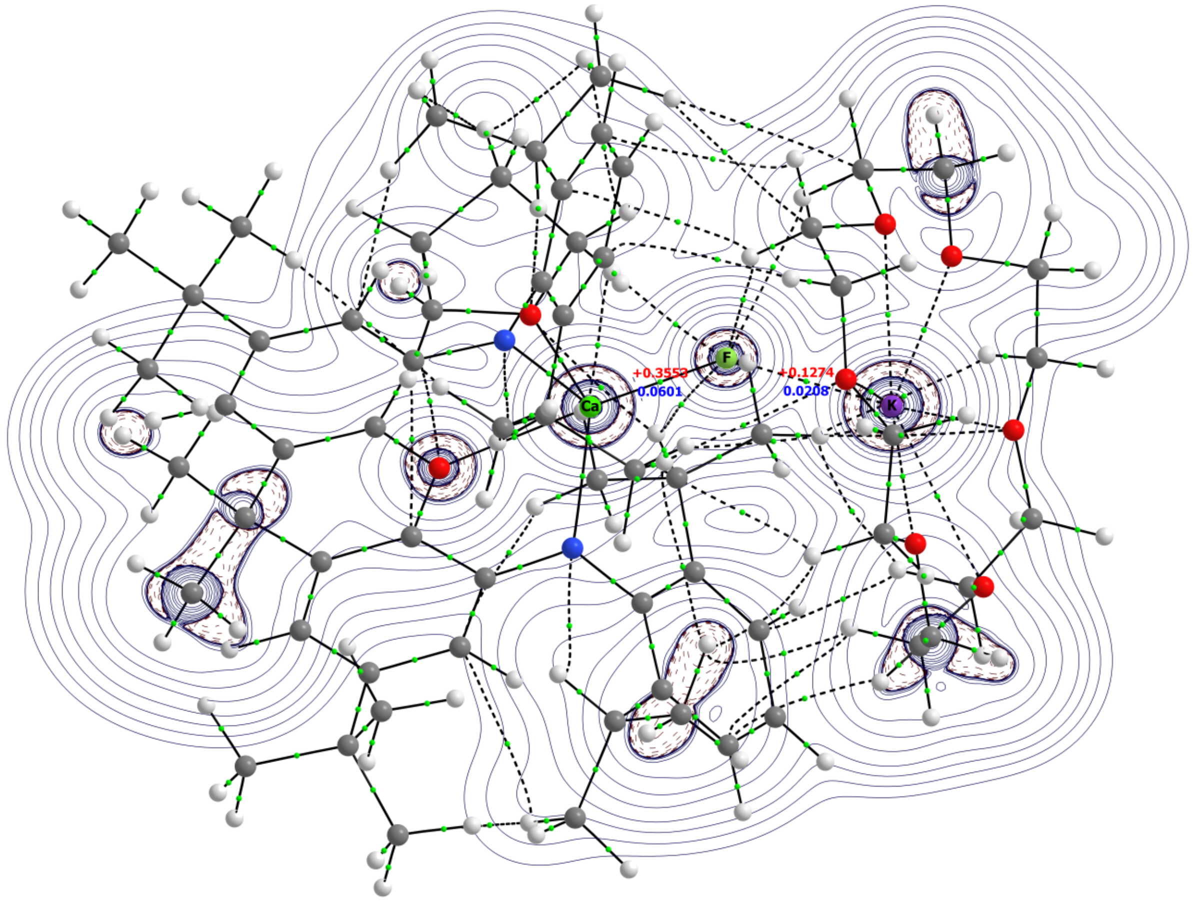

***Figure S 17** QTAIM analysis of **6**. Bond critical points shown in green were located between Ca-F and K-F, although the Rho values (in blue and its derivative in red) are low as befitting the predominantly ionic nature of the interactions. However, the value found for the Ca-F BCP (0.060 e/bohr^3) is significantly higher than its K-F counterpart (0.021 e/bohr^3).*

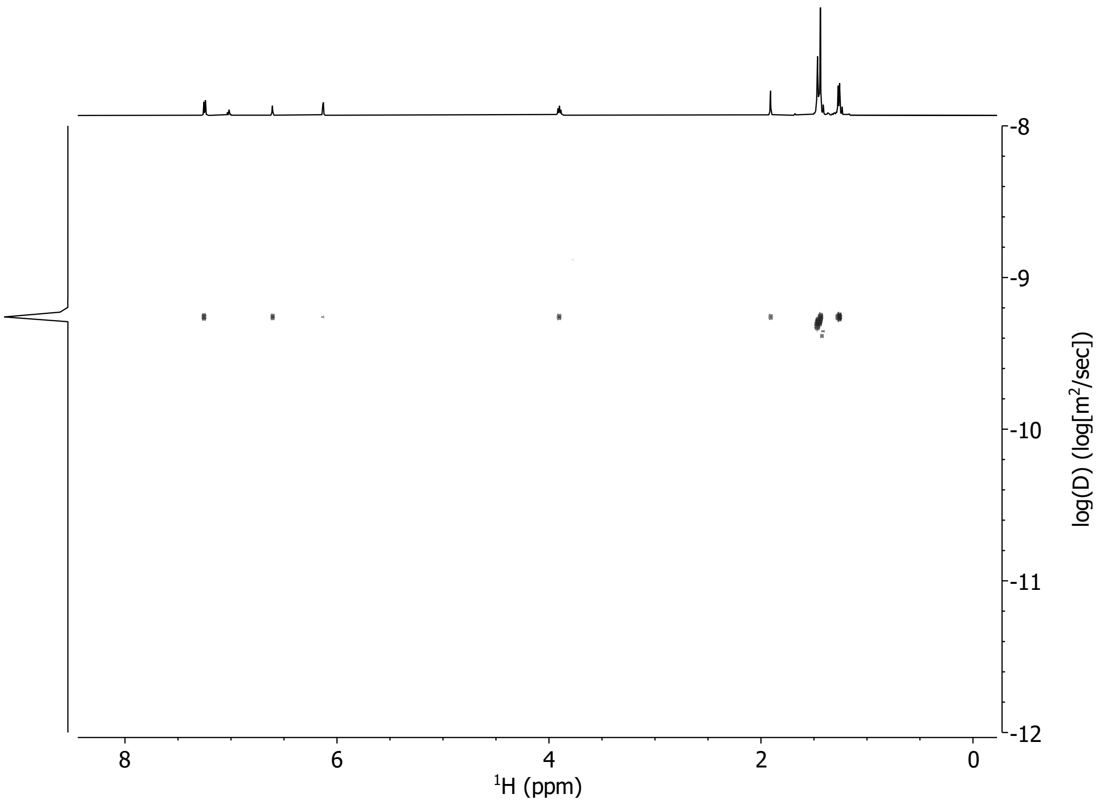

Figure S 18. *^{1}H DOSY NMR of an in-situ sample of **6**. The diffusion constant aligns with the species being present in solution as in solid-state ($M_{w,DOSY}$ ≈ 1000 g/mol, $M_{w,act}$ = 1106 g/mol).*

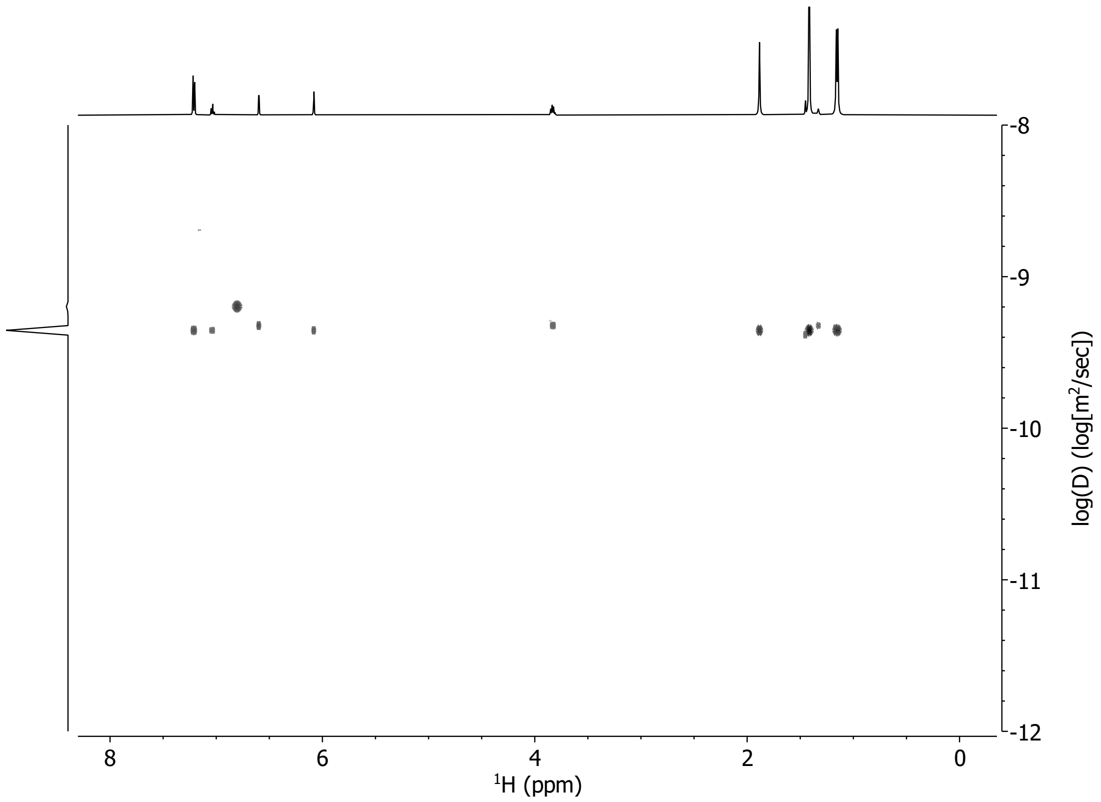

Figure S 19. *^{1}H DOSY NMR of an in-situ sample of **8**. The diffusion constant aligns with the species being present in solution as a monomer analogues to **6** and **7** ($M_{w,DOSY}$ ≈ 1300 g/mol, $M_{w,act}$ = 1202 g/mol) in contrast to the solid state structure **8’** ($M_{w,act}$ = 2520 g/mol).*

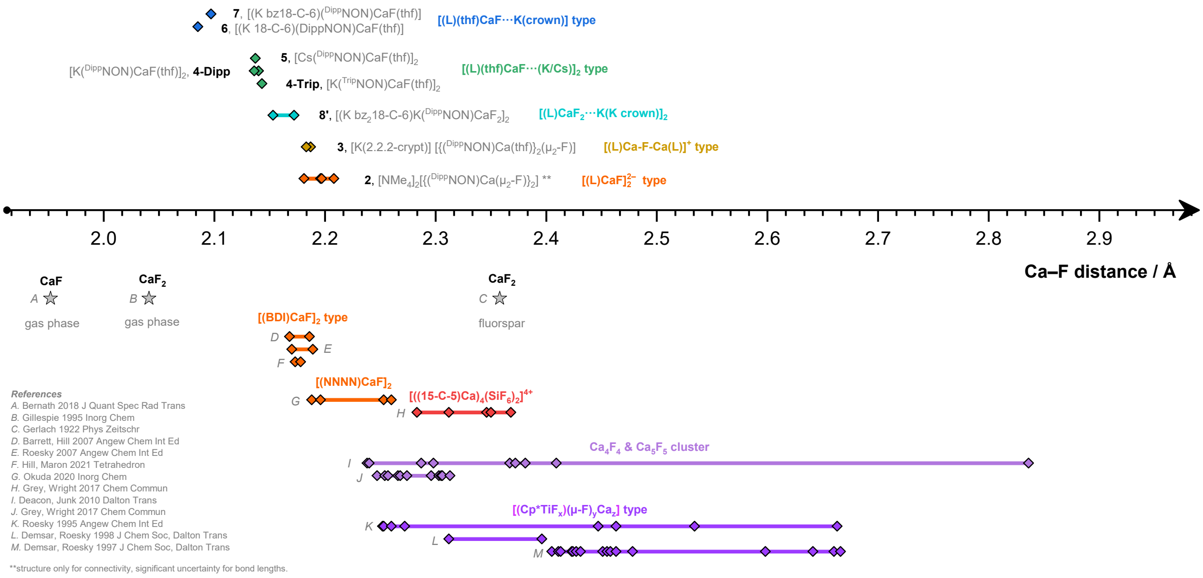

***Figure S 20**. Overview of relevant Ca-F bond lengths in literature (below axis) and this work (above axis)[20–29].*

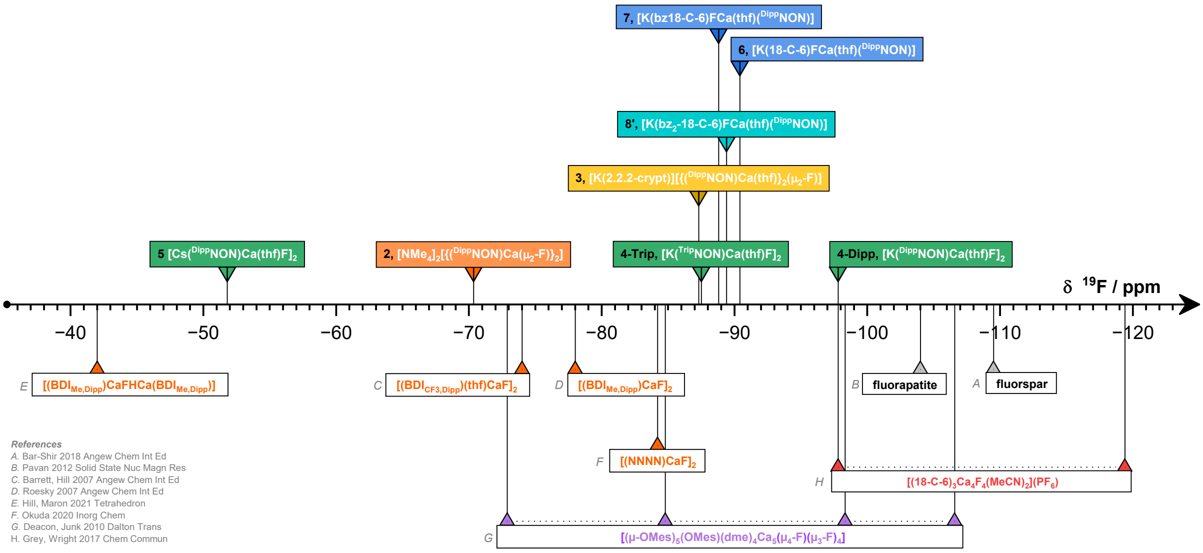

***Figure S 21**. Overview of relevant ^{19}F NMR shifts in literature (below axis) and this work (above axis)[21–26,30,31].*

Reactivity studies

Defluorination of fluorobenzene

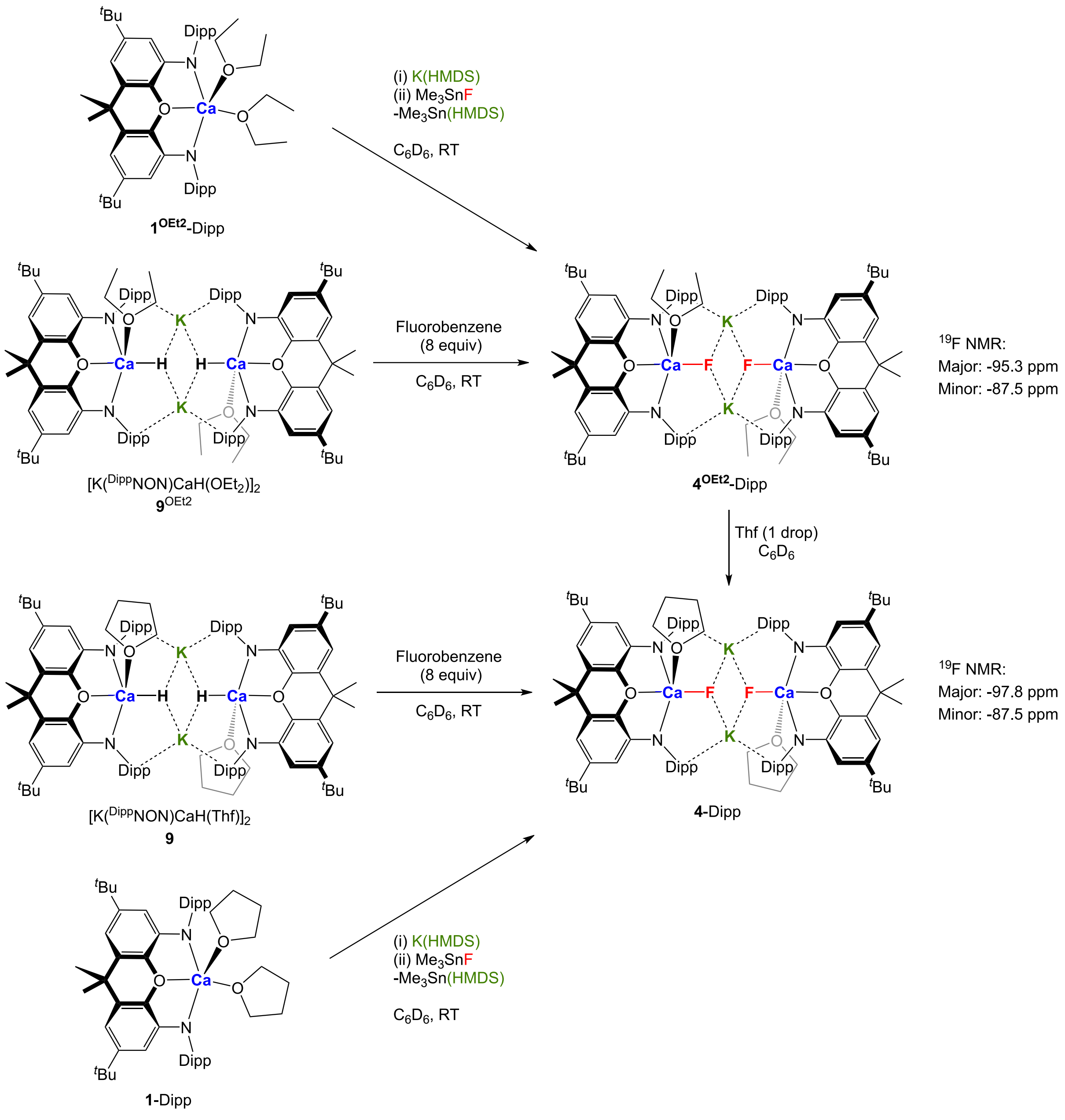

***Figure S 22**. Overview of reactions performed towards the formation of **4**-Dipp from fluorobenzene and Me_3SnF. All reactions were performed in J-Young NMR tubes. Reactions with fluorobenzene: to a 0.5 ml C_6D_6 solution of the respective hydride (**9**^{OEt2} and **9**, 0.0061 mmol) was added fluorobenzene (8 equiv.) and let to react at room temperature resulting in the formation of **4**^{OEt2}-Dipp and **4**-Dipp. Both **4**^{OEt2}-Dipp and **4**-Dipp were also synthesized independently from **1**^{OEt2}-Dipp or **1**-Dipp (0.015 mmol), respectively, by reaction with K(HMDS) (0.015 mmol) and Me_3SnF (0.015 mmol) in C_6D_6. (0.5 ml) at room temperature. To all reaction mixtures containing **4**^{OEt2}-Dipp was added thf (1 drop) and let to react at room temperature resulting in the complete conversion to **4**-Dipp. The crystal structure of **9** and example NMRs can be found in the figures below.*

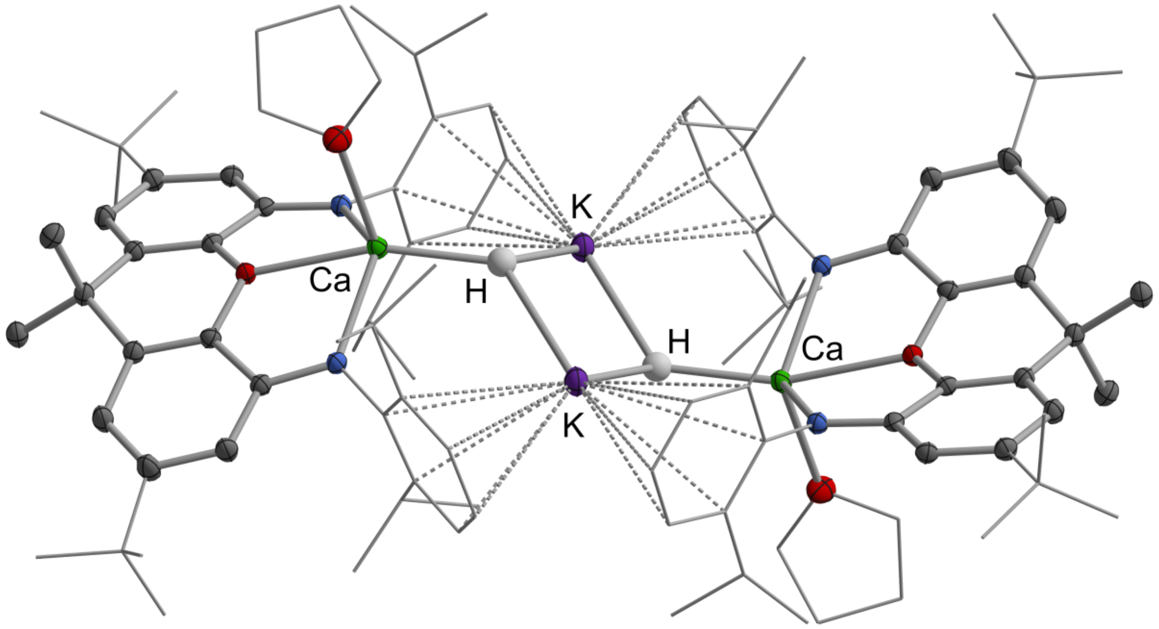

Figure S 23. *Molecular structure of [K(^{Dipp}NON)CaH(thf)]$_2$ (**9**) in the solid state, as determined by X-ray crystallography. Thermal ellipsoids set at the 30% level. Selected hydrogen atoms, solvate molecules and second disorder component omitted, and selected fragments shown in wireframe format for clarity.*

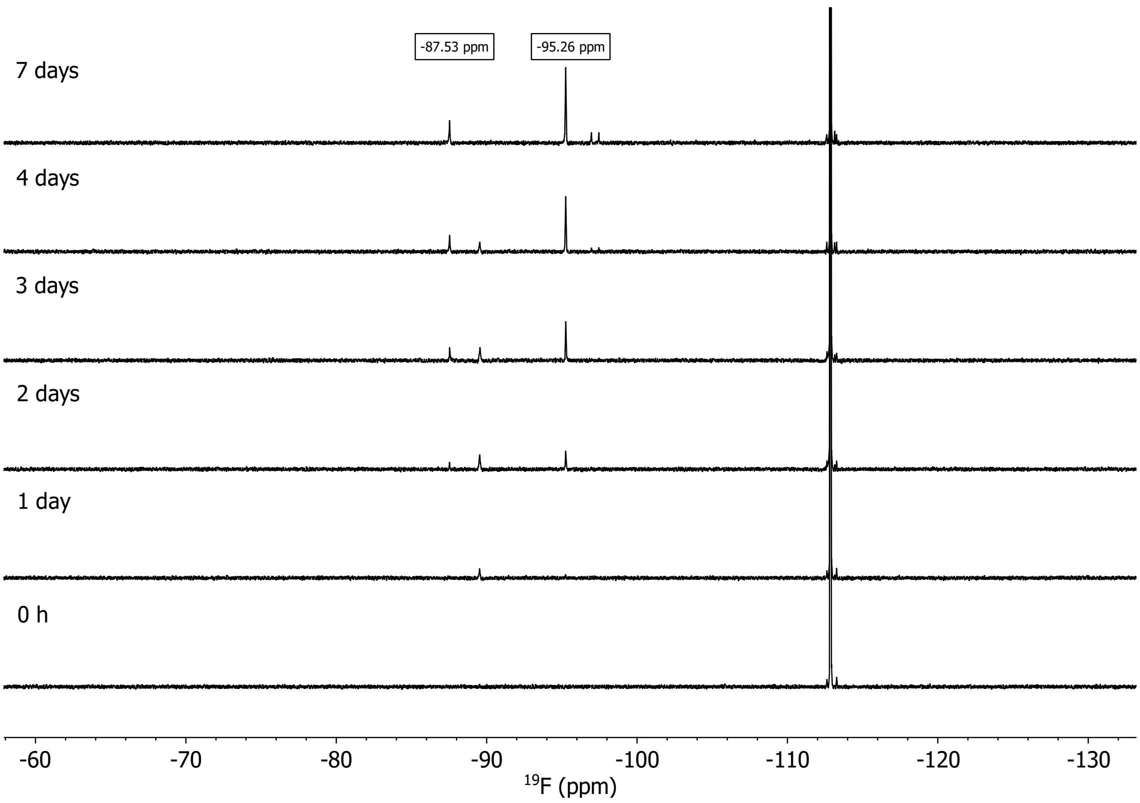

Figure S 24 *Example reaction of fluorobenzene (-112.9 ppm) with [K(^{Dipp}NON)CaH(OEt$_2$)]$_2$ in C_6D_6 to form* ***4***^{OEt2}*-Dipp. Analogous to* ***4****-Dipp,* ***4***^{OEt2}*-Dipp gives rise to two conformers: a major species at -95.3 ppm and a minor species at -87.5 ppm.*

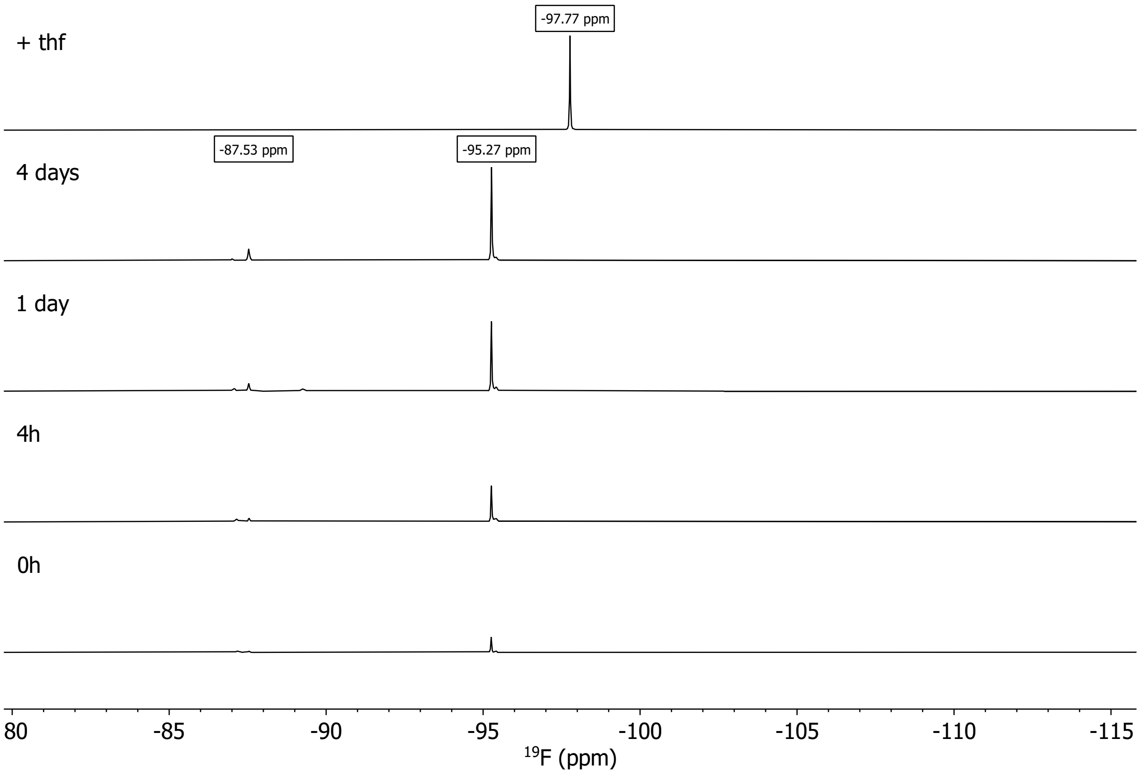

Figure S 25 *Example of independent synthesis of* ***4***^{OEt2}*-Dipp with K(HMDS) and Me_3SnF in C_6D_6 and subsequent reaction towards* ***4****-Dipp by addition of thf.*

Nucleophilic fluoride transfer

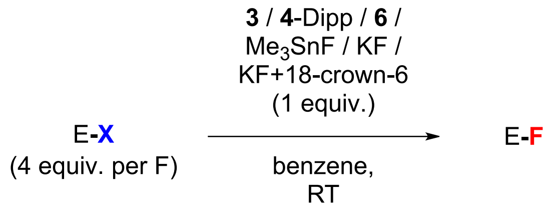

Figure S 26 *Reaction scheme for fluoride transfer from several different fluoride sources (i.e., **4**-Dipp, **3**, **6** and, for control reactions, Me_3SnF, KF and KF + 18-crown-6; all 0.005 mmol fluoride, 1 equiv.) to C(sp^2), C(sp^3), S, Si, and P based electrophiles (i.e., AdCOCl, 1-bromopentane, TsCl, Ph_3SiCl, PPh_3Cl_2; 4 equiv.). All reactions were performed in a J-Young NMR tube at room temperature (except for selected reactions with 1-bromopentane at 80 °C where indicated). All products matched characterization reported in literature[32–36]. All yields were determined by quantitative ^{19}F NMR spectroscopy with PhF (5 µL) as internal standard (the integrated standard was set to 100 and the yield subsequently calculated from the product integral). Selected examples are given below.*

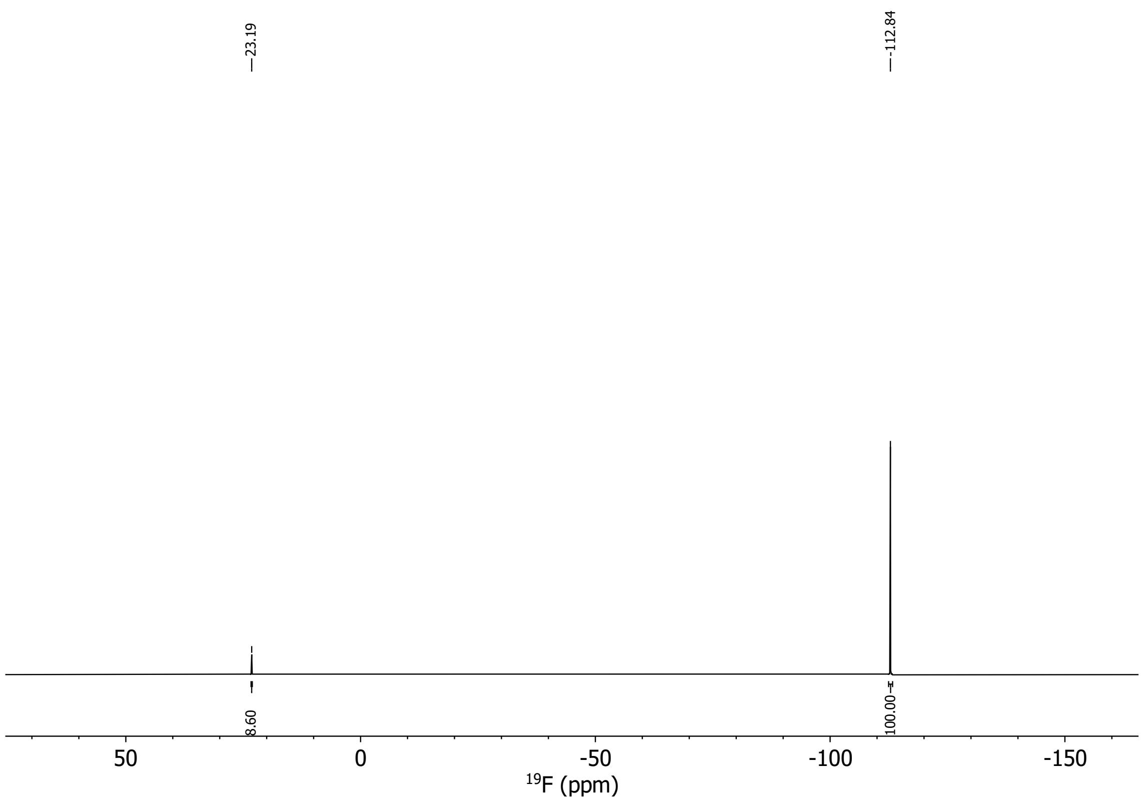

Figure S 27 *Quantitative ^{19}F NMR spectrum for the reaction of **4**-Dipp with AdCOCl in C_6D_6 after 24 h at RT.*

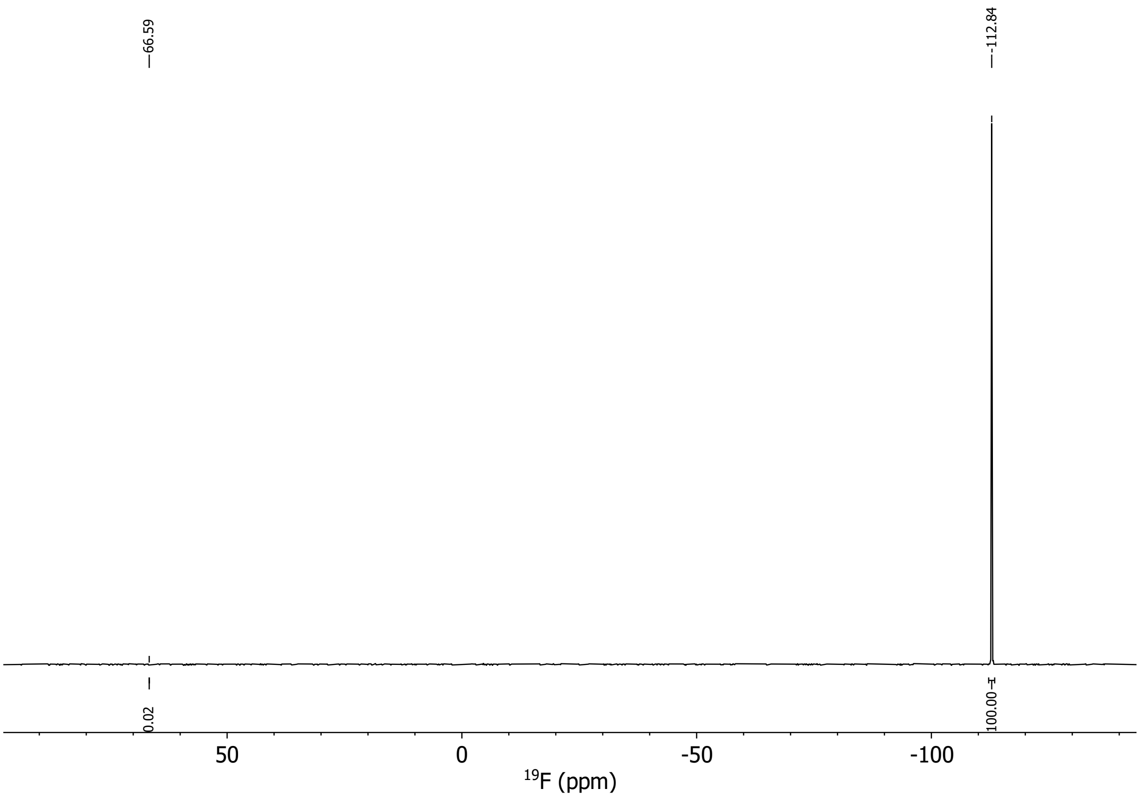

Figure S 28 *Quantitative* ^{19}*F NMR spectrum for the reaction of* ***4****-Dipp with TsCl in* C_6D_6 *after 24 h at RT.*

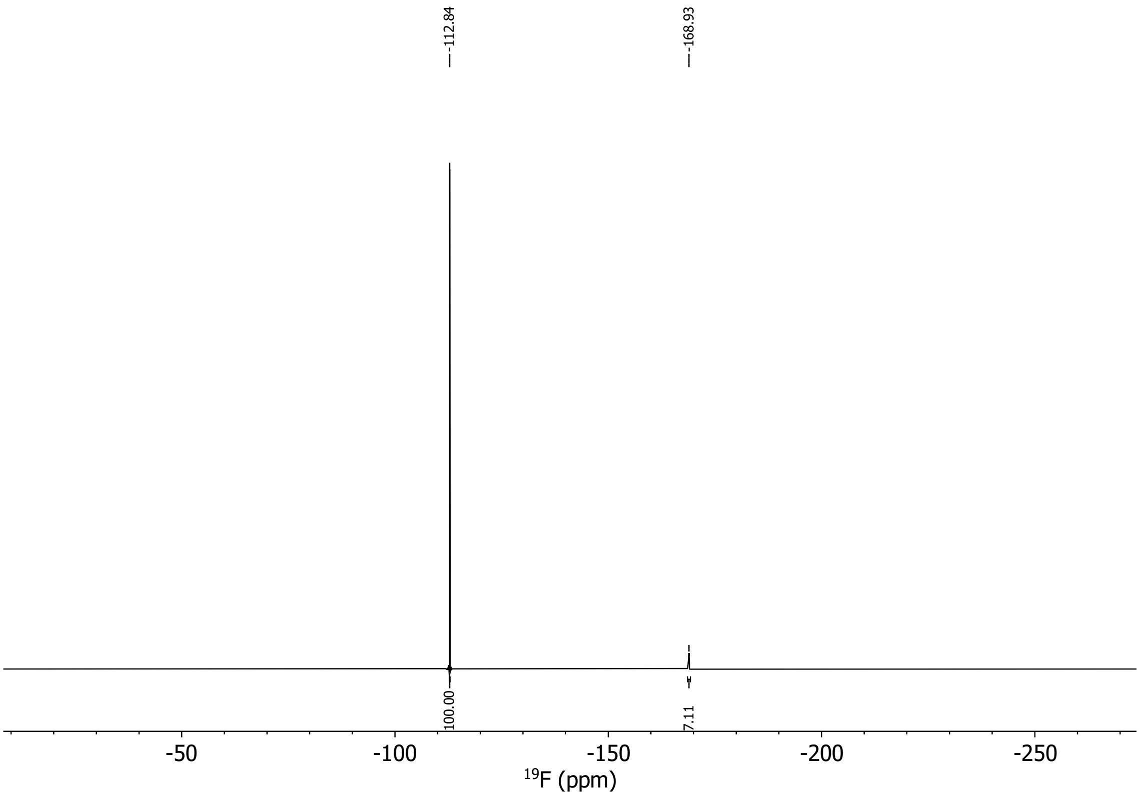

Figure S 29 *Quantitative* ^{19}*F NMR spectrum for the reaction of* ***4****-Dipp with* Ph_3SiCl *in* C_6D_6 *after 24 h at RT.*

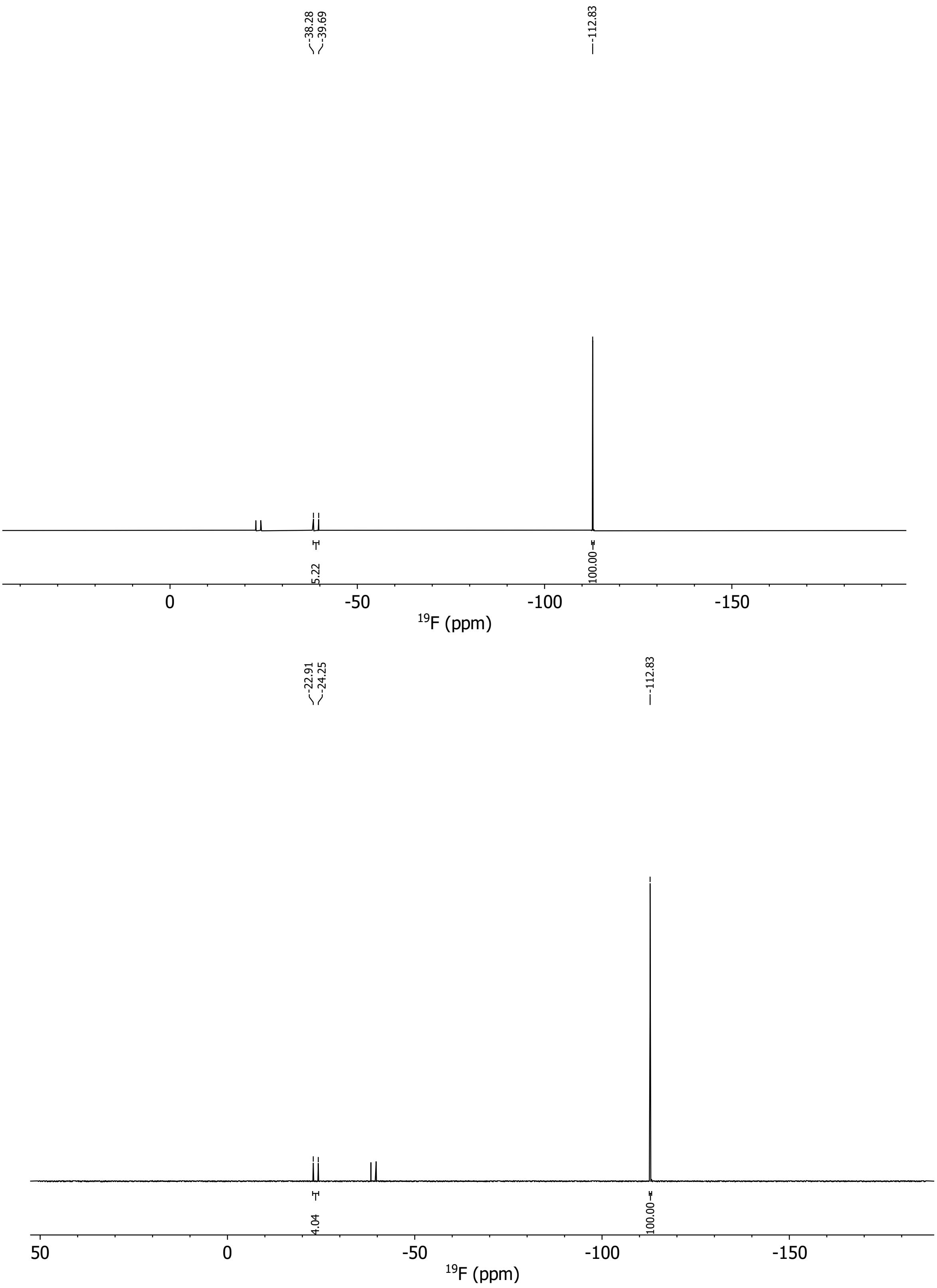

Figure S 30 *Quantitative* ^{19}F *NMR spectrum for the reaction of* ***4****-Dipp with* PPh_3Cl_2 *in* C_6D_6 *after 24 h at RT. For the yield the total amount of F in P-F bonds from a mixture of* PPh_3F_2 *(at -39.0 ppm) and monofluorinated product (at -23.6 ppm) was used. To obtain the most reliable results (i.e., ensure that the center of the spectrum is between the internal standard and the product peak), two spectra were acquired with different midpoints.*

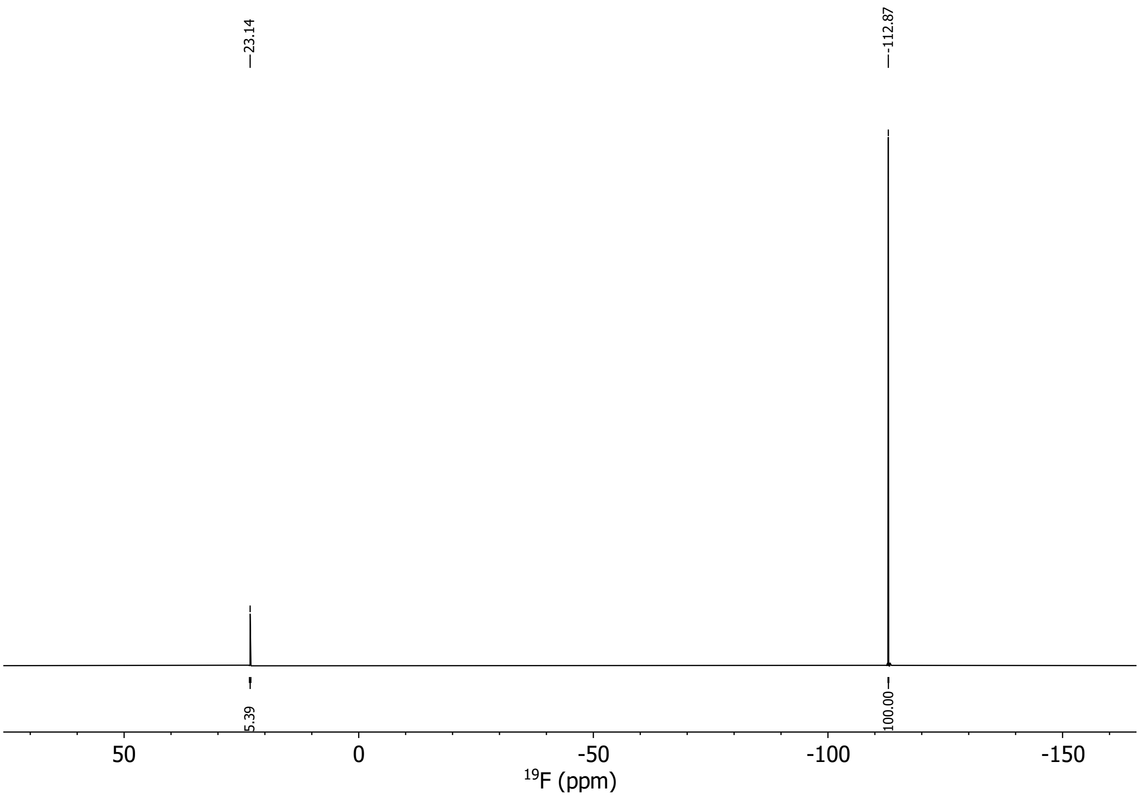

Figure S 31 *Quantitative ^{19}F NMR spectrum for the reaction of **3** with AdCOCl in C_6D_6 after 24 h at RT.*

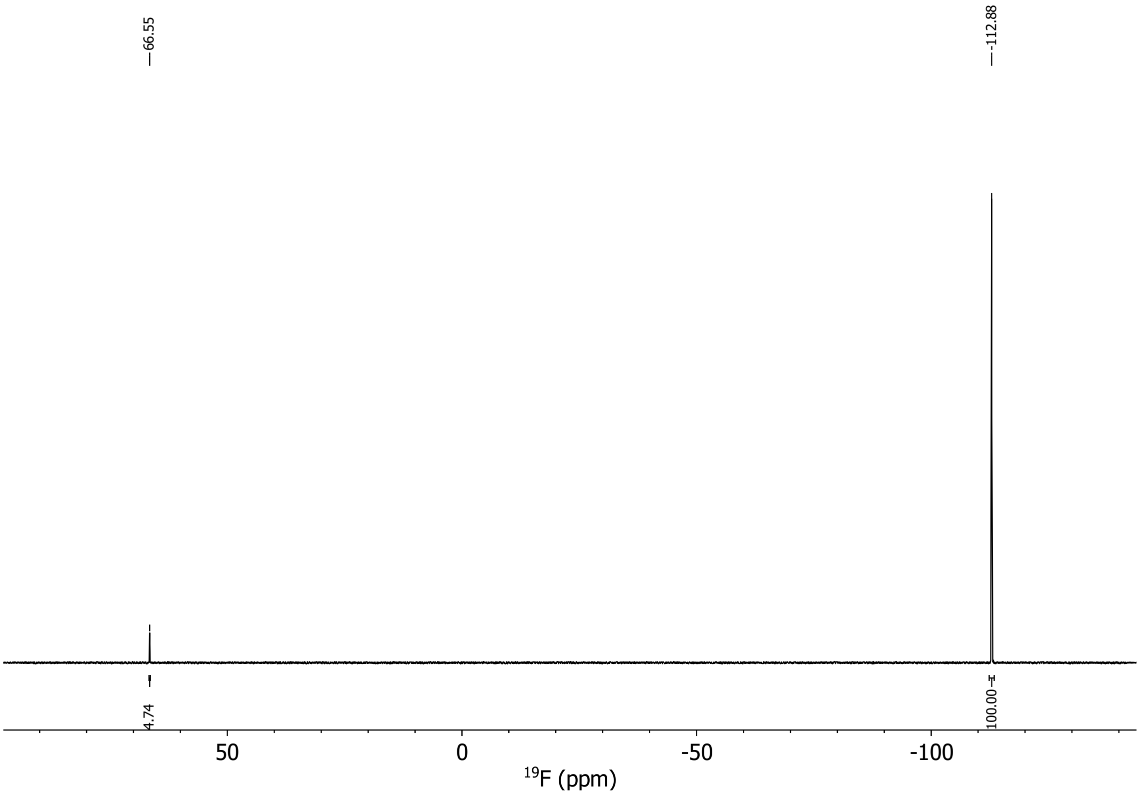

Figure S 32 *Quantitative ^{19}F NMR spectrum for the reaction of **3** with TsCl in C_6D_6 after 24 h at RT.*

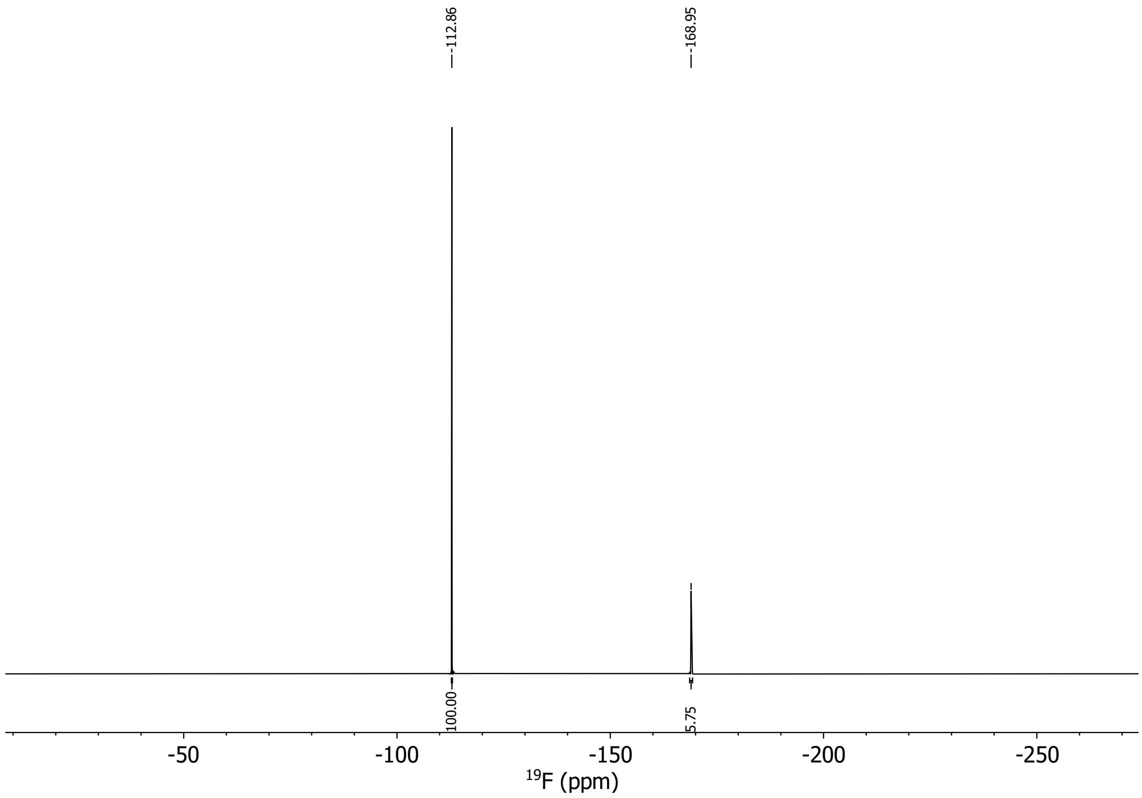

Figure S 33 *Quantitative* ^{19}*F NMR spectrum for the reaction of* ***3*** *with* Ph_3SiCl *in* C_6D_6 *after 24 h at RT.*

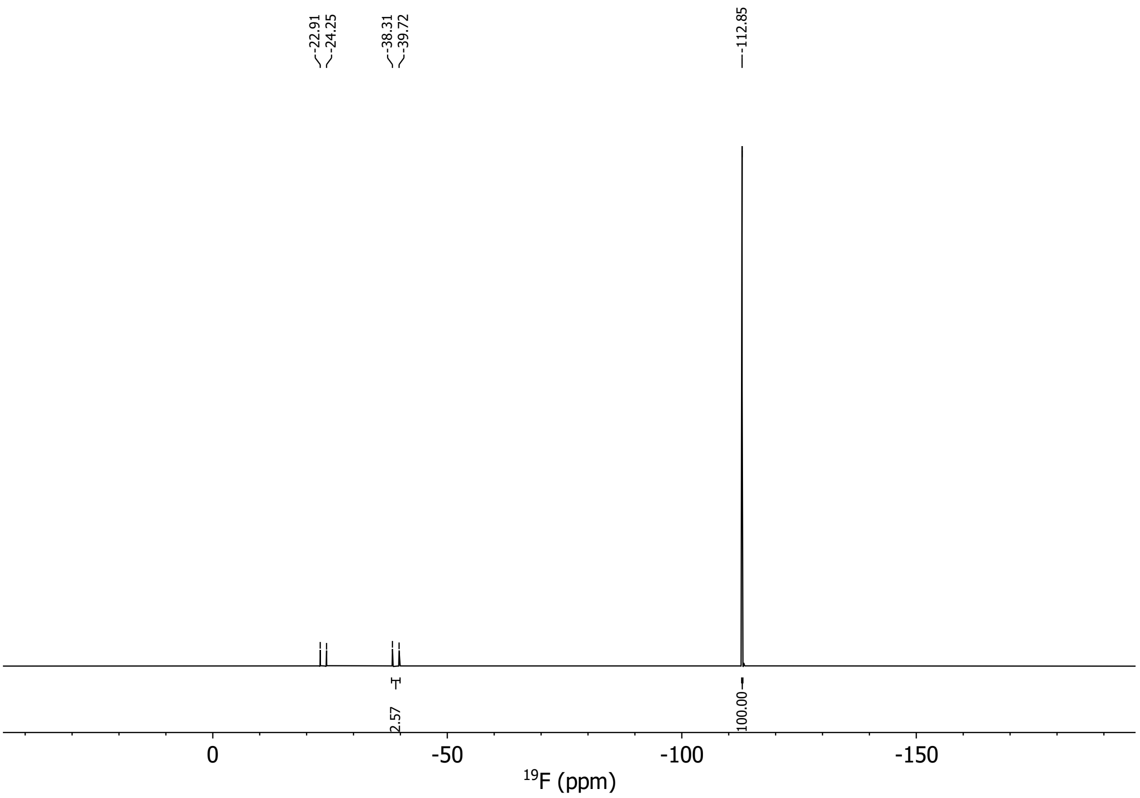

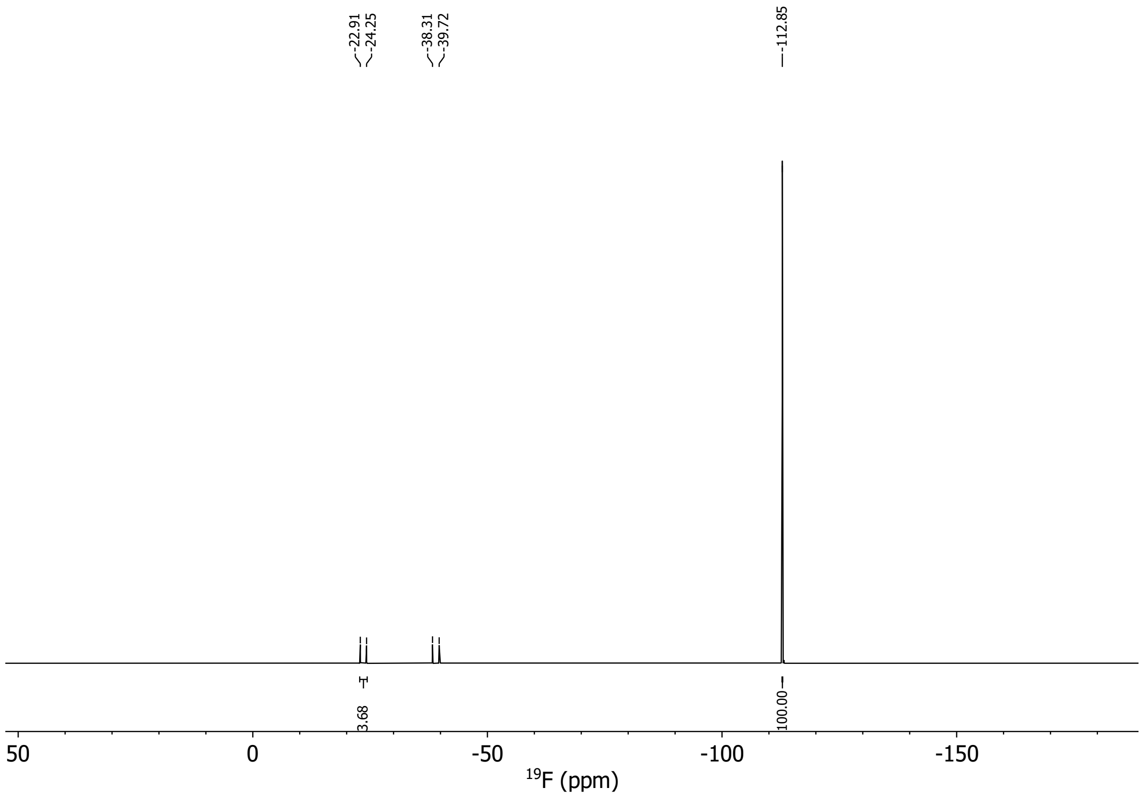

Figure S 34 *Quantitative ^{19}F NMR spectrum for the reaction of* ***3*** *with PPh_3Cl_2 in C_6D_6 after 24 h at RT. For the yield the total amount of F in P-F bonds from a mixture of PPh_3F_2 (at -39.0 ppm) and monofluorinated product (at -23.6 ppm) was used. To obtain the most reliable results (i.e., ensure that the center of the spectrum is between the internal standard and the product peak), two spectra were acquired with different midpoints.*

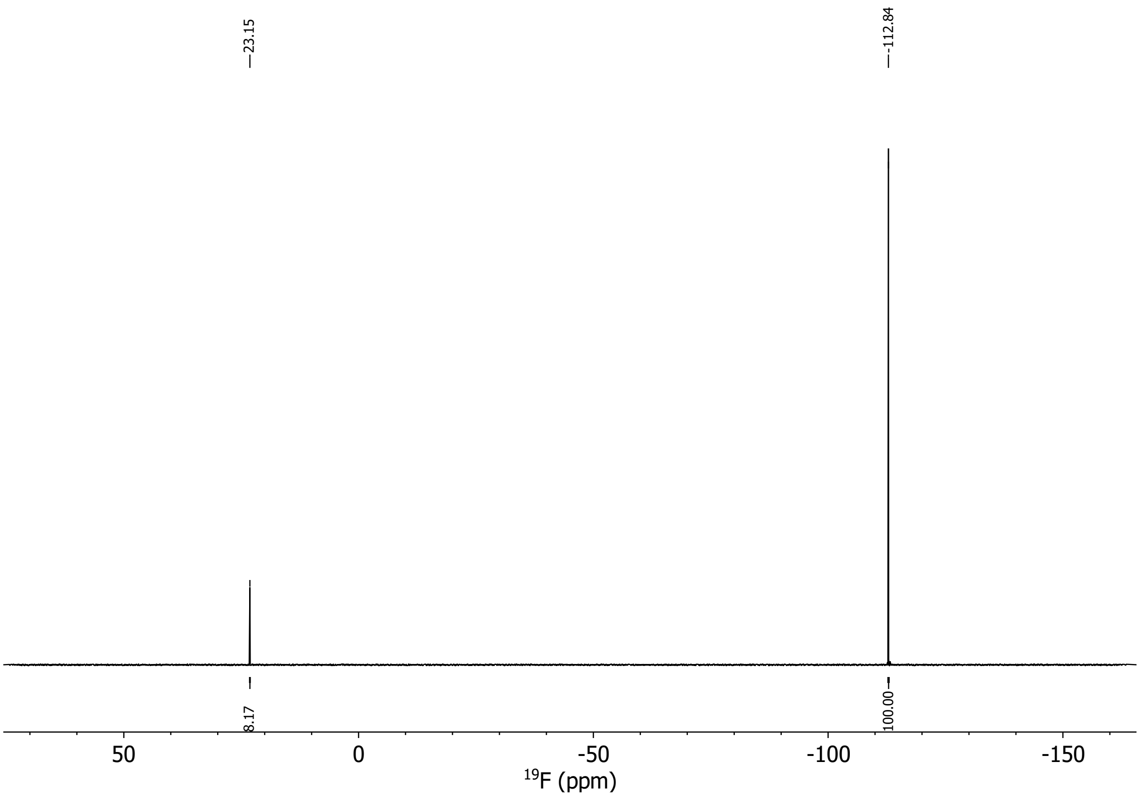

Figure S 35 *Quantitative* ^{19}F *NMR spectrum for the reaction of* ***6*** *with AdCOCl in* C_6D_6 *after 24 h at RT.*

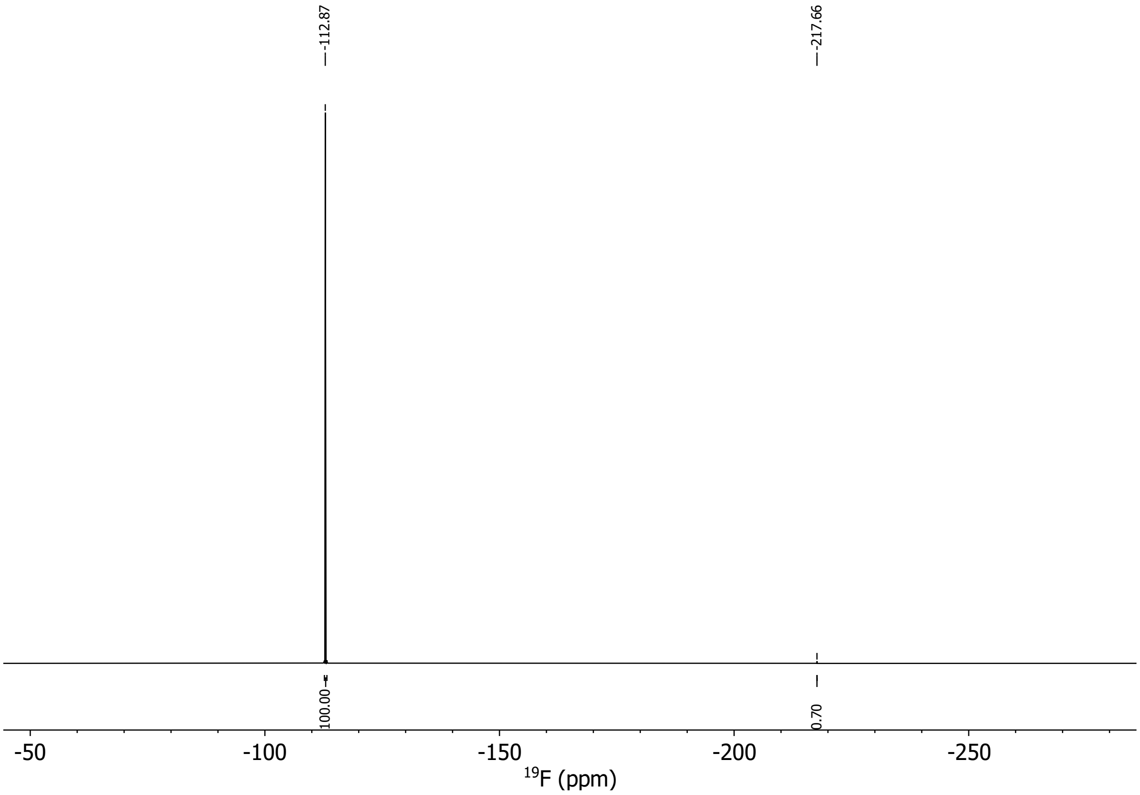

Figure S 36 *Quantitative* ^{19}F *NMR spectrum for the reaction of* ***6*** *with 1-bromopentane in* C_6D_6 *after 24 h at 80 °C.*

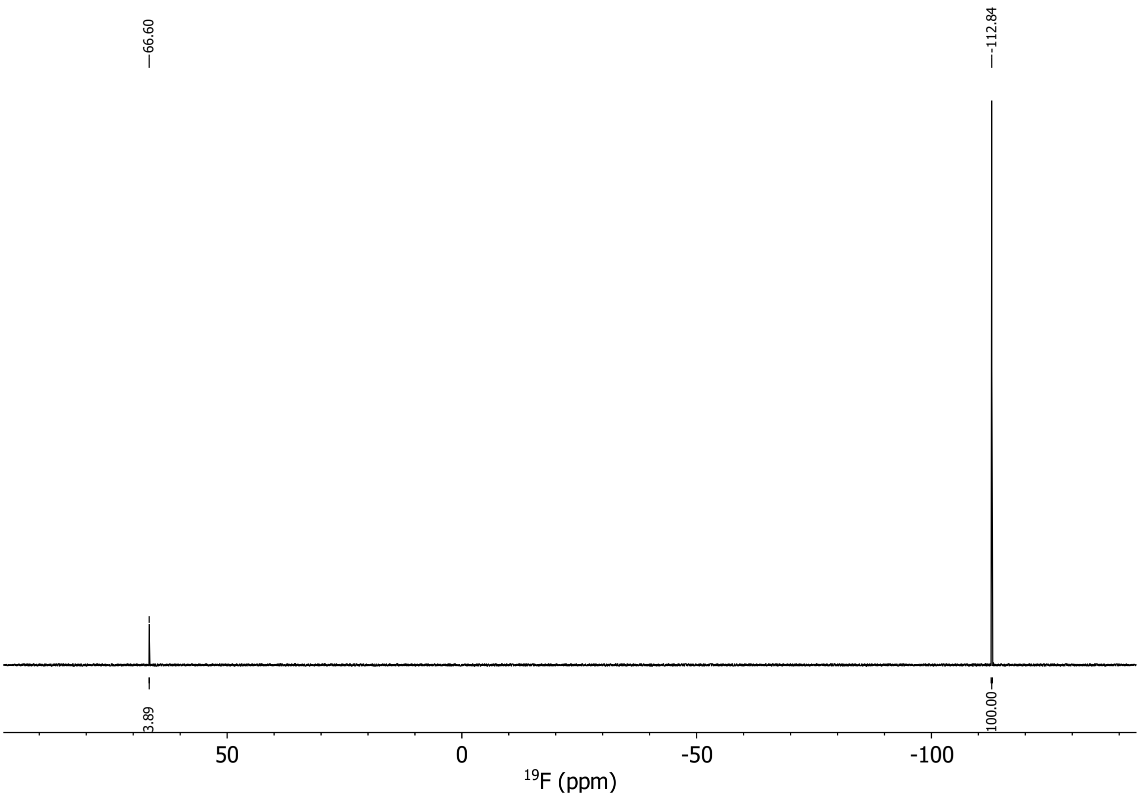

Figure S 37 *Quantitative* ^{19}*F NMR spectrum for the reaction of* ***6*** *with TsCl in* C_6D_6 *after 24 h at RT.*

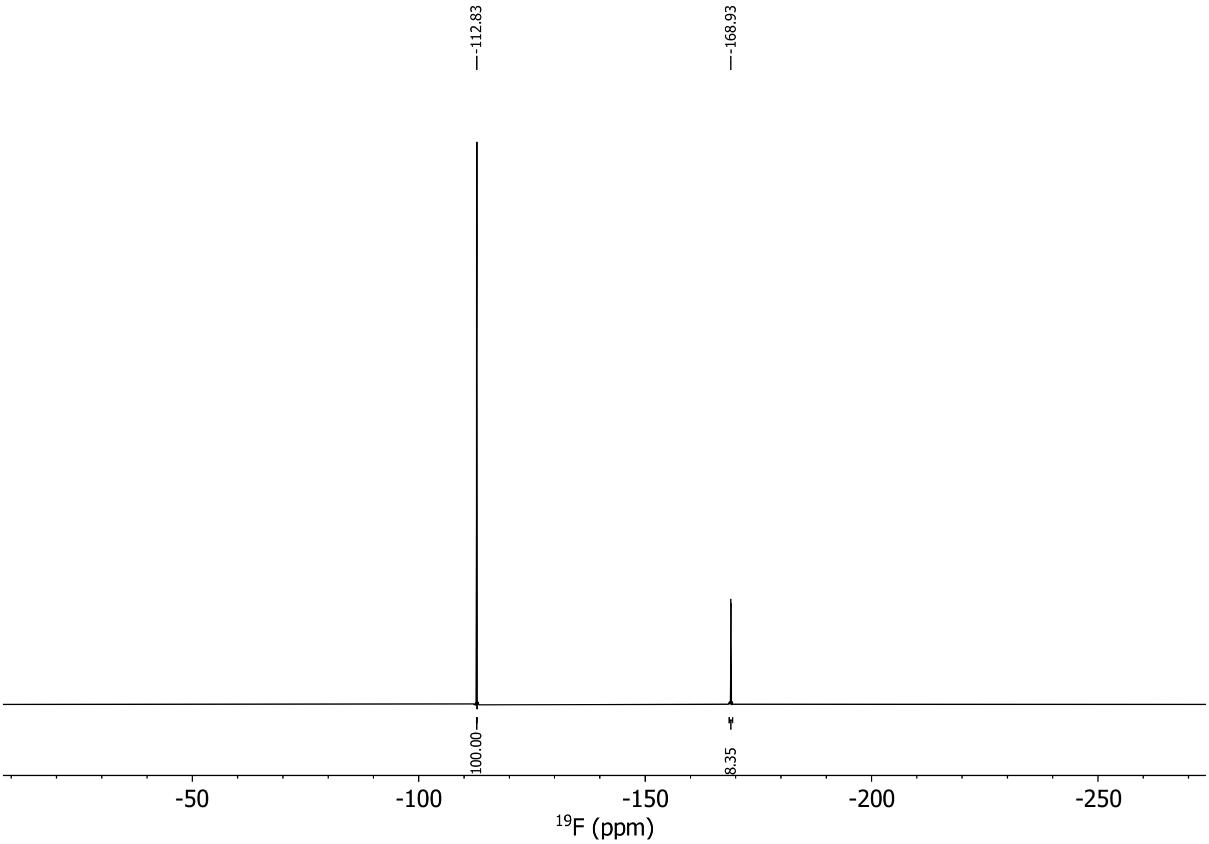

Figure S 38 *Quantitative* ^{19}*F NMR spectrum for the reaction of* ***6*** *with* Ph_3SiCl *in* C_6D_6 *after 24 h at RT.*

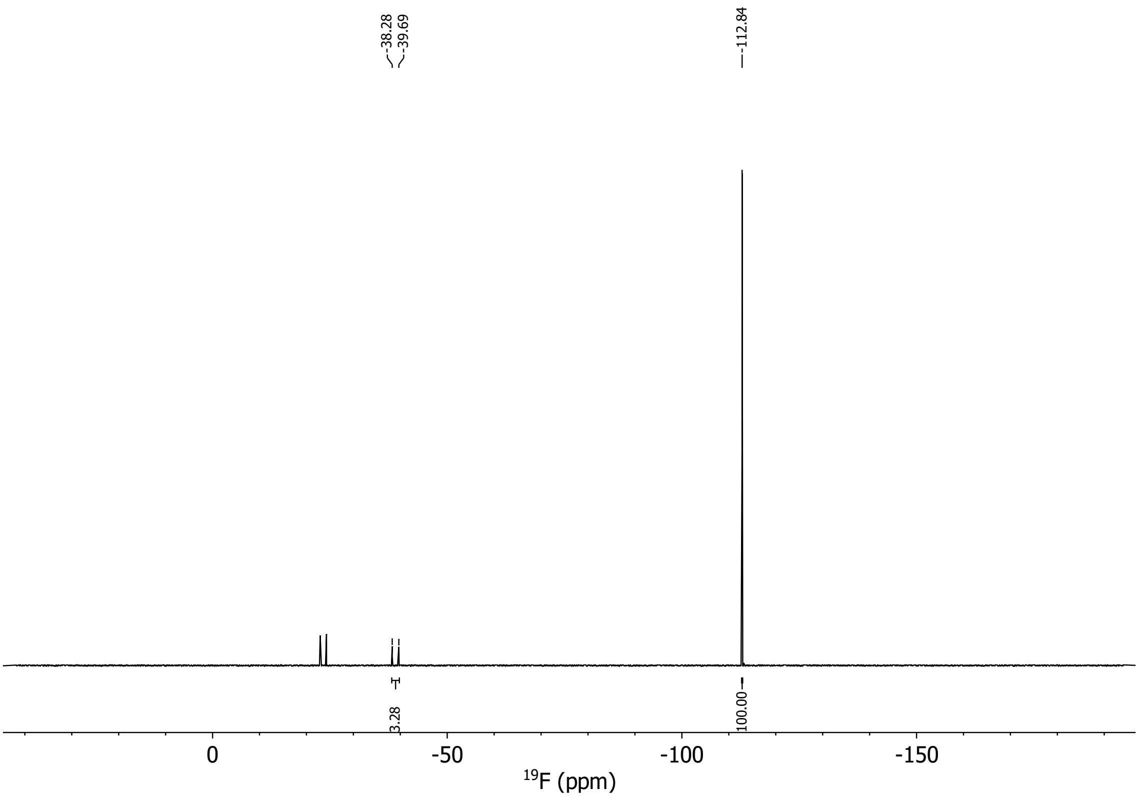

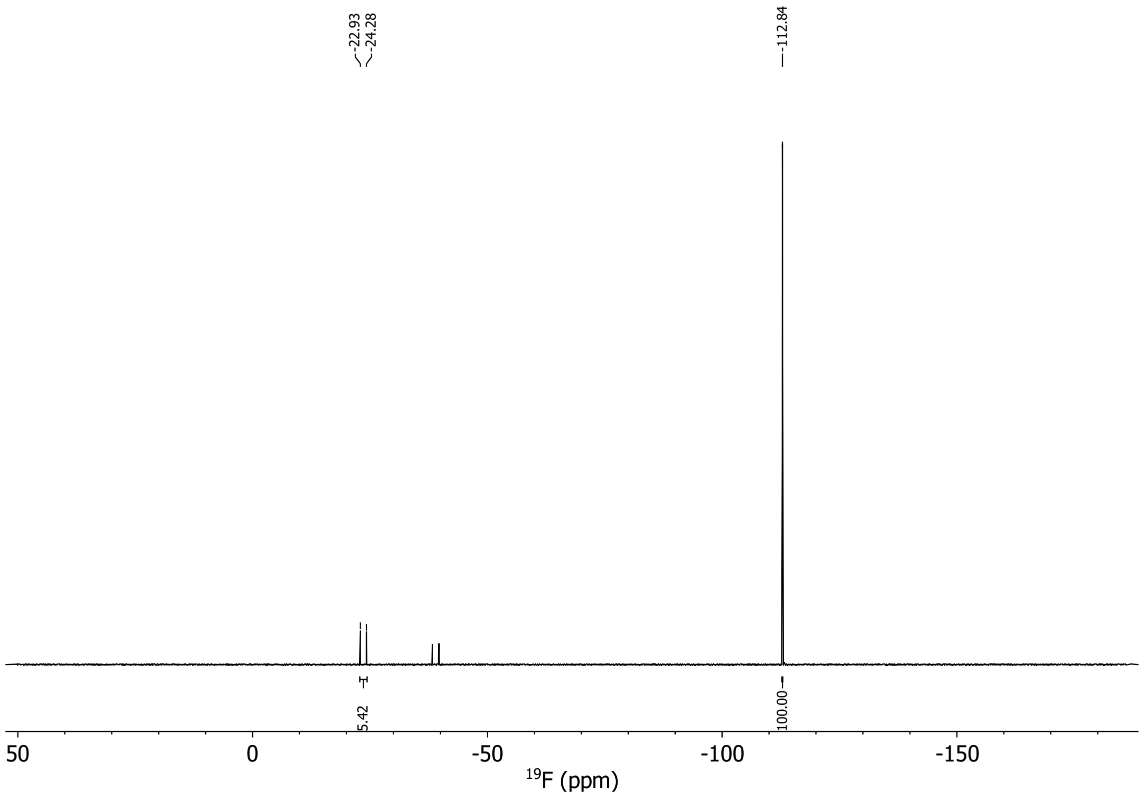

Figure S 39 *Quantitative* ^{19}*F NMR spectrum for the reaction of* ***6*** *with* PPh_3Cl_2 *in* C_6D_6 *after 24 h at RT. For the yield the total amount of F in P-F bonds from a mixture of* PPh_3F_2 *(at -39.0 ppm) and monofluorinated product (at -23.6 ppm) was used. To obtain the most reliable results (i.e., ensure that the center of the spectrum is between the internal standard and the product peak), two spectra were acquired with different midpoints.*

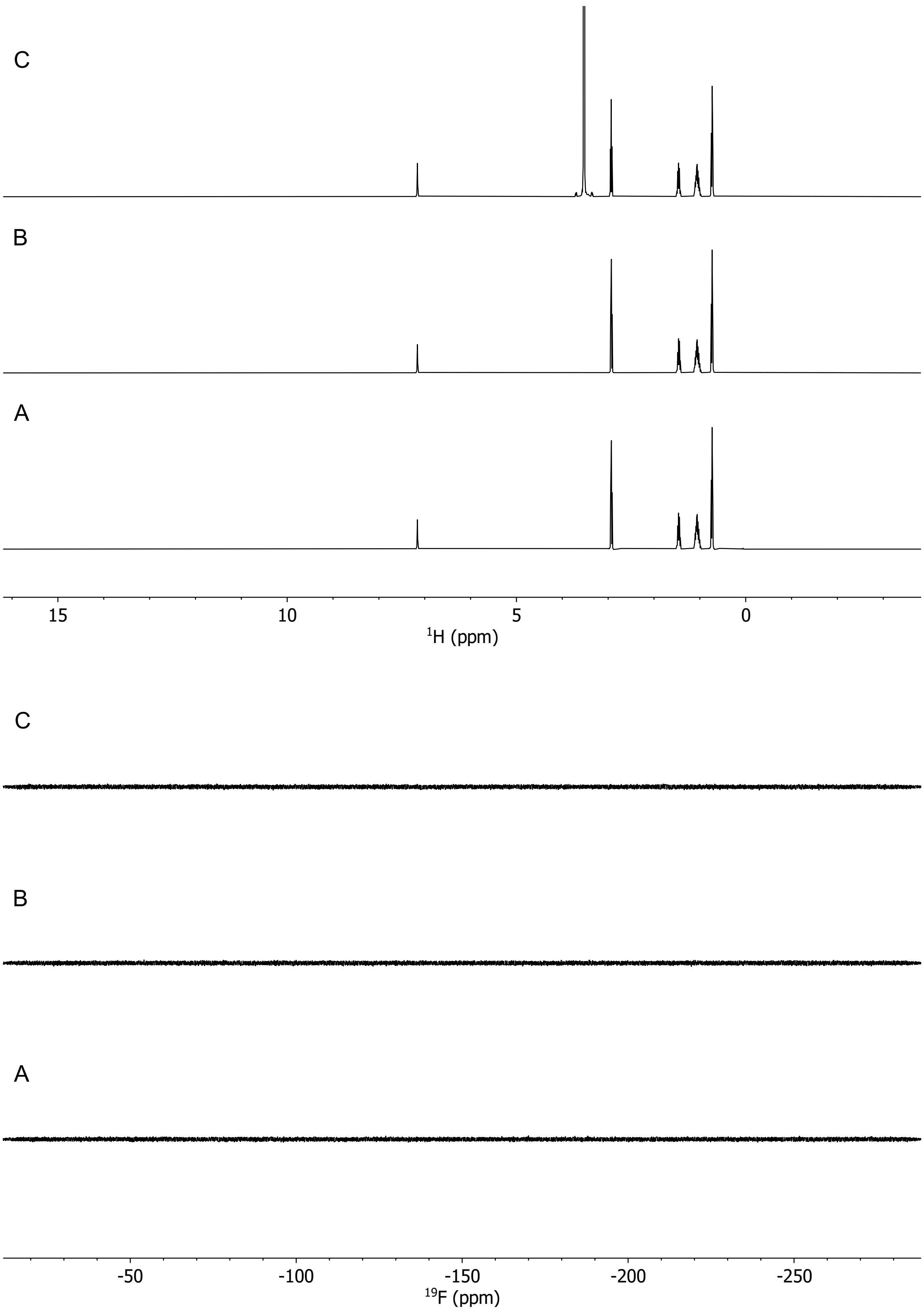

Figure S 40 *Example of control reactions: ^{1}H NMR and ^{19}F NMR spectra of the reactions of Me_3SnF (A), KF (B), KF + 18-crown-6 (C) with 1-bromopentane in C_6D_6 showing no product formation after 24 h at 80 °C.*

NMR spectra of synthesized compounds

Me_3SnF

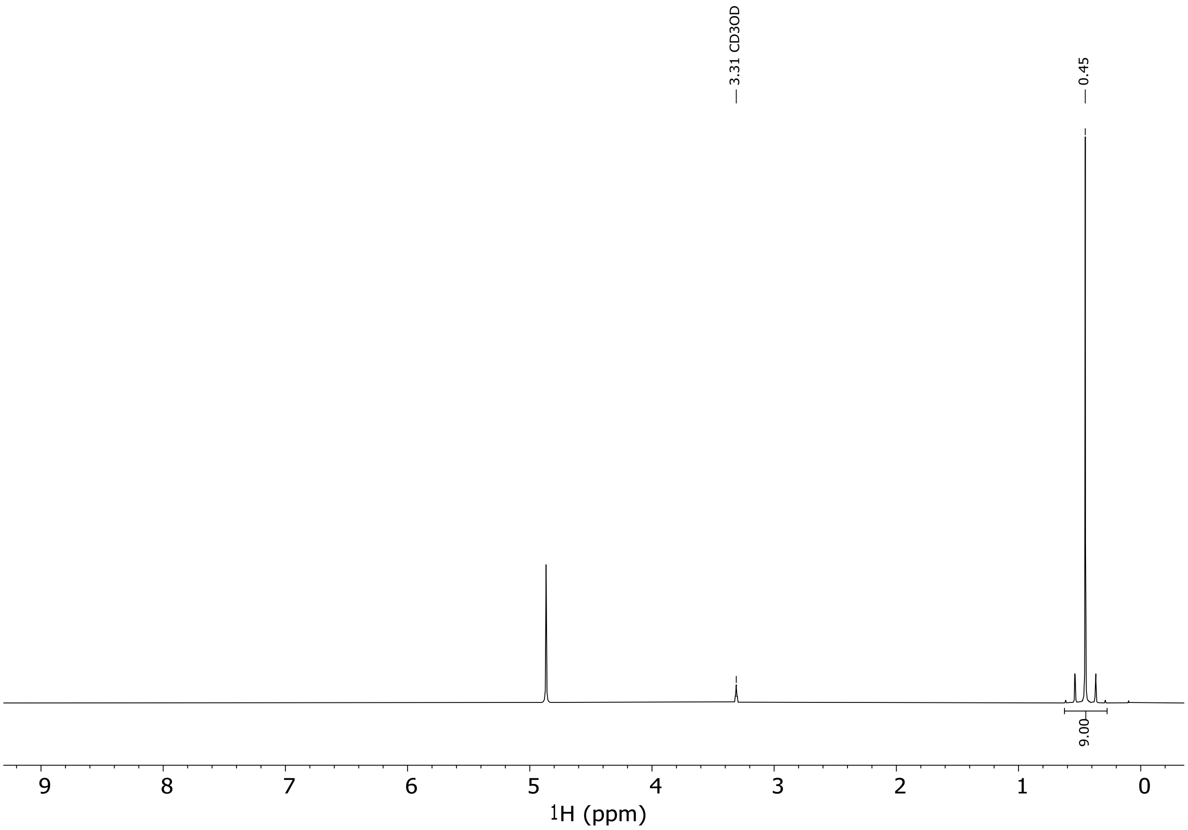

Figure S 41 *^1H NMR spectrum of Me_3SnF in MeOD-d_4.*

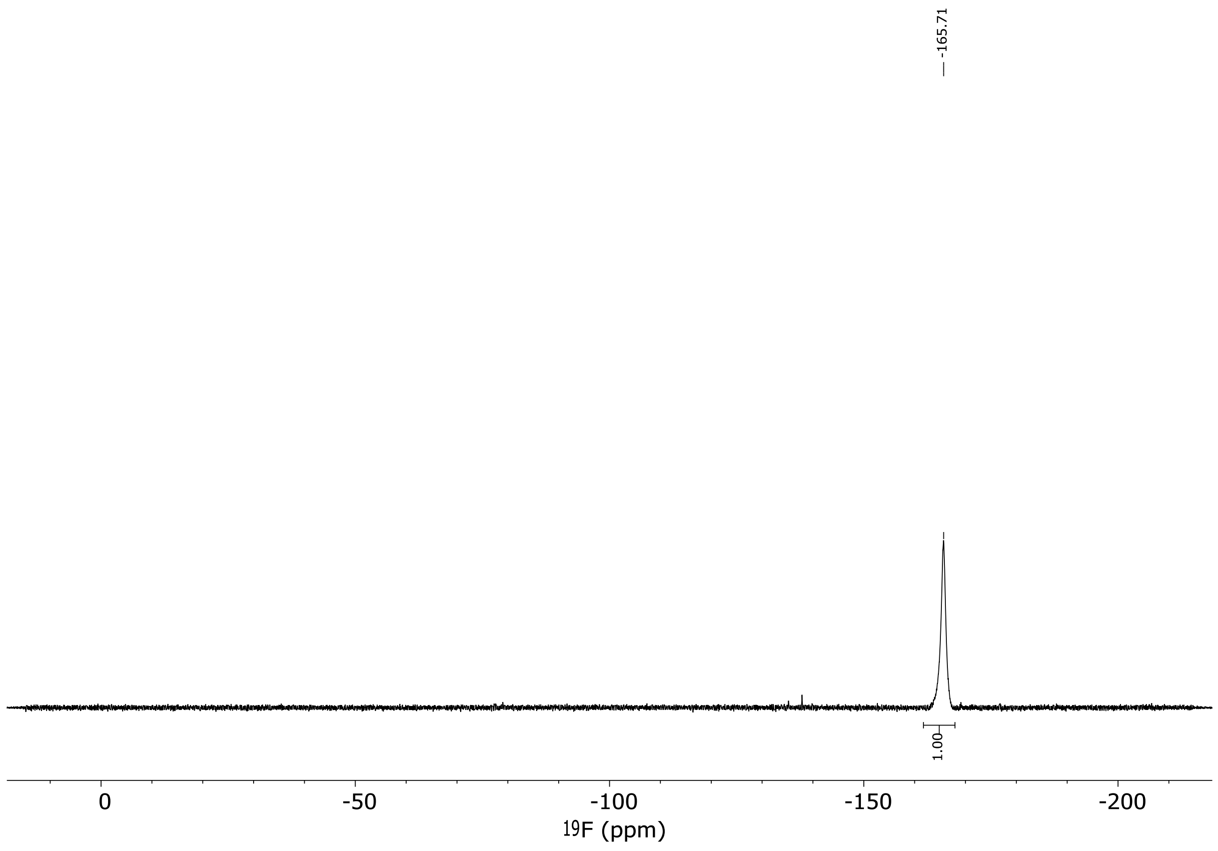

Figure S 42 *^{19}F NMR spectrum of Me_3SnF in MeOD-d_4.*

Ca(HMDS)$_2$(thf)$_2$

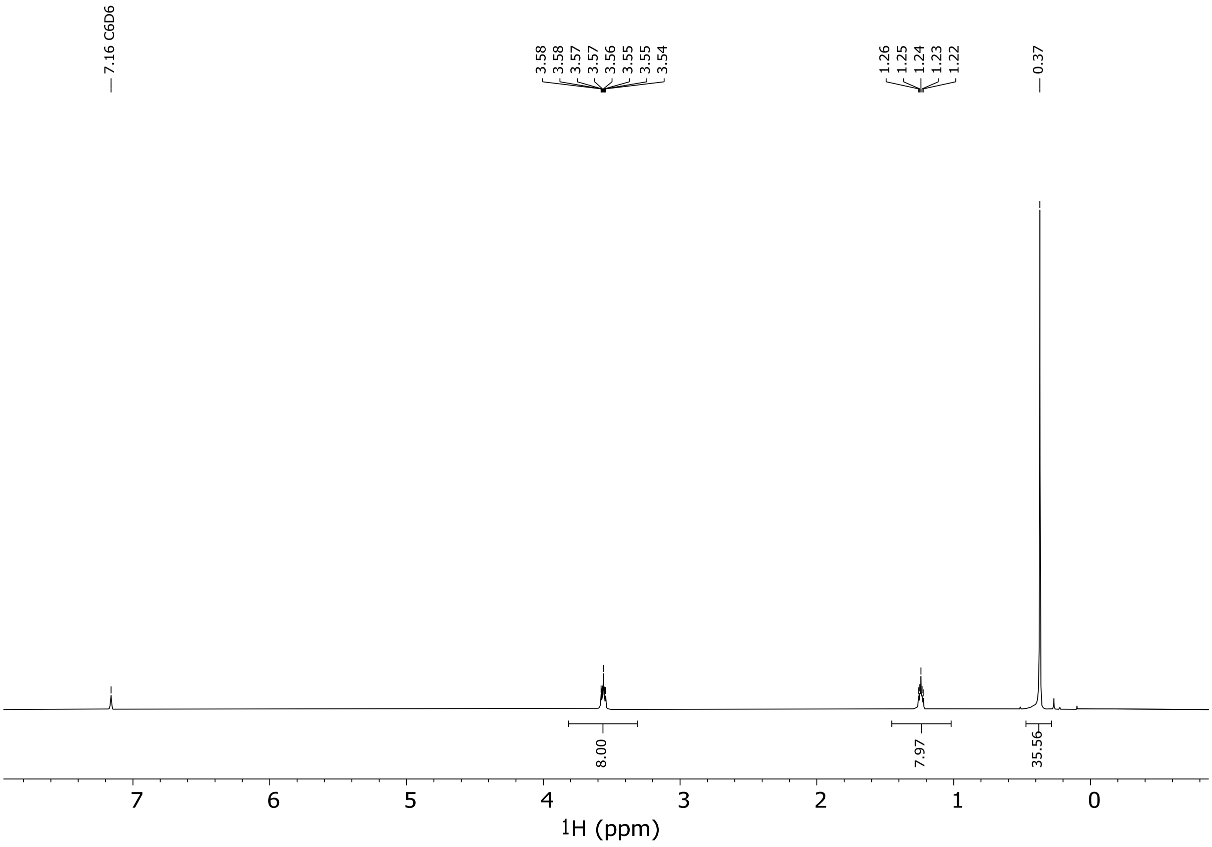

Figure S 43 *^{1}H NMR spectrum of Ca(HMDS)$_2$(thf)$_2$ in C_6D_6.*

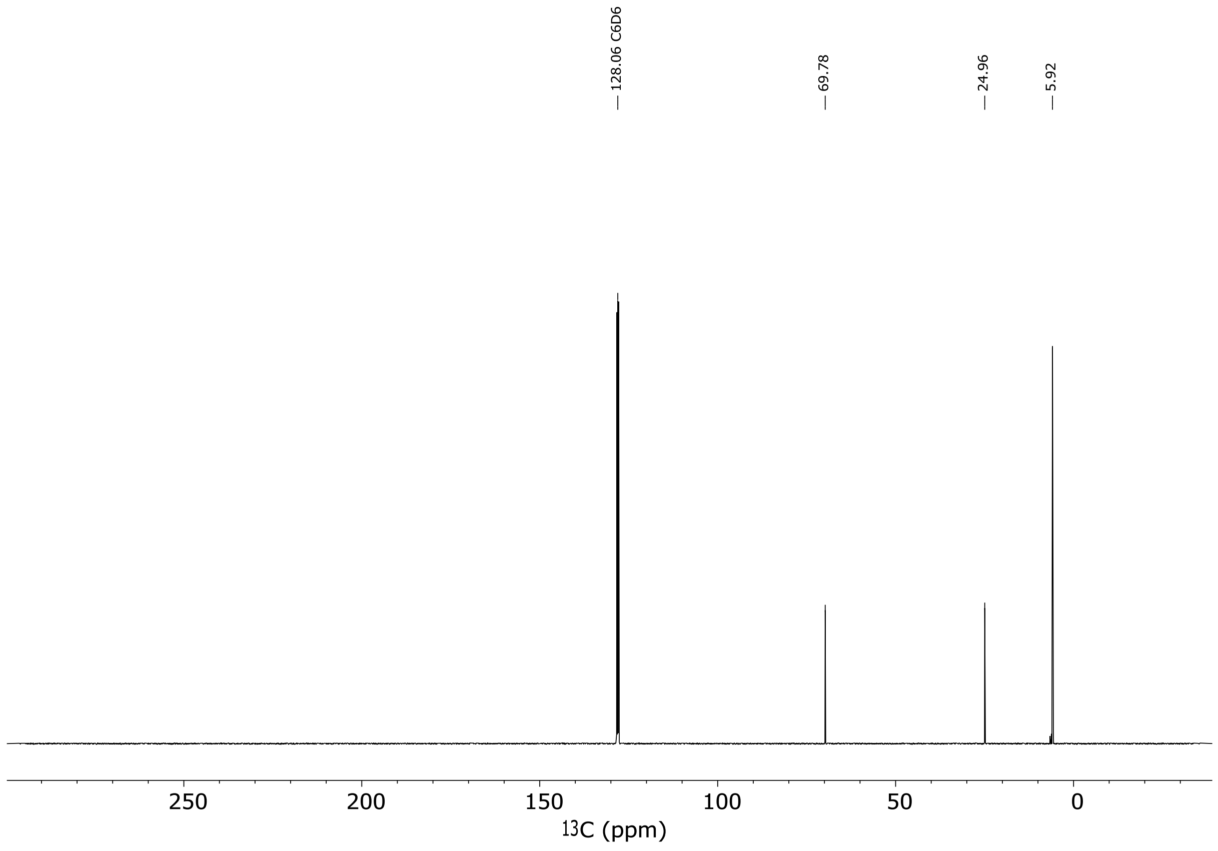

Figure S 44 *$^{13}C\{^{1}H\}$ NMR spectrum of $Ca(HMDS)_2(thf)_2$ in C_6D_6.*

Cs(HMDS)

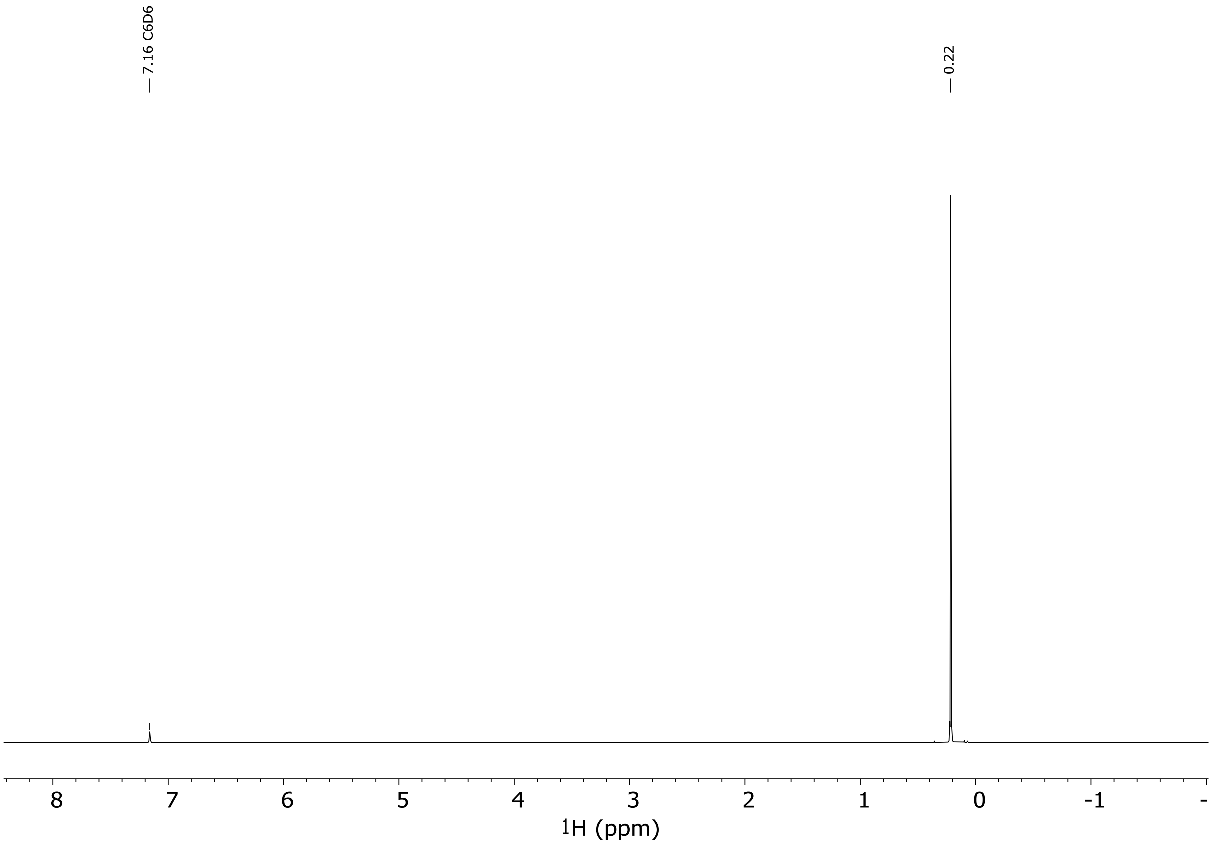

Figure S 45 *¹H NMR spectrum of Cs(HMDS) in C_6D_6.*

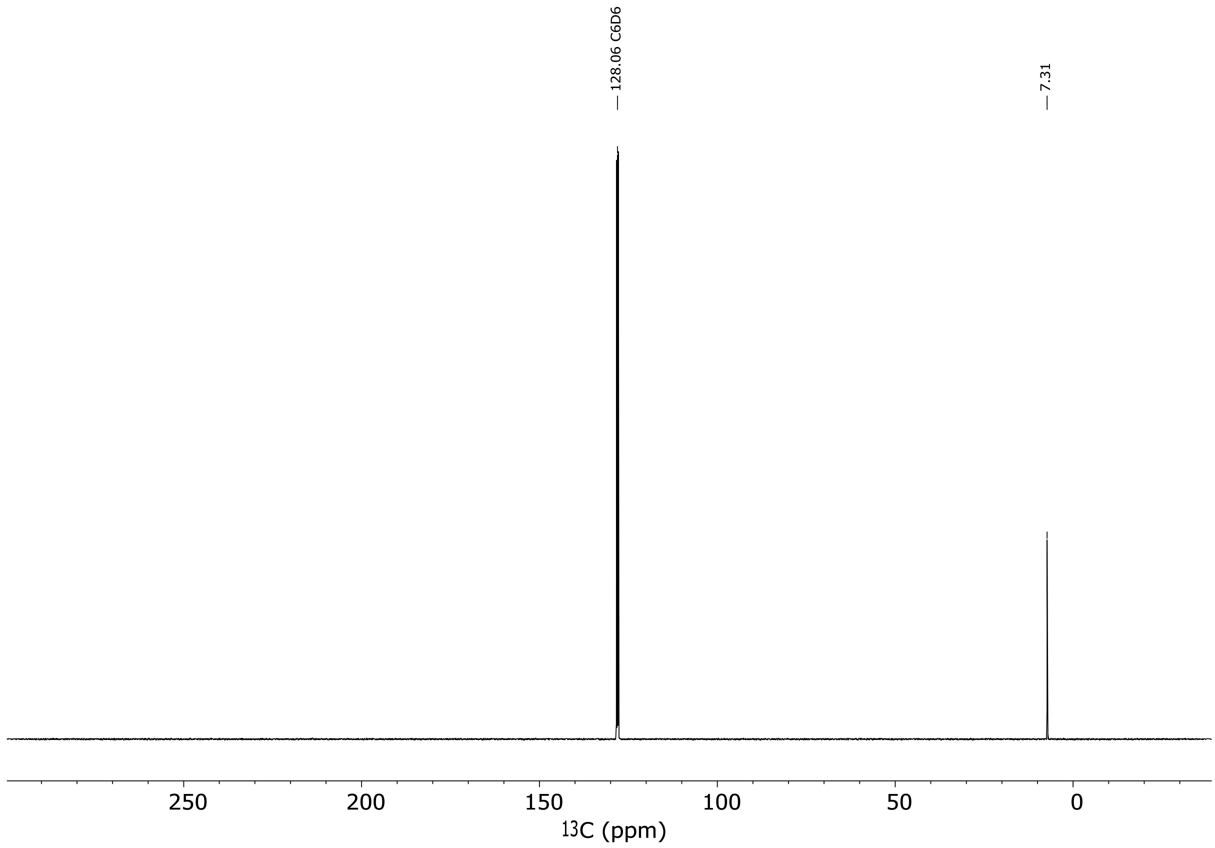

Figure S 46 *$^{13}C\{^1H\}$ NMR spectrum of Cs(HMDS) in C_6D_6.*

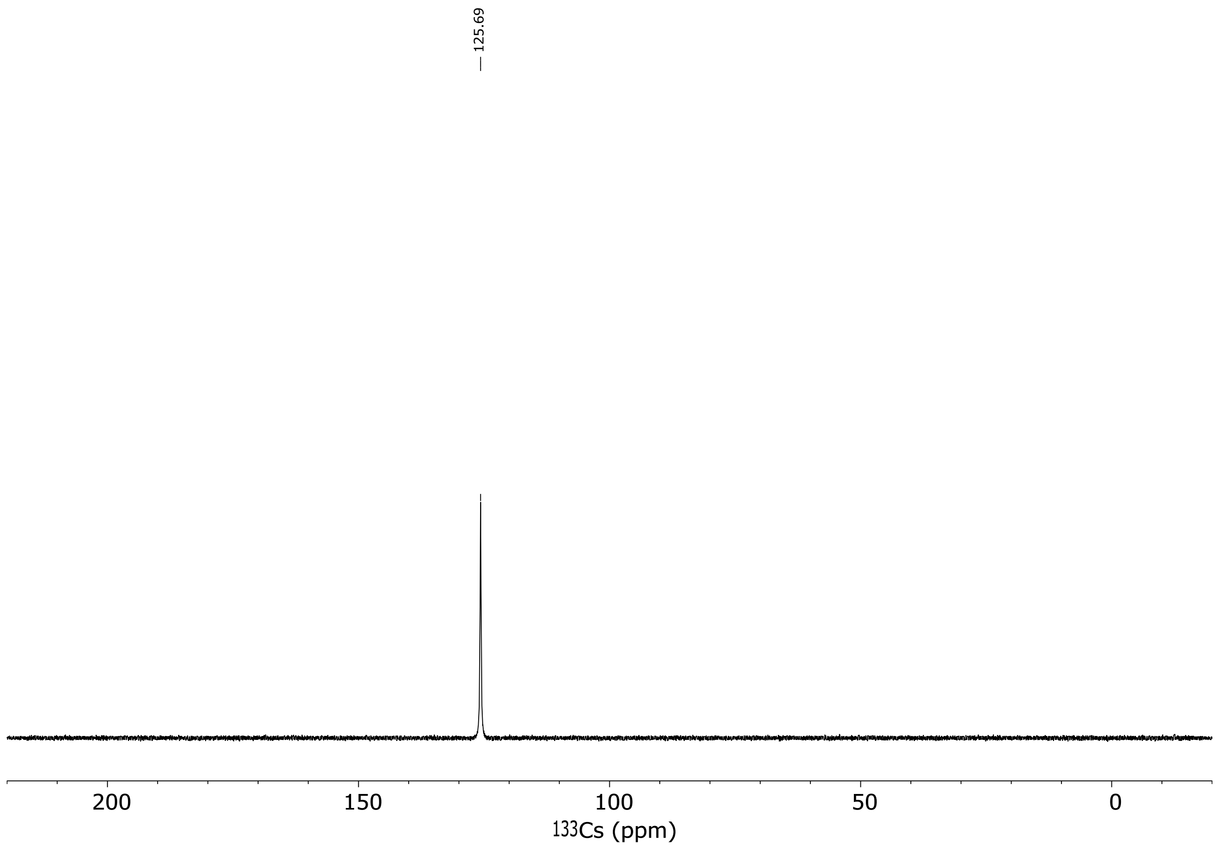

Figure S 47 *^{133}Cs NMR spectrum of Cs(HMDS) in C_6D_6.*

1-Dipp

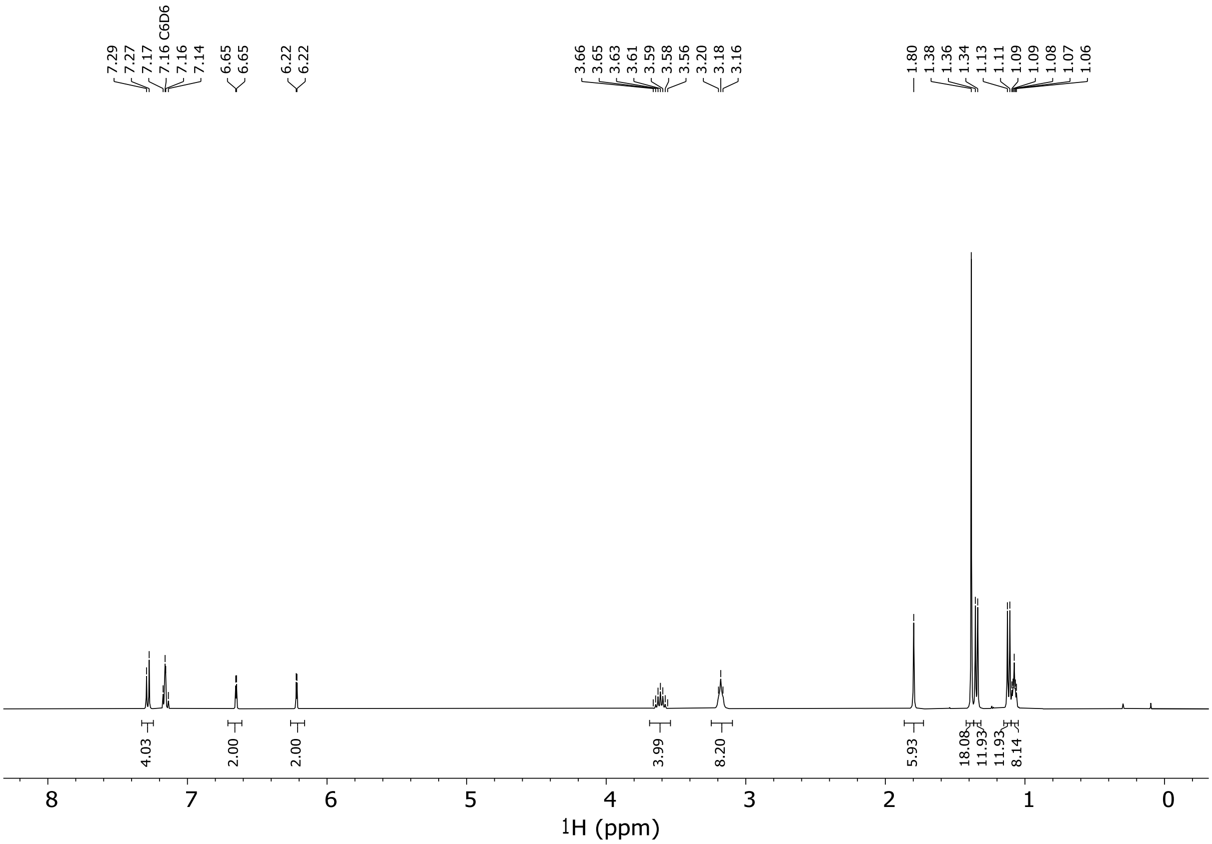

Figure S 48 *^{1}H NMR spectrum of **1-Dipp** in C_6D_6.*

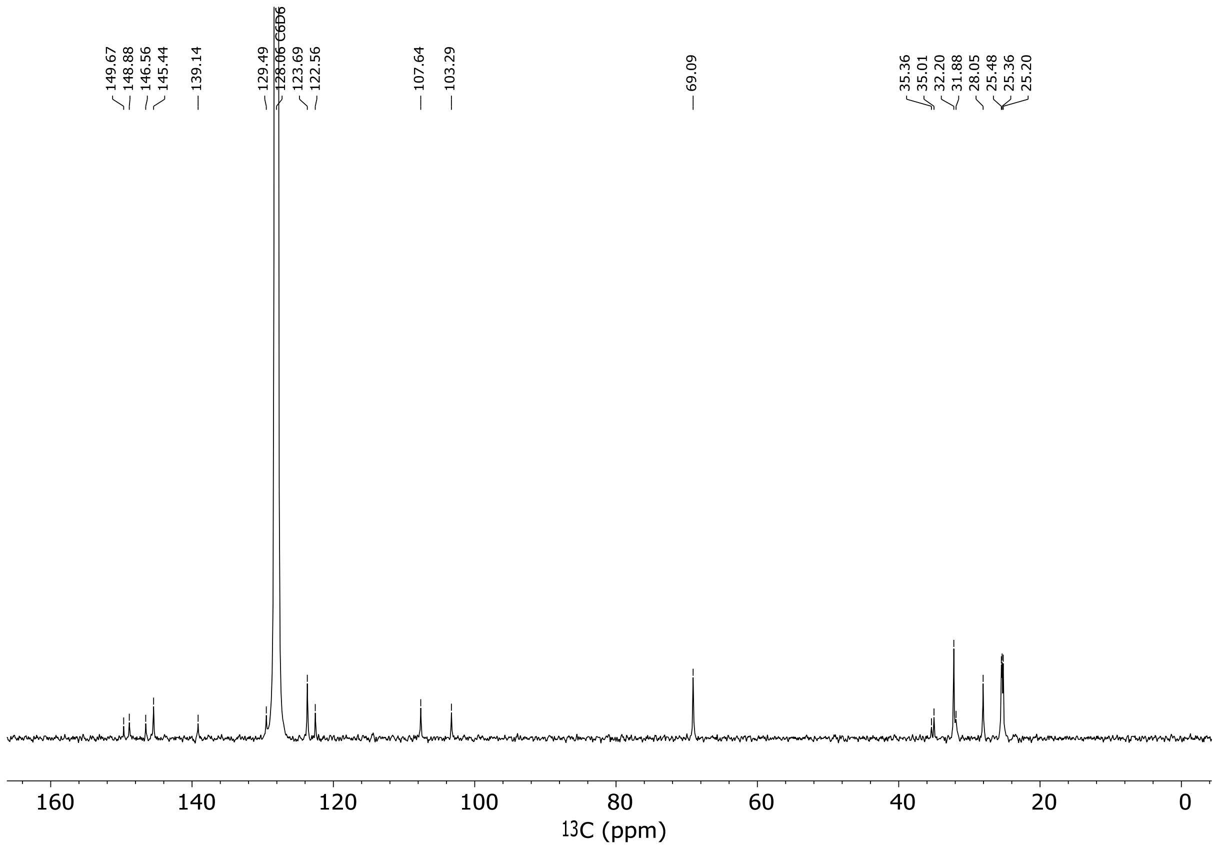

Figure S 49 *$^{13}C\{^1H\}$ NMR spectrum of **1-Dipp** in C_6D_6.*

1-Trip

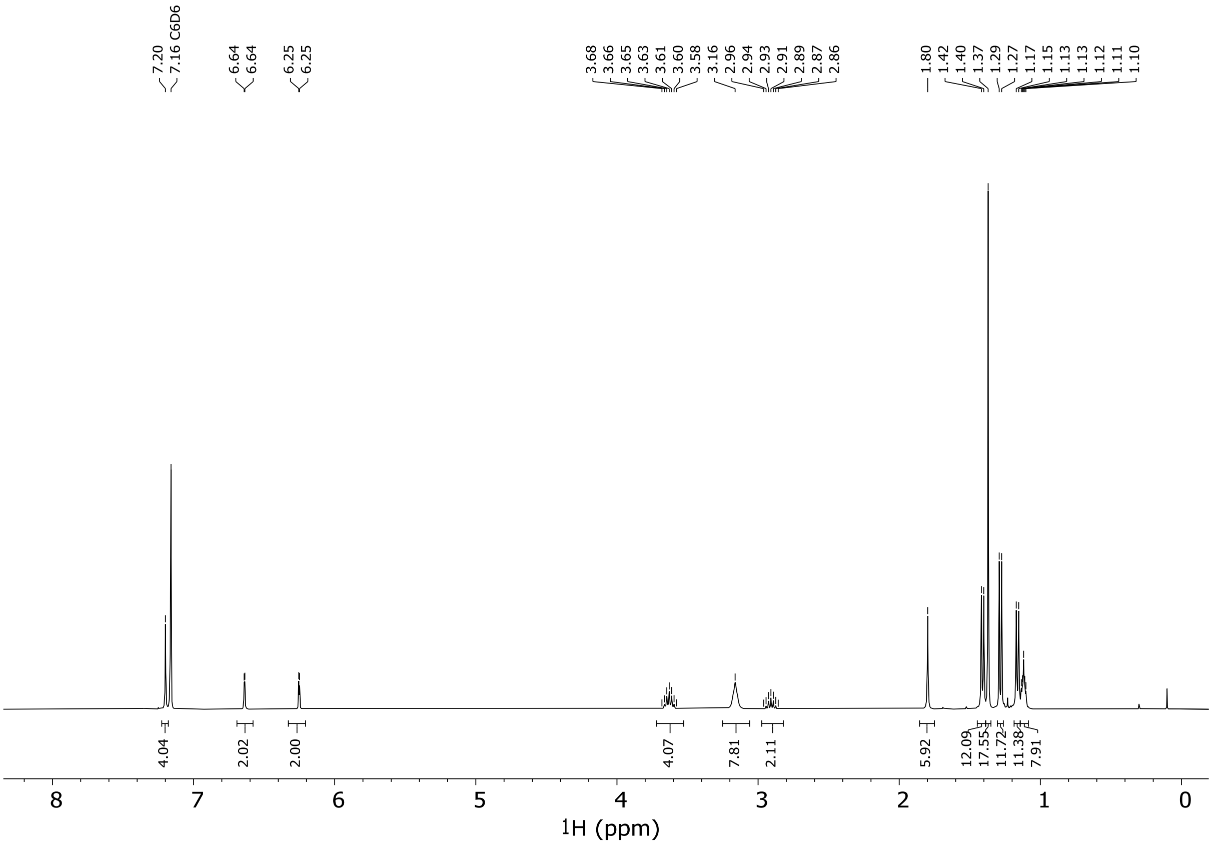

Figure S 50 *^{1}H NMR spectrum of* ***1-Trip*** *in C_6D_6.*

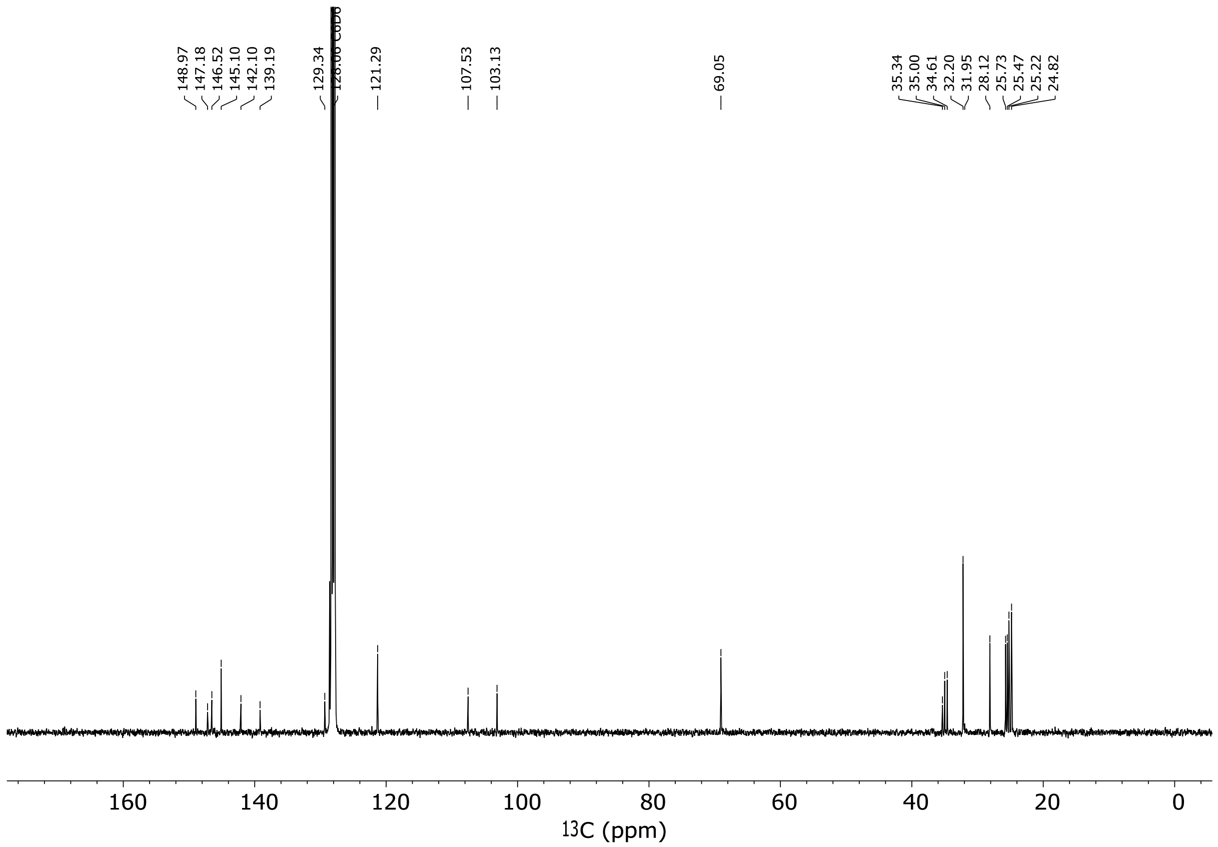

***Figure S 51** $^{13}C\{^{1}H\}$ NMR spectrum of **1-Trip** in C_6D_6.*

2

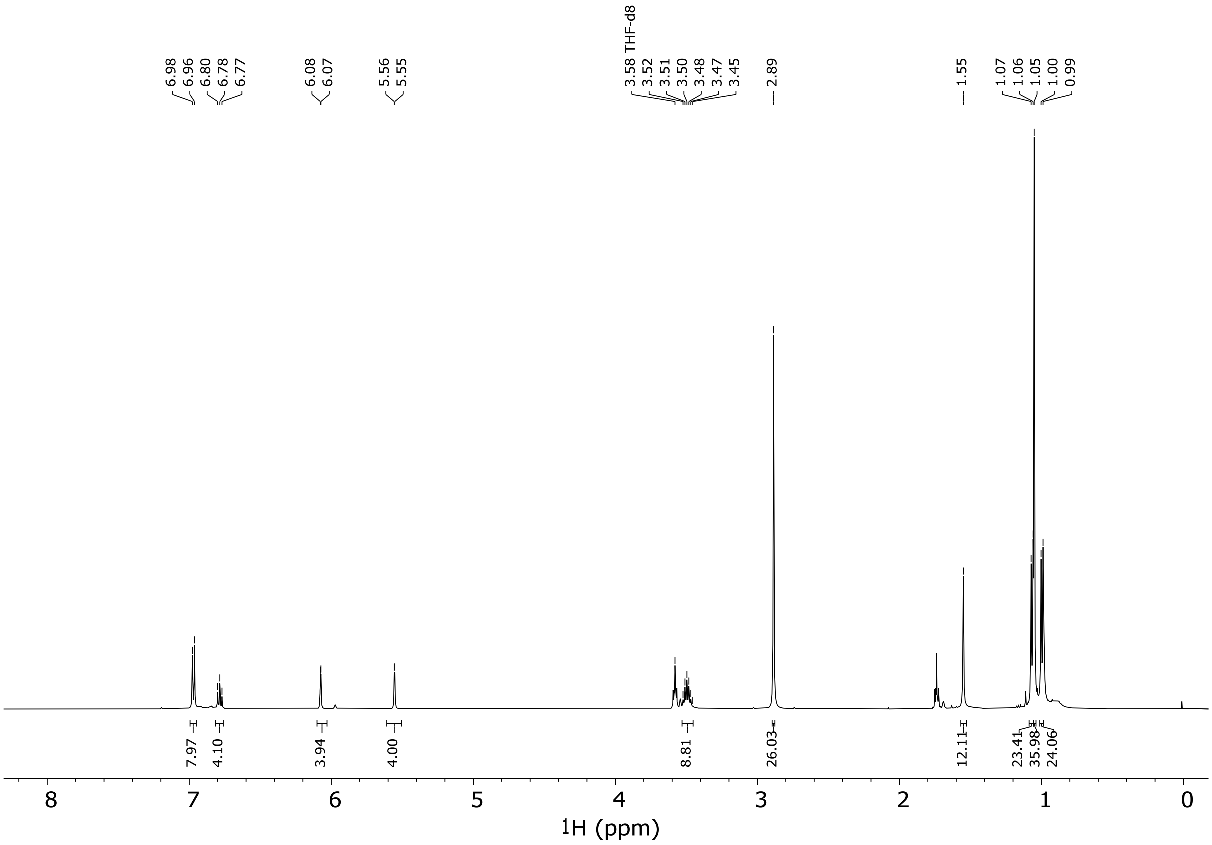

Figure S 52 *^{1}H NMR spectrum of* ***2*** *in thf-d_8.*

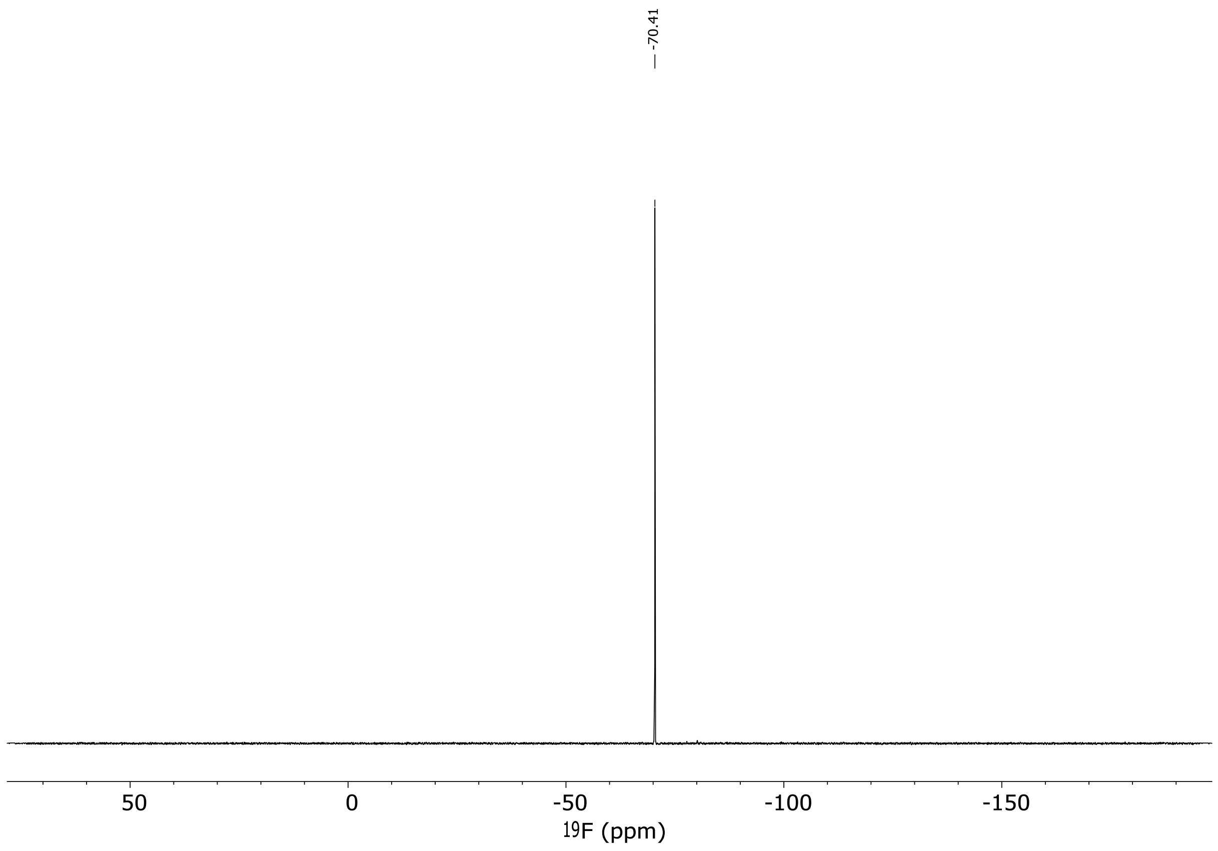

Figure S 53 *^{19}F NMR spectrum of* ***2*** *in thf-d_8.*

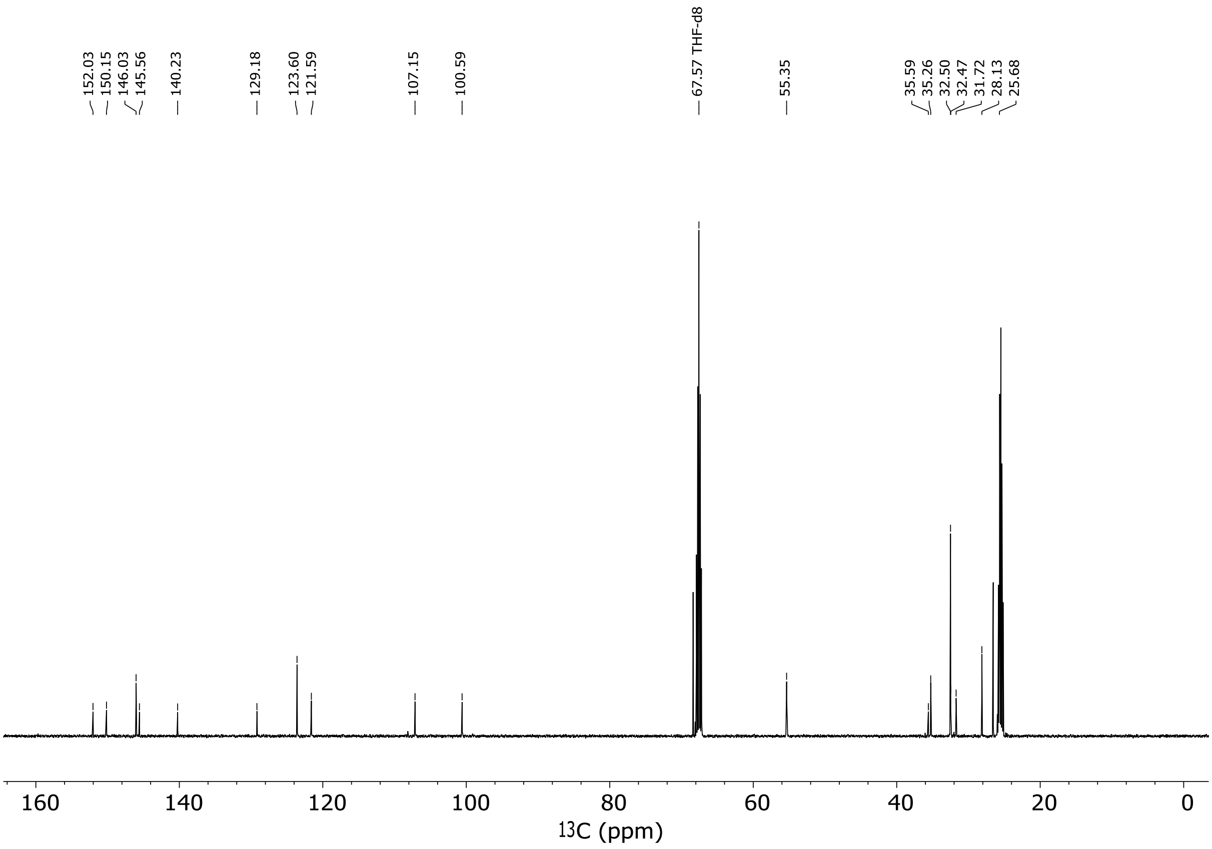

Figure S 54 *$^{13}C\{^1H\}$ NMR spectrum of* ***2*** *in thf-d_8.*

3

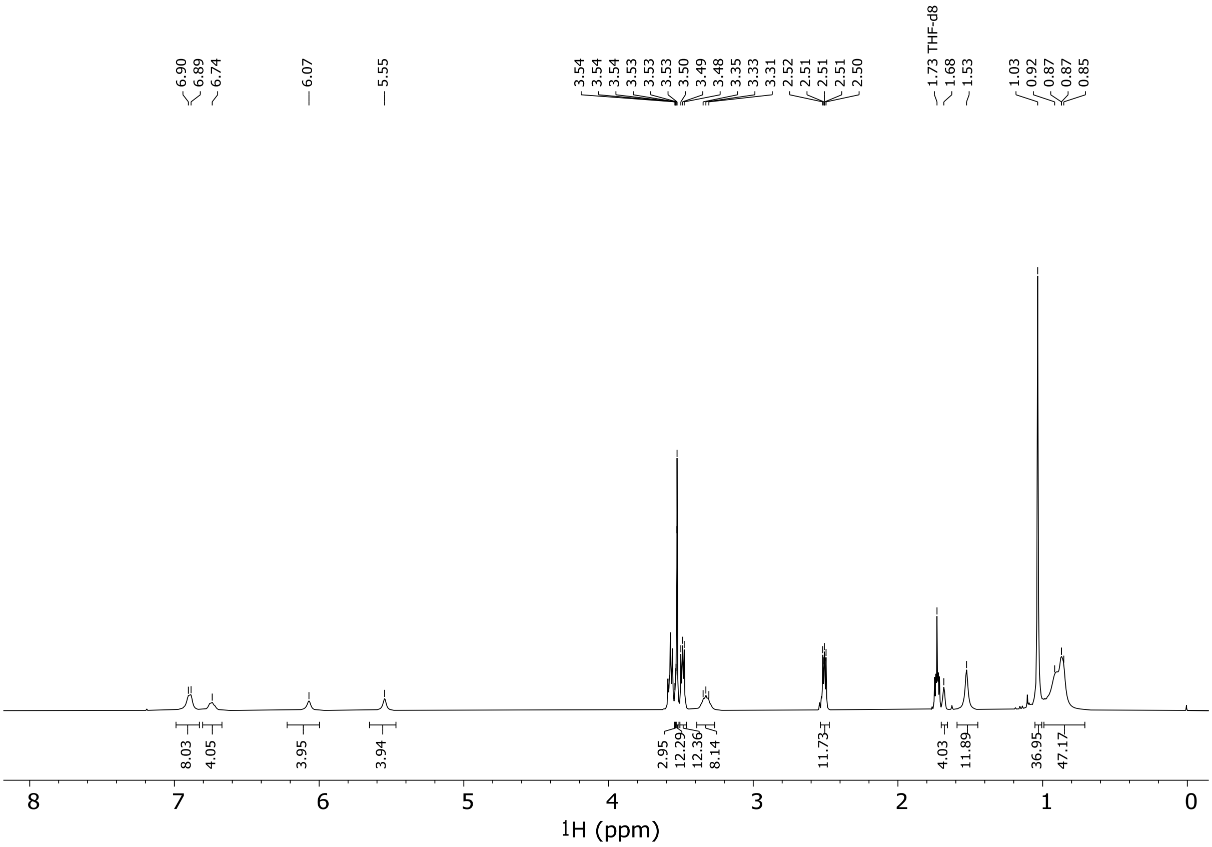

Figure S 55 *^{1}H NMR spectrum of **3** in thf-d_8 at RT.*

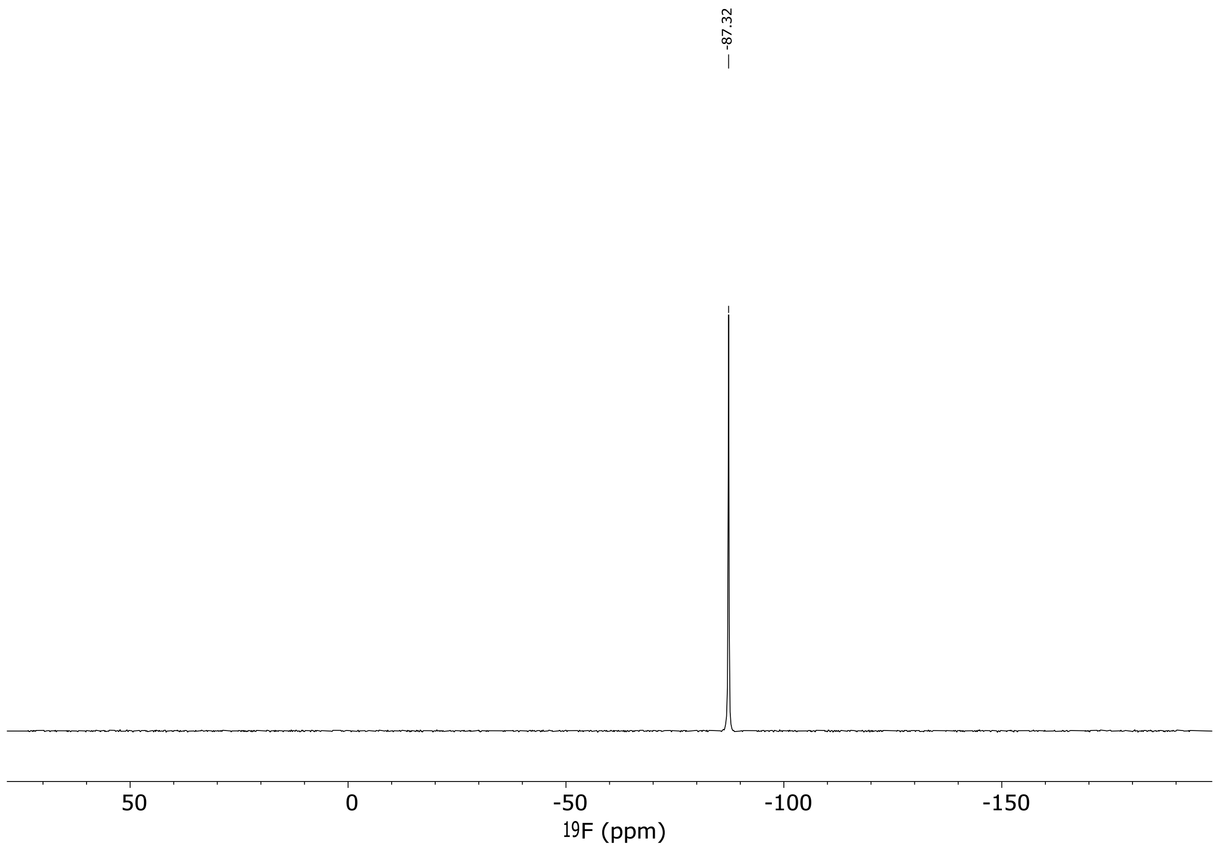

Figure S 56 *^{19}F NMR spectrum of **3** in thf-d_8 at RT.*

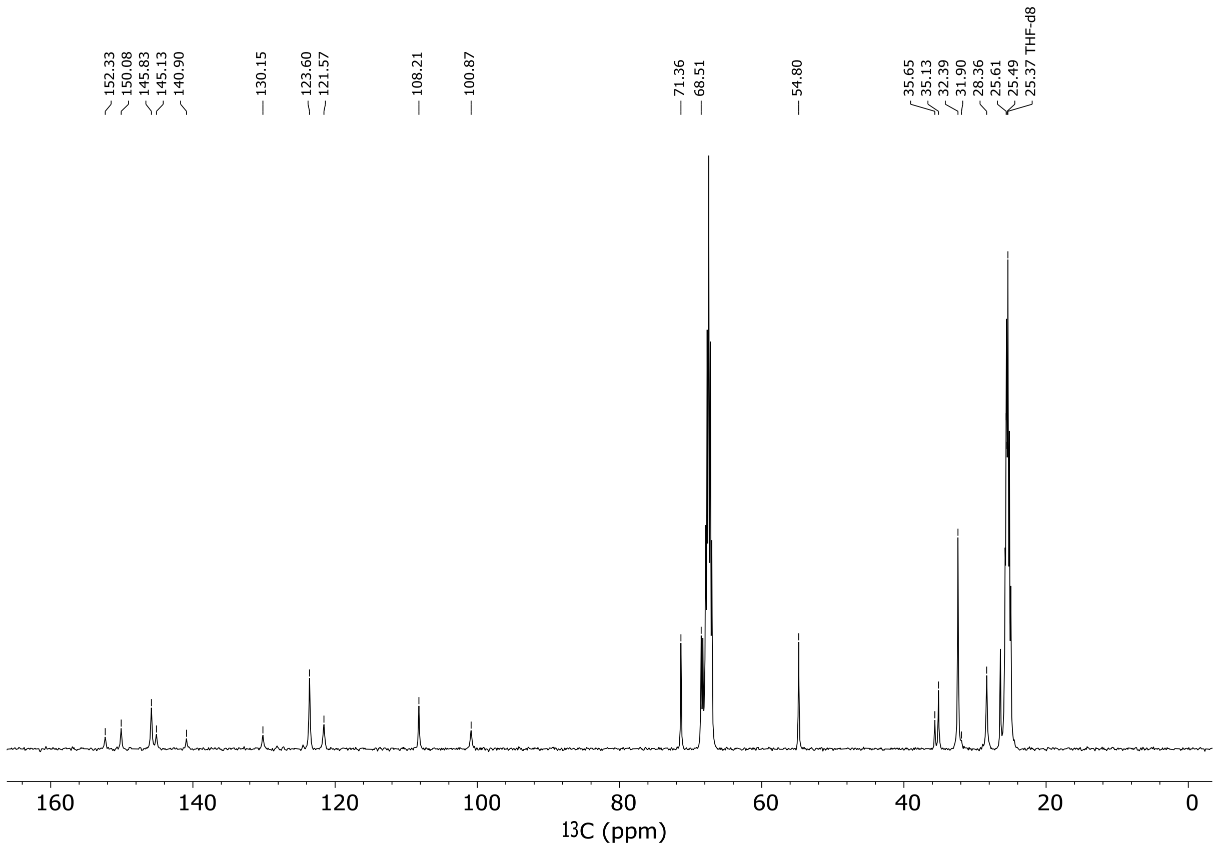

Figure S 57 *^{13}C{^{1}H} NMR spectrum of **3** in thf-d$_8$ at RT.*

4-Dipp and 4'-Dipp

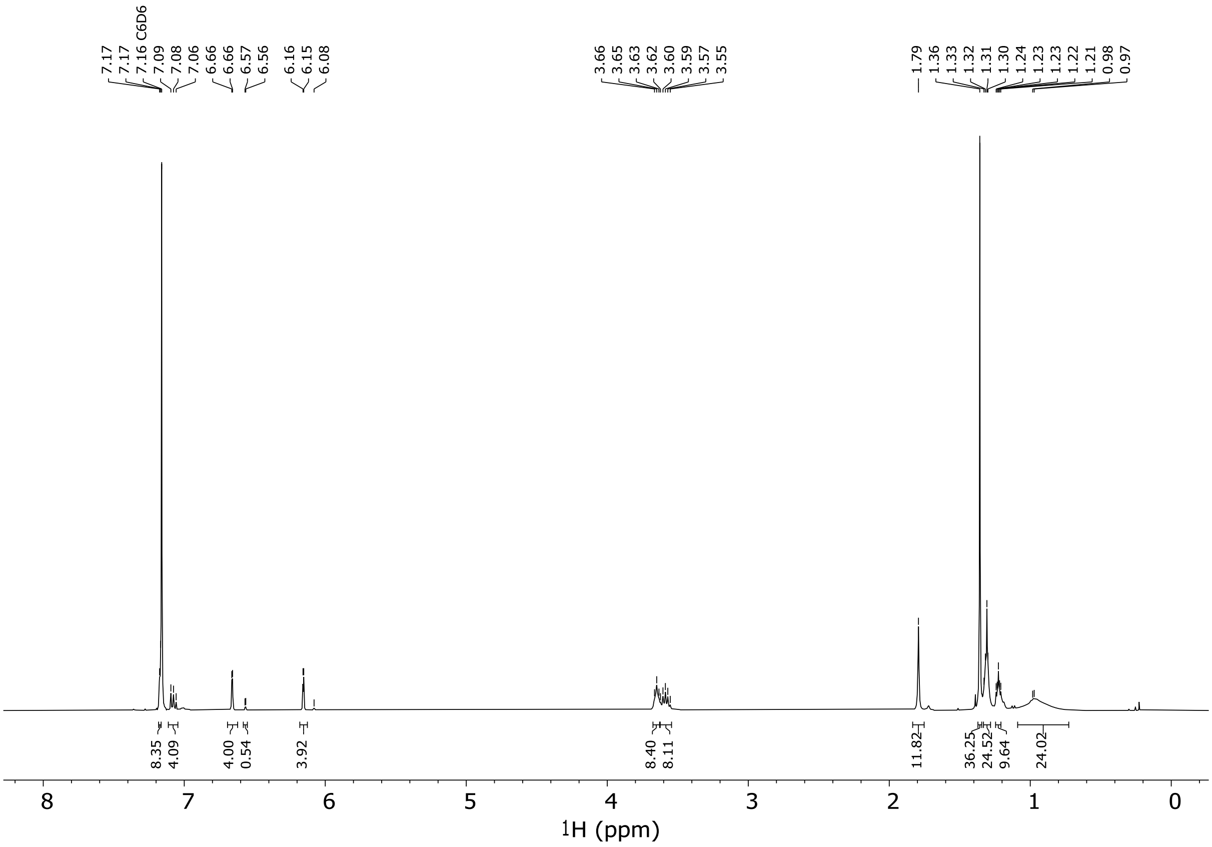

Figure S 58 *^{1}H NMR spectrum of* ***4-Dipp*** *(major) and* ***4'-Dipp*** *(minor) in C_6D_6.*

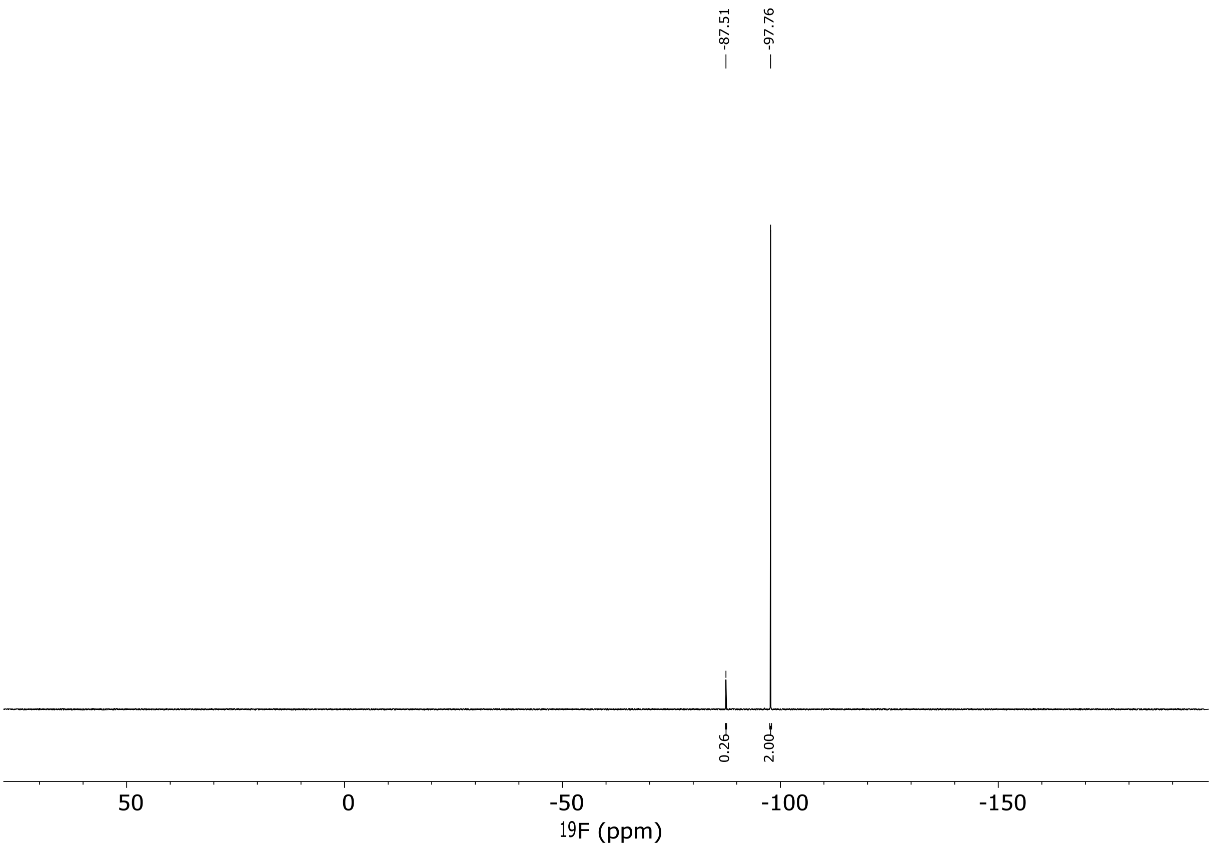

Figure S 59 *^{19}F NMR spectrum of* ***4-Dipp*** *(major) and* ***4'-Dipp*** *(minor) in C_6D_6.*

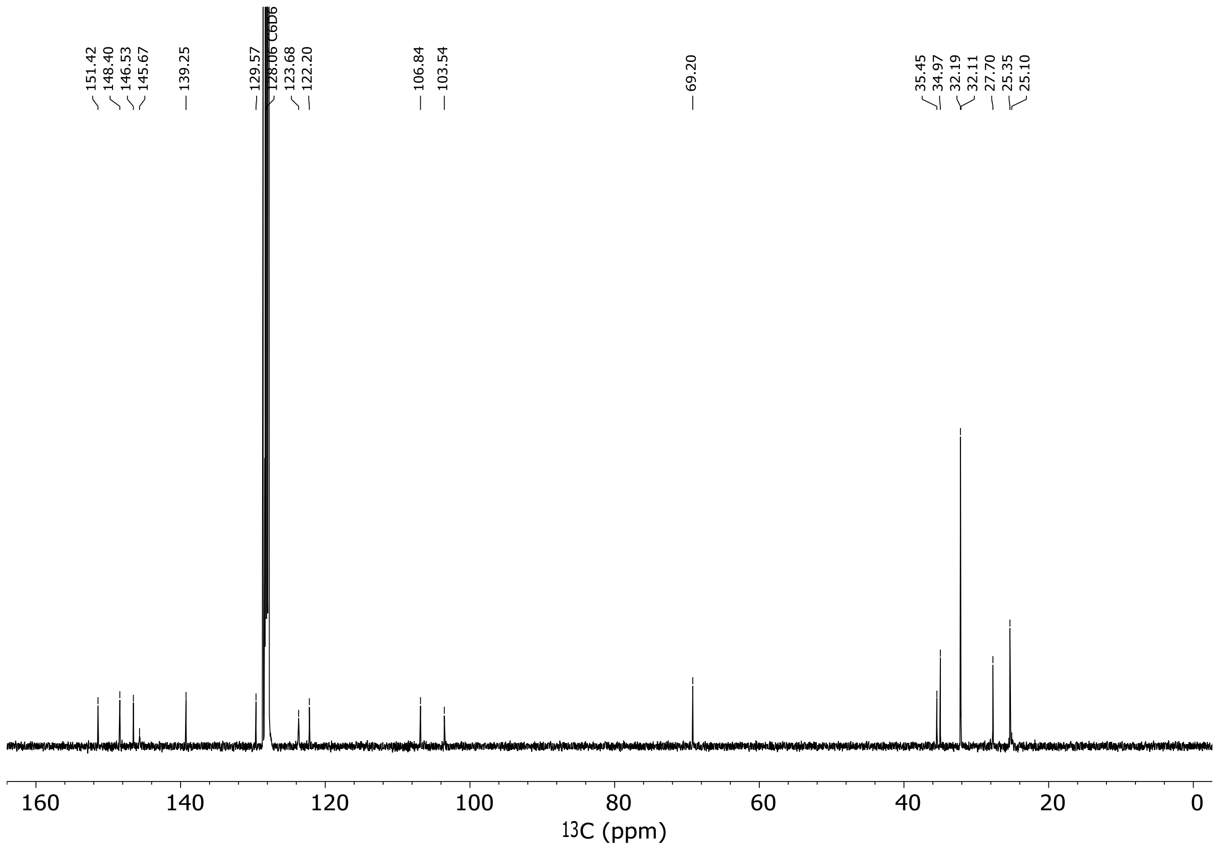

Figure S 60 *$^{13}C\{^1H\}$ NMR spectrum of* ***4-Dipp*** *(major) and* ***4’-Dipp*** *(minor) in C_6D_6.*

4’-Trip

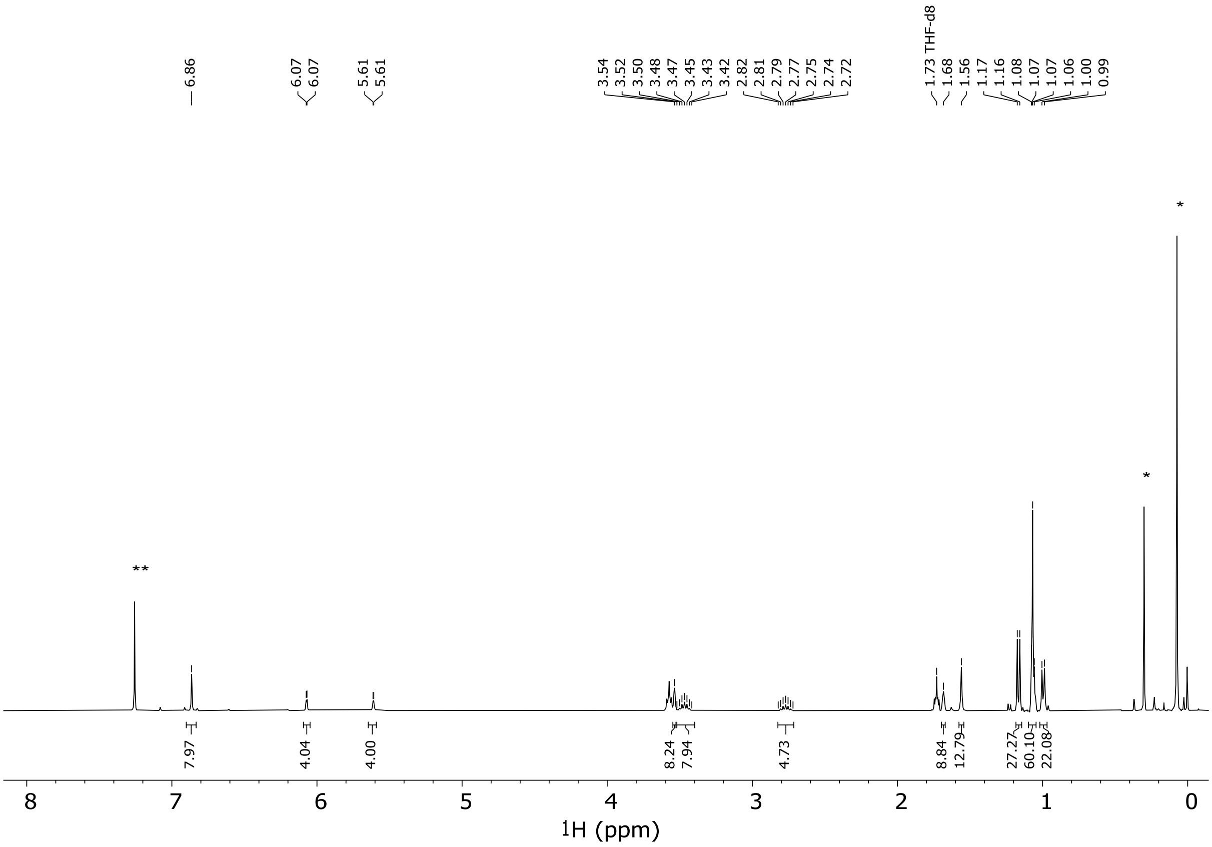

***Figure S 61** ^1H NMR spectrum of **4’**-Trip in situ after 1 day in thf-d_8. *$Me_3SnHMDS$ & **C_6H_6.*

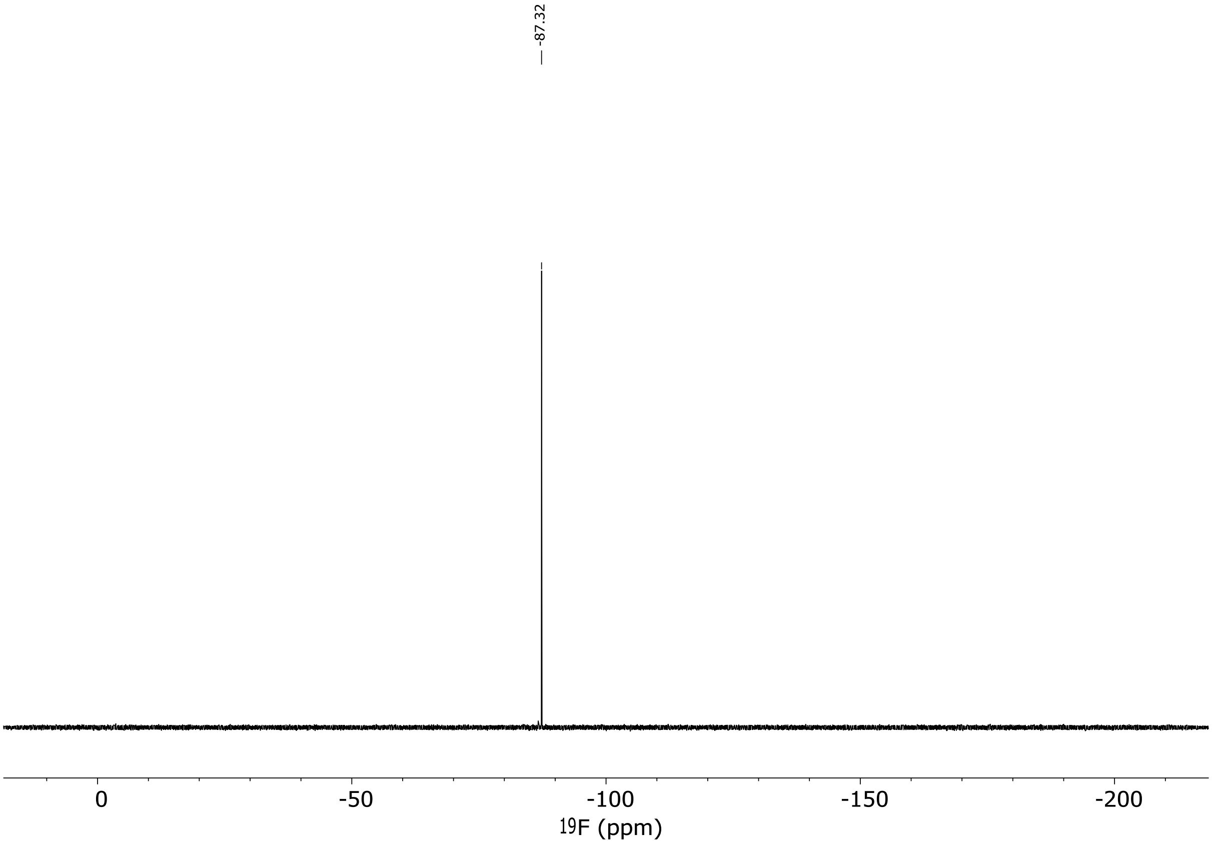

Figure S 62 *^{19}F NMR spectrum of **4’**-Trip in situ after 1 day in thf-d_8.*

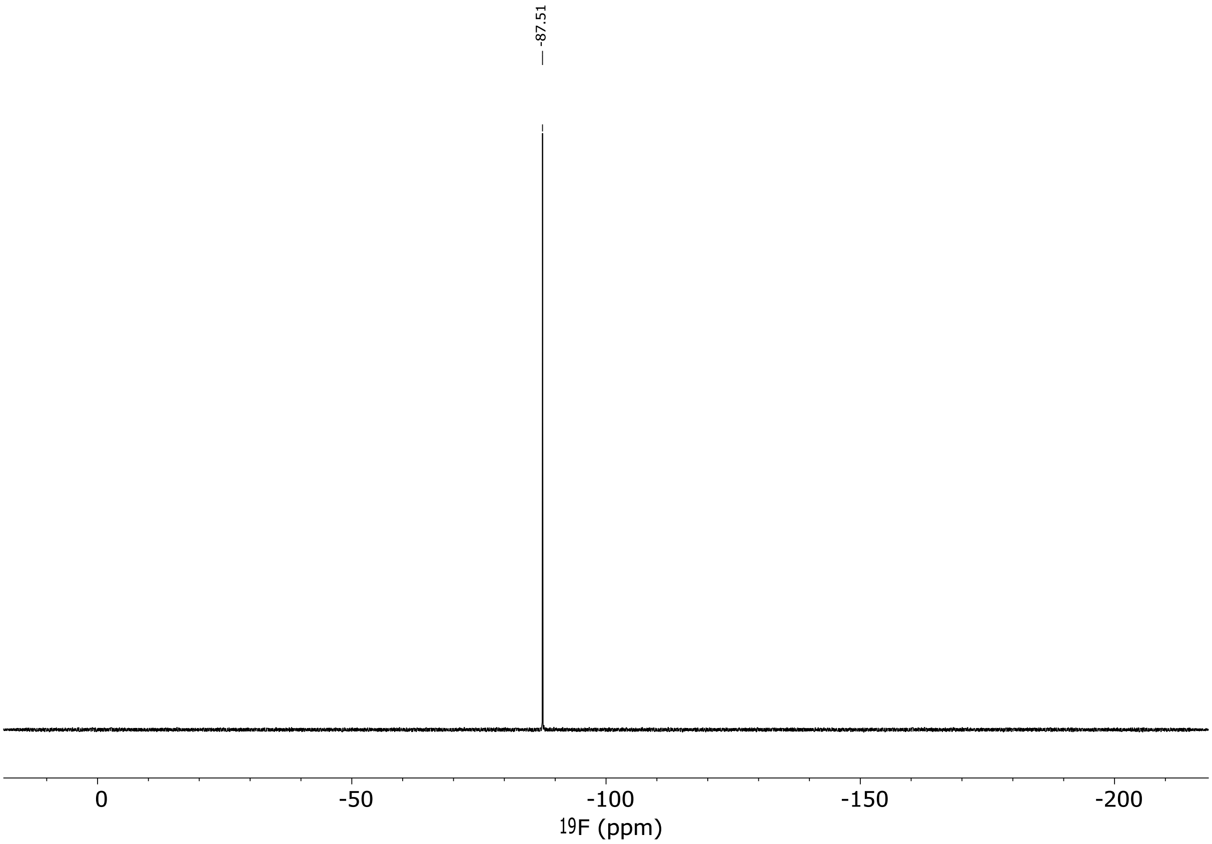

Figure S 63 *^{19}F NMR spectrum of **4'**-Trip in situ after 1 day in thf-d_8.*

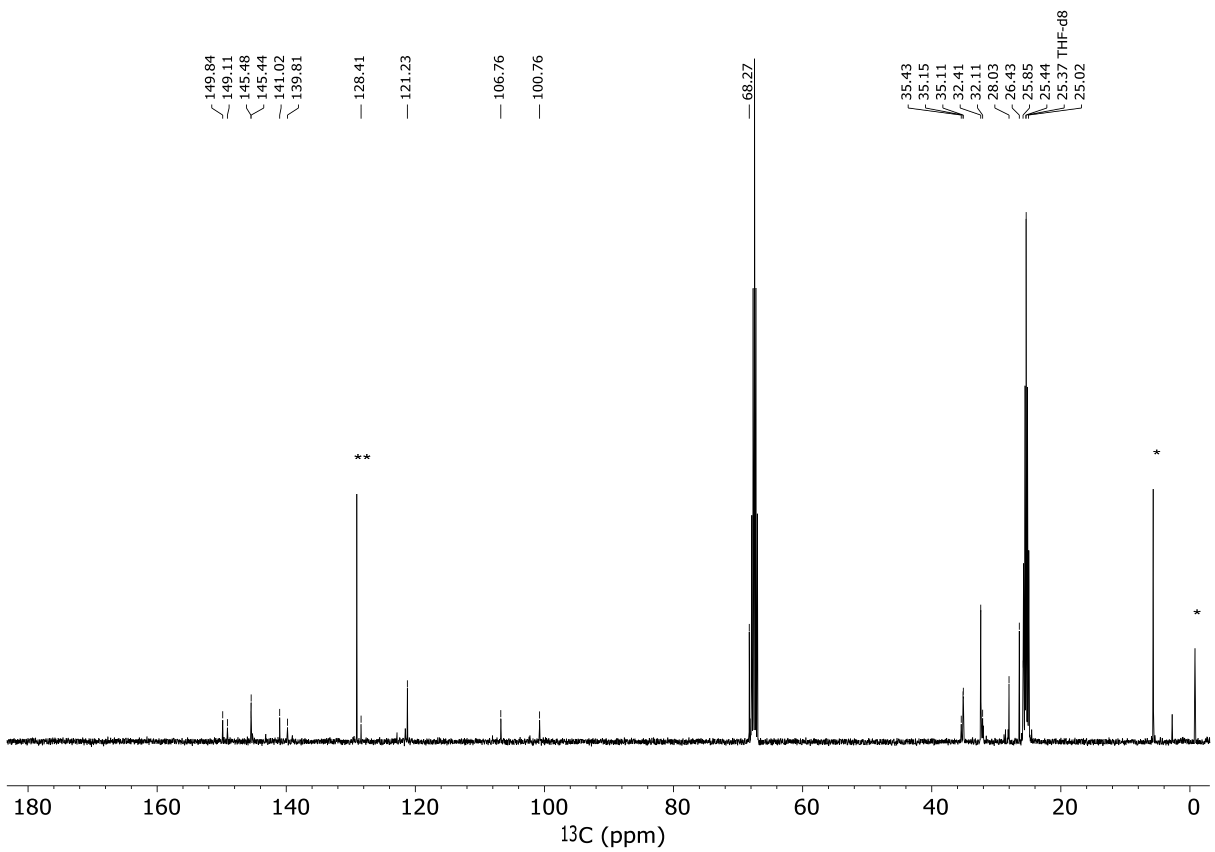

Figure S 64 *^{13}C{^{1}H} NMR spectrum of **4'**-Trip in situ after 1 day in thf-d_8. *Me_3SnHMDS & **C_6H_6.*

5

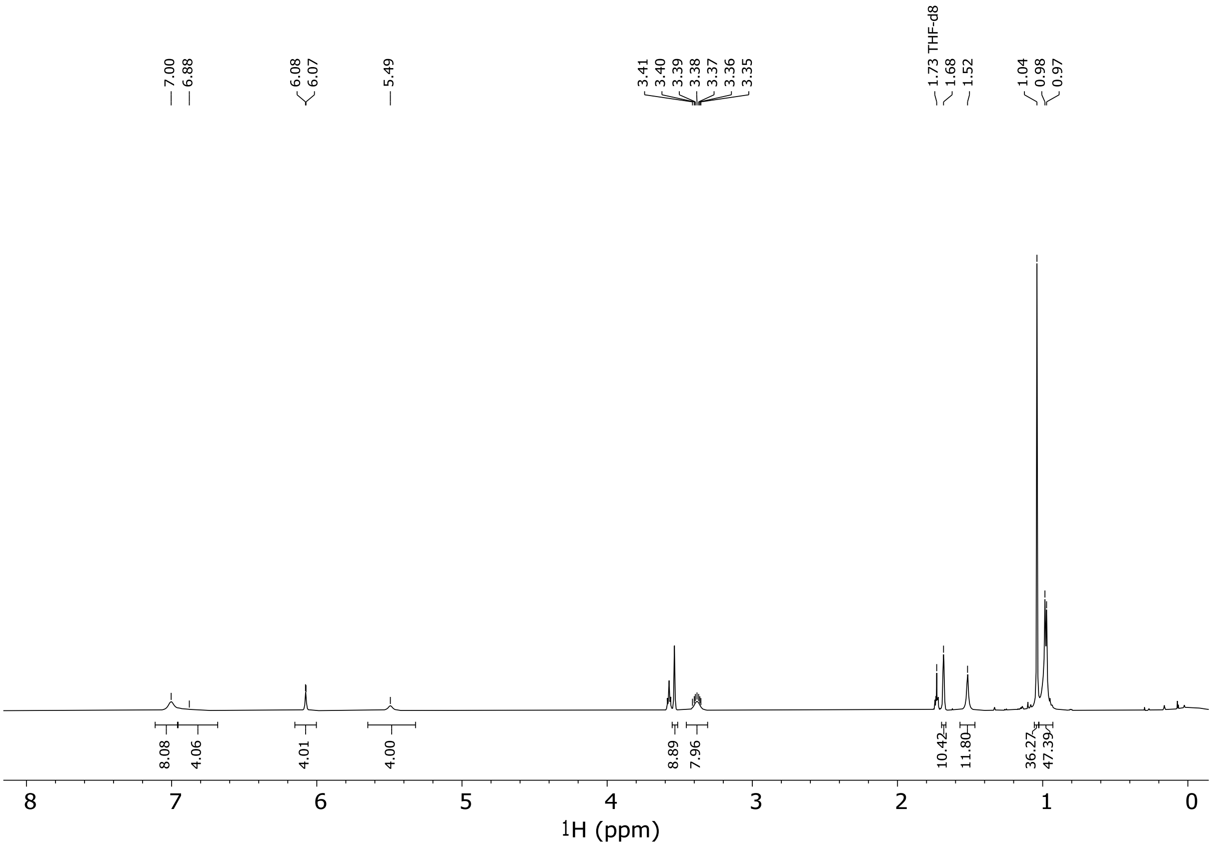

Figure S 65 *¹H NMR spectrum of* ***5*** *in thf-d₈.*

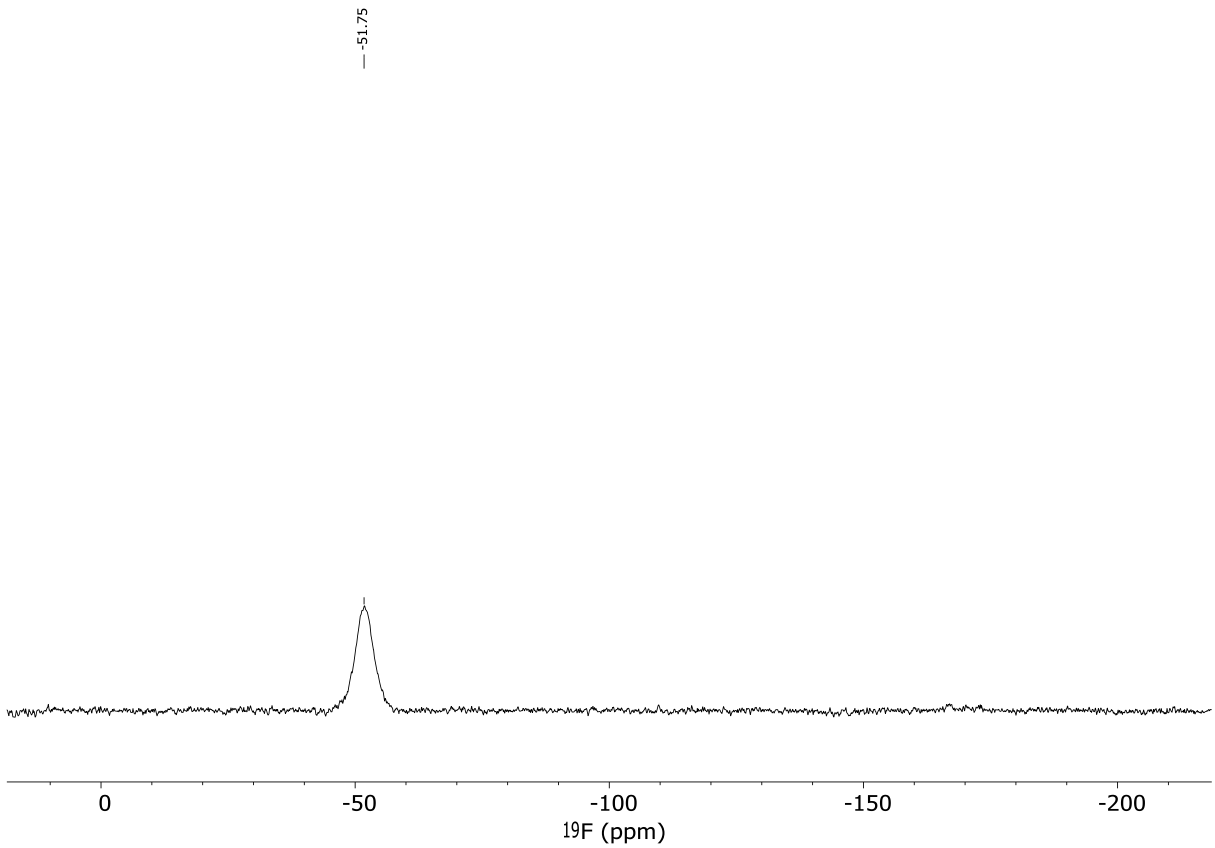

Figure S 66 *^{19}F NMR spectrum of **5** in thf-d8.*

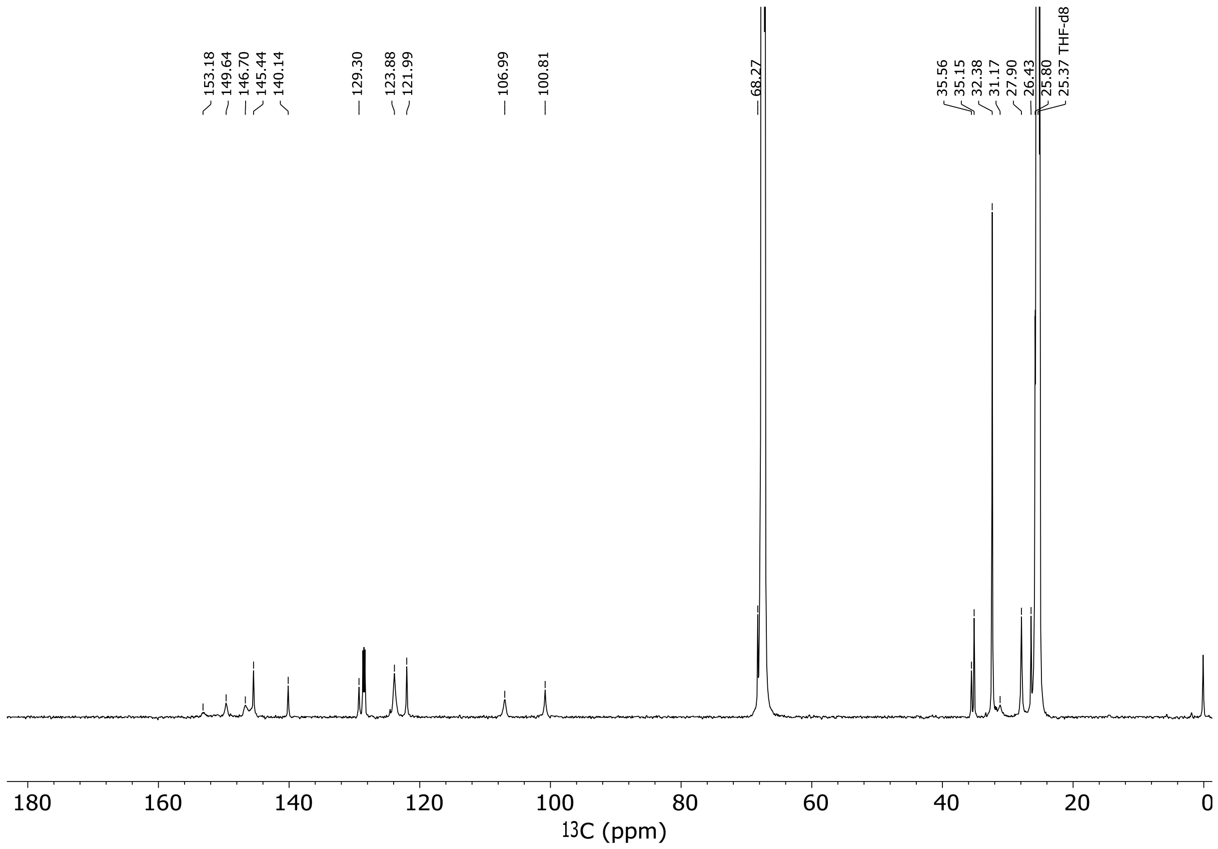

Figure S 67 *$^{13}C\{^1H\}$ NMR spectrum of **5** in thf-d8.*

6

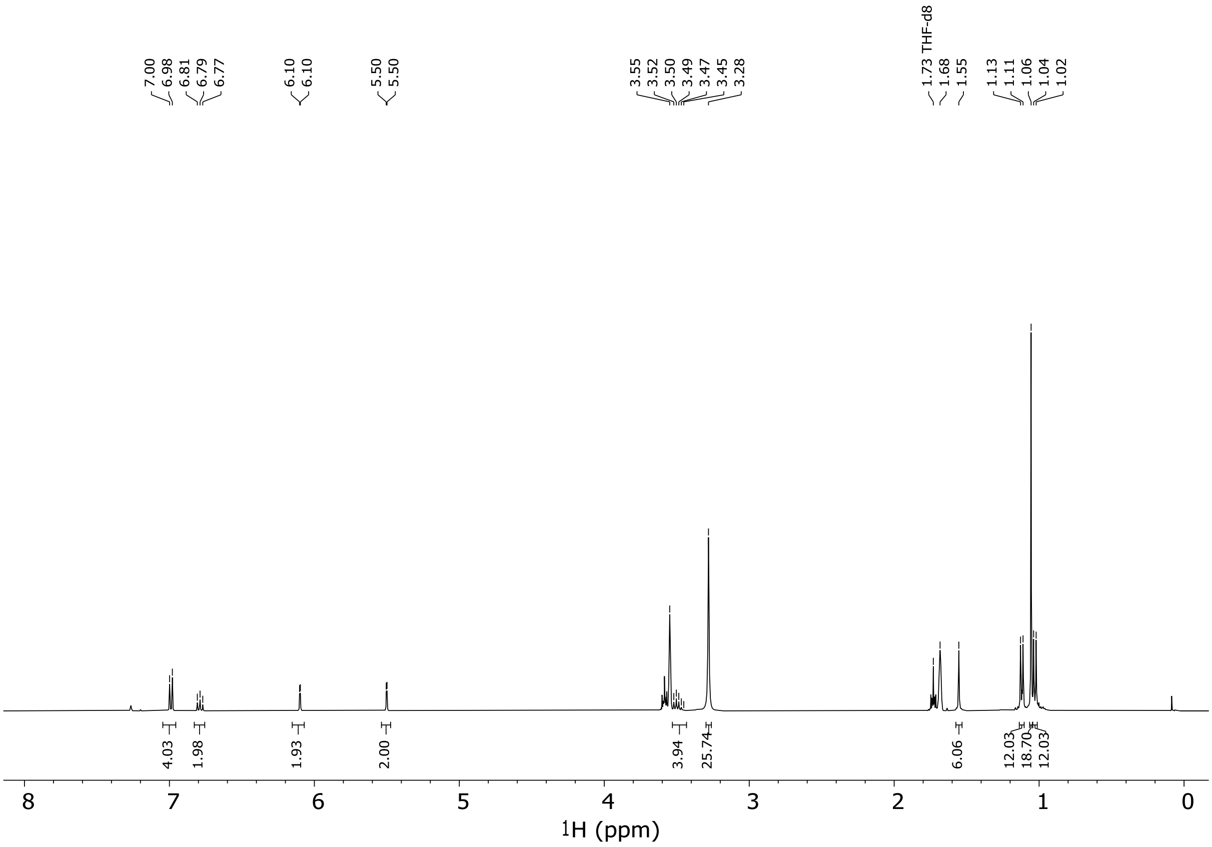

Figure S 68 *^{1}H NMR spectrum of **6** in thf-d_8.*

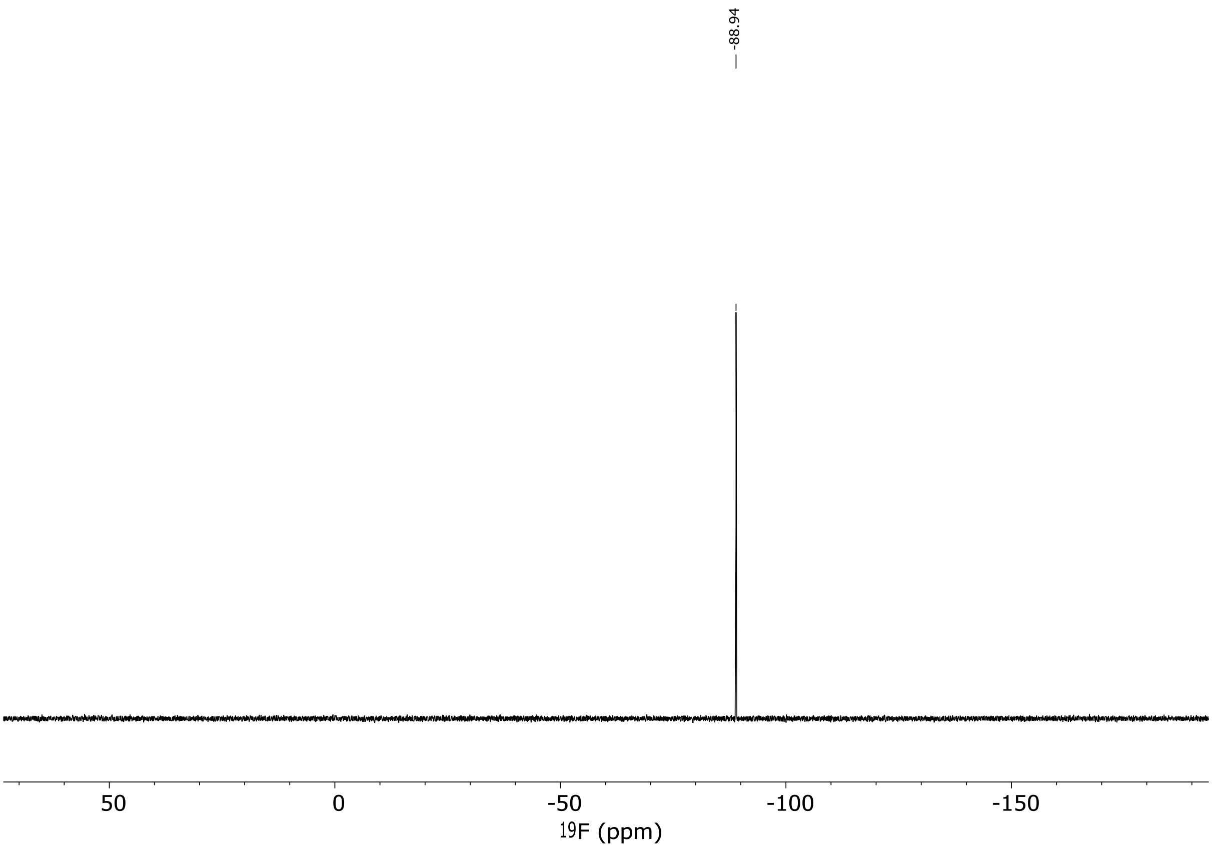

Figure S 69 *^{19}F NMR spectrum of **6** in thf-d_8.*

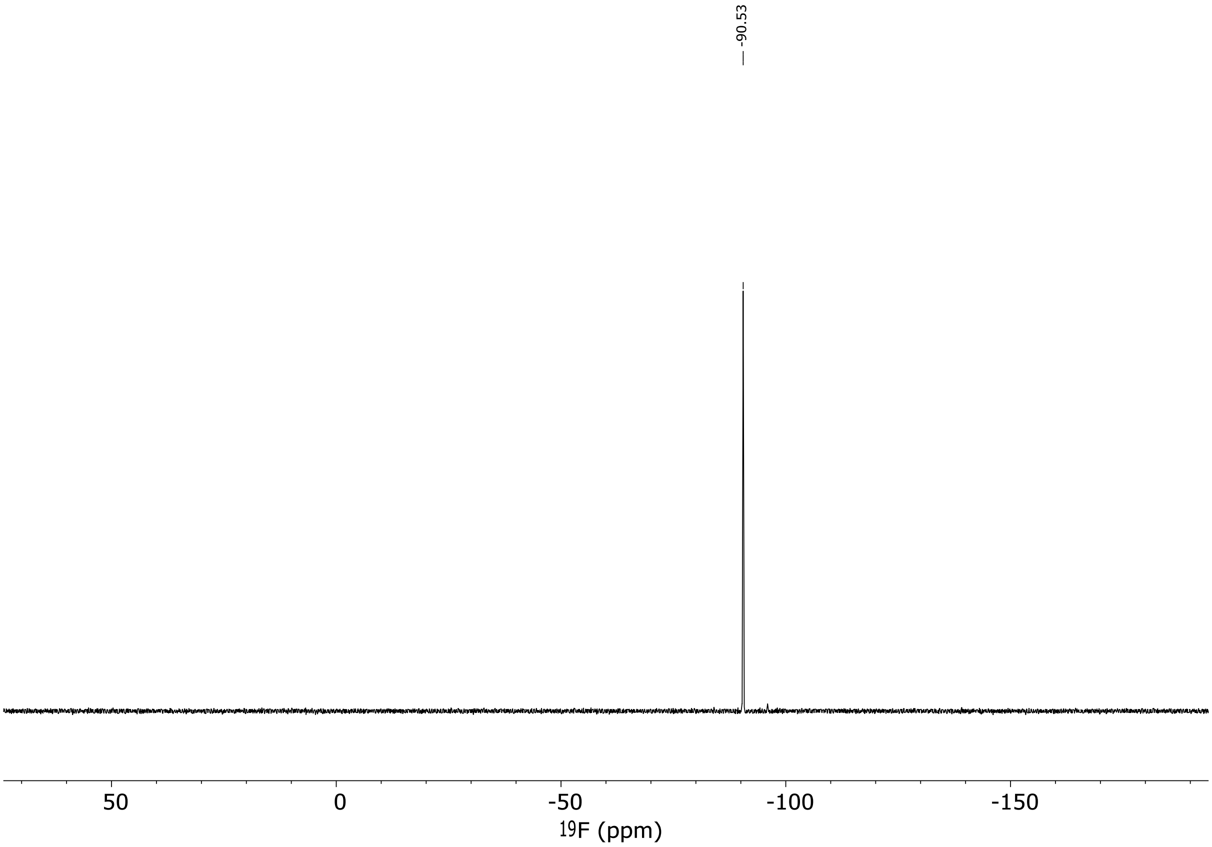

Figure S 70 ^{19}F NMR spectrum of **6** in C_6D_6.

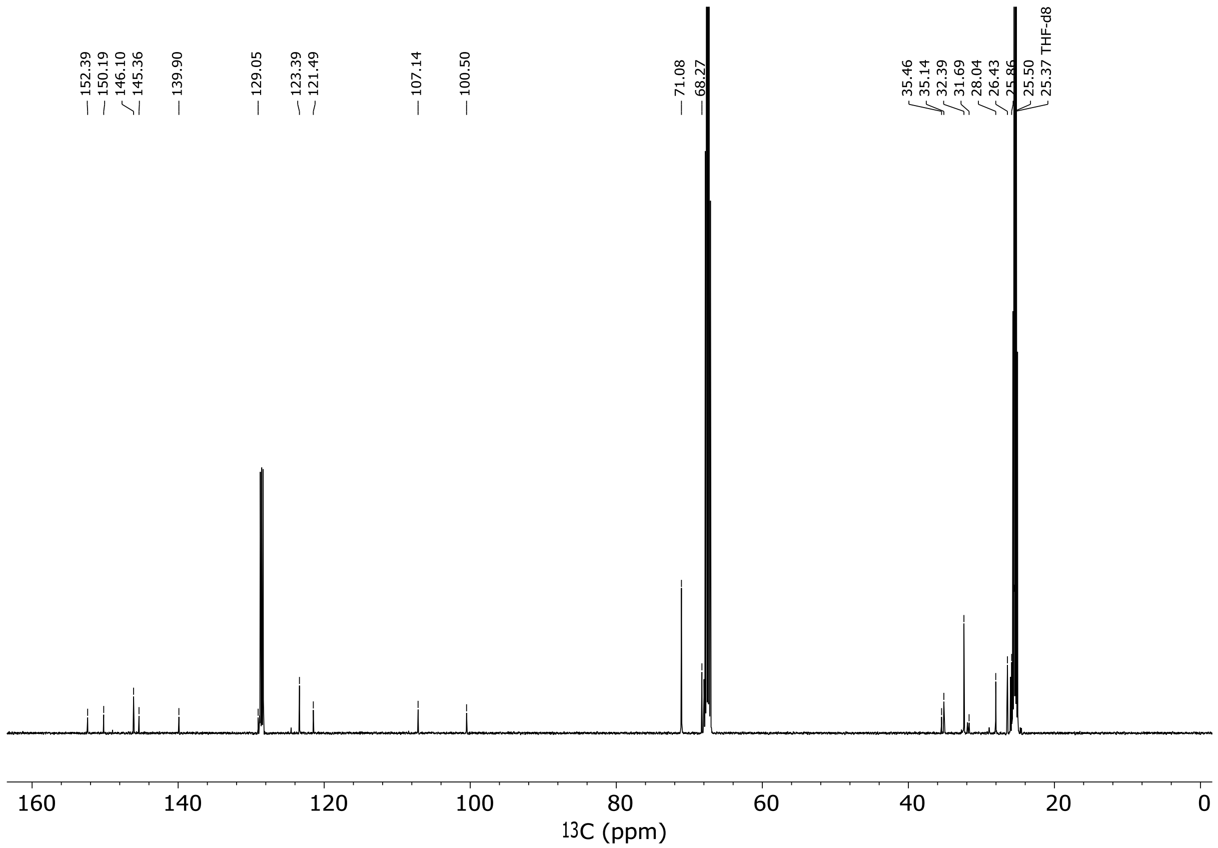

Figure S 71 *$^{13}C\{^{1}H\}$ NMR spectrum of **6** in thf-d_8.*

7

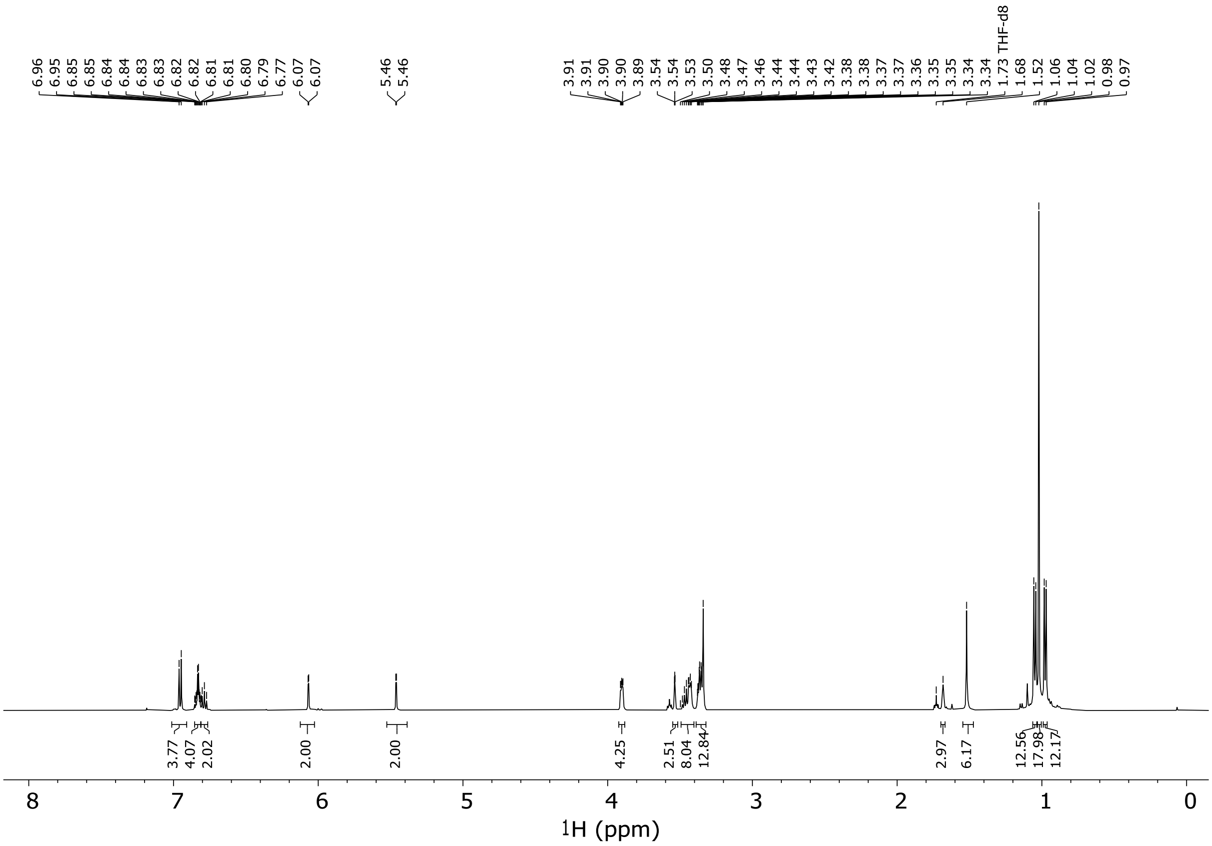

***Figure S 72** ^1H NMR spectrum of **7** in thf-d_8.*

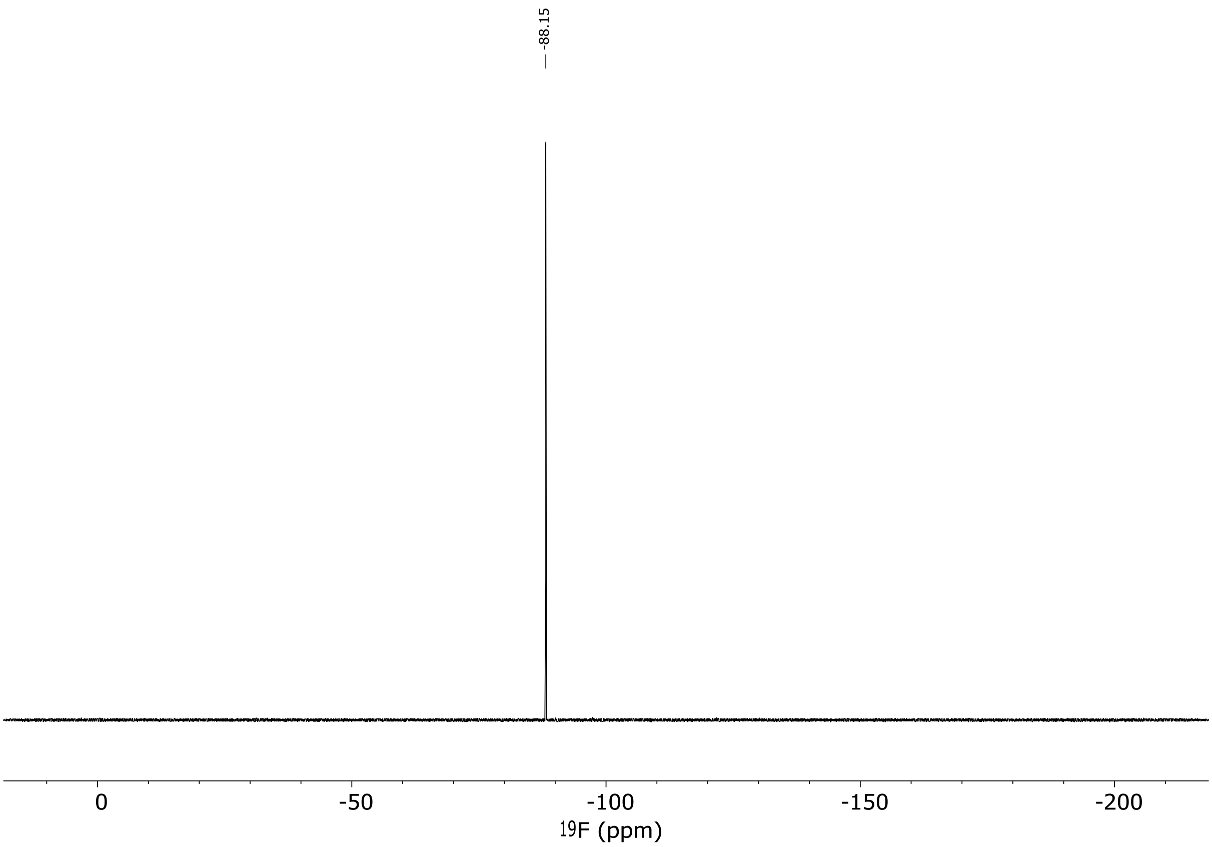

***Figure S 73** ^{19}F NMR spectrum of **7** in thf-d_8.*

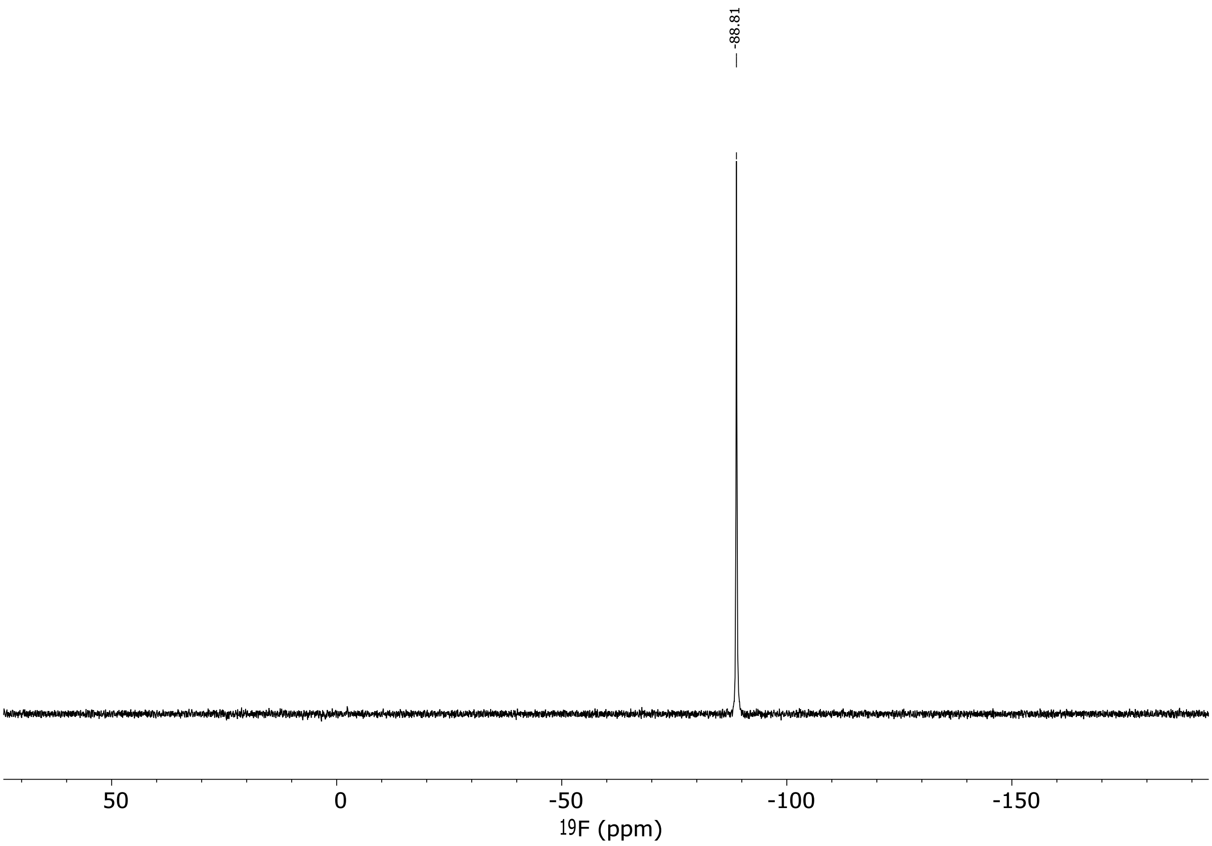

Figure S 74 *^{19}F NMR spectrum of **7** in C_6D_6.*

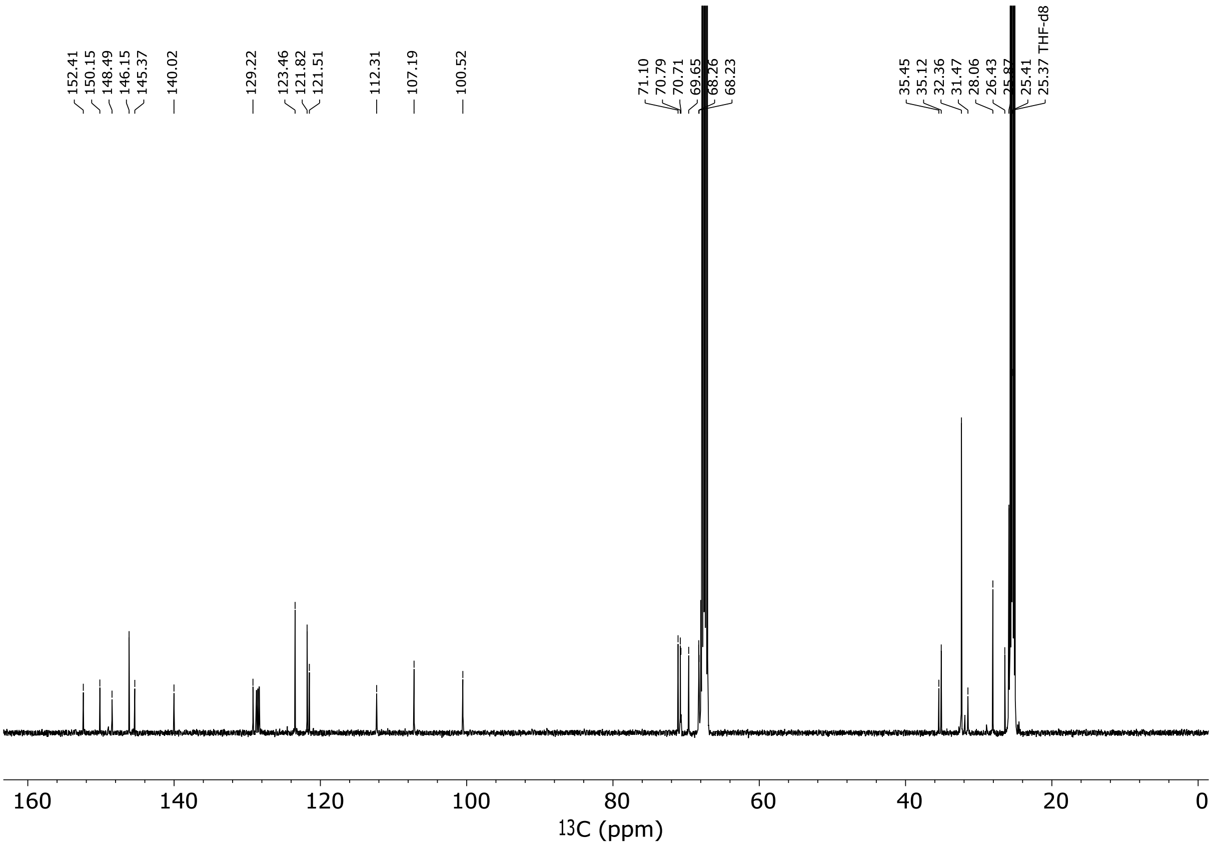

Figure S 75 *$^{13}C\{^1H\}$ NMR spectrum of **7** in thf-d_8.*

8

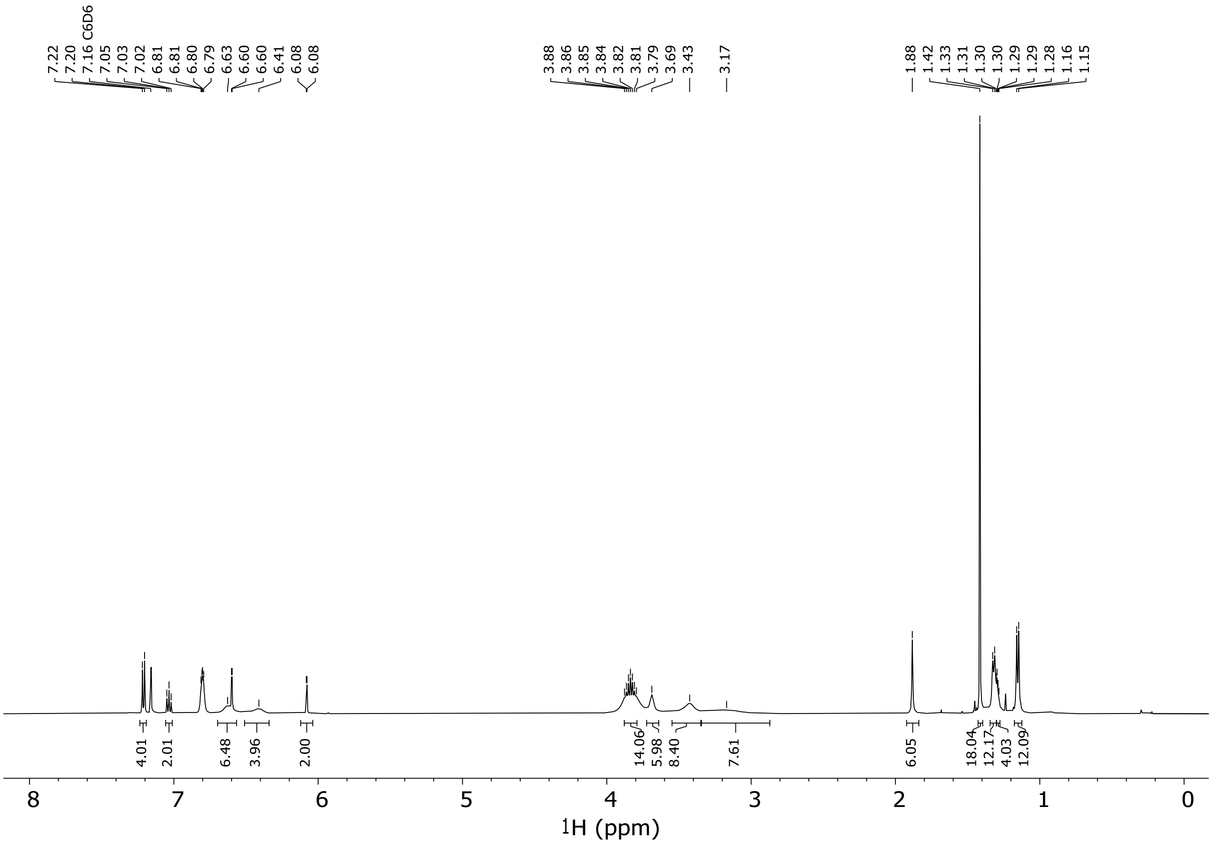

Figure S 76 *^{1}H NMR spectrum of **8** in C_6D_6. Note: an excess of dibenzo-18-crown-6 is present.*

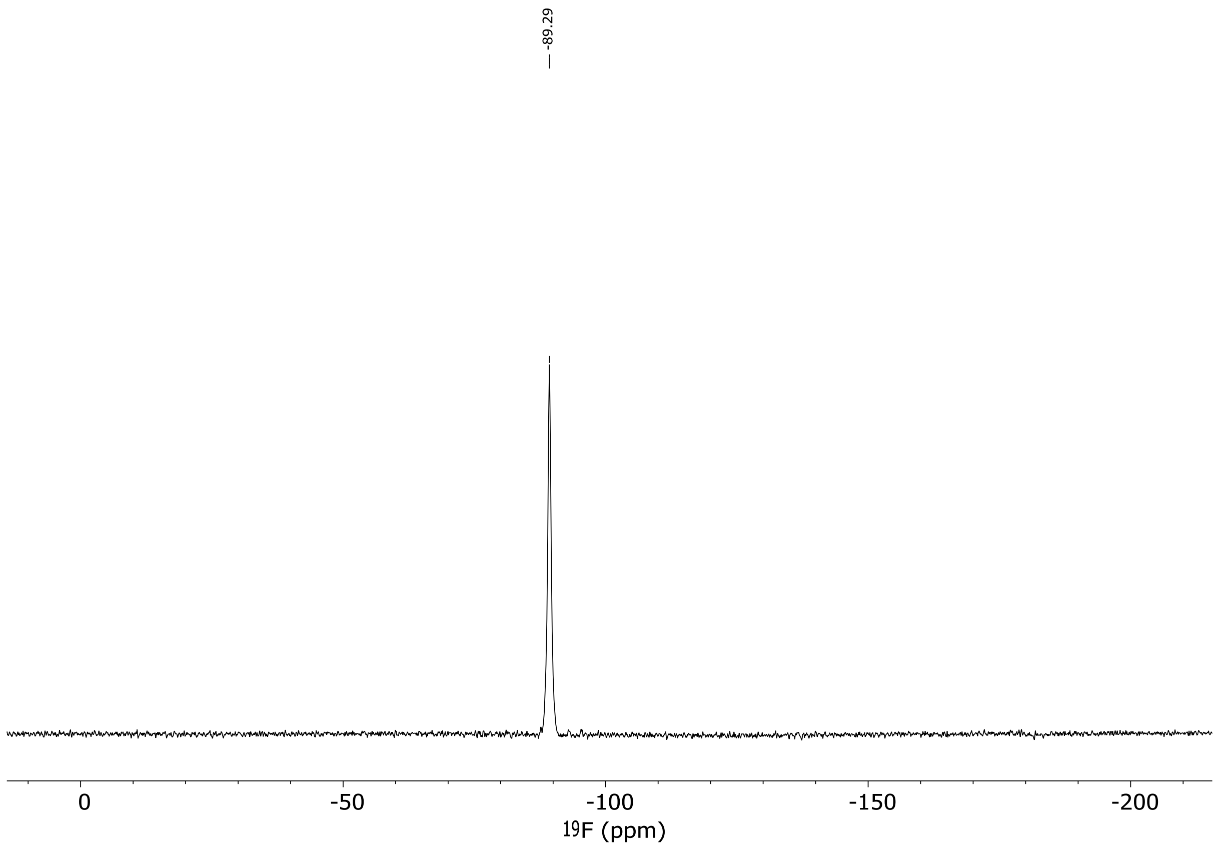

Figure S 77 *^{19}F NMR spectrum of **8** in C_6D_6.*

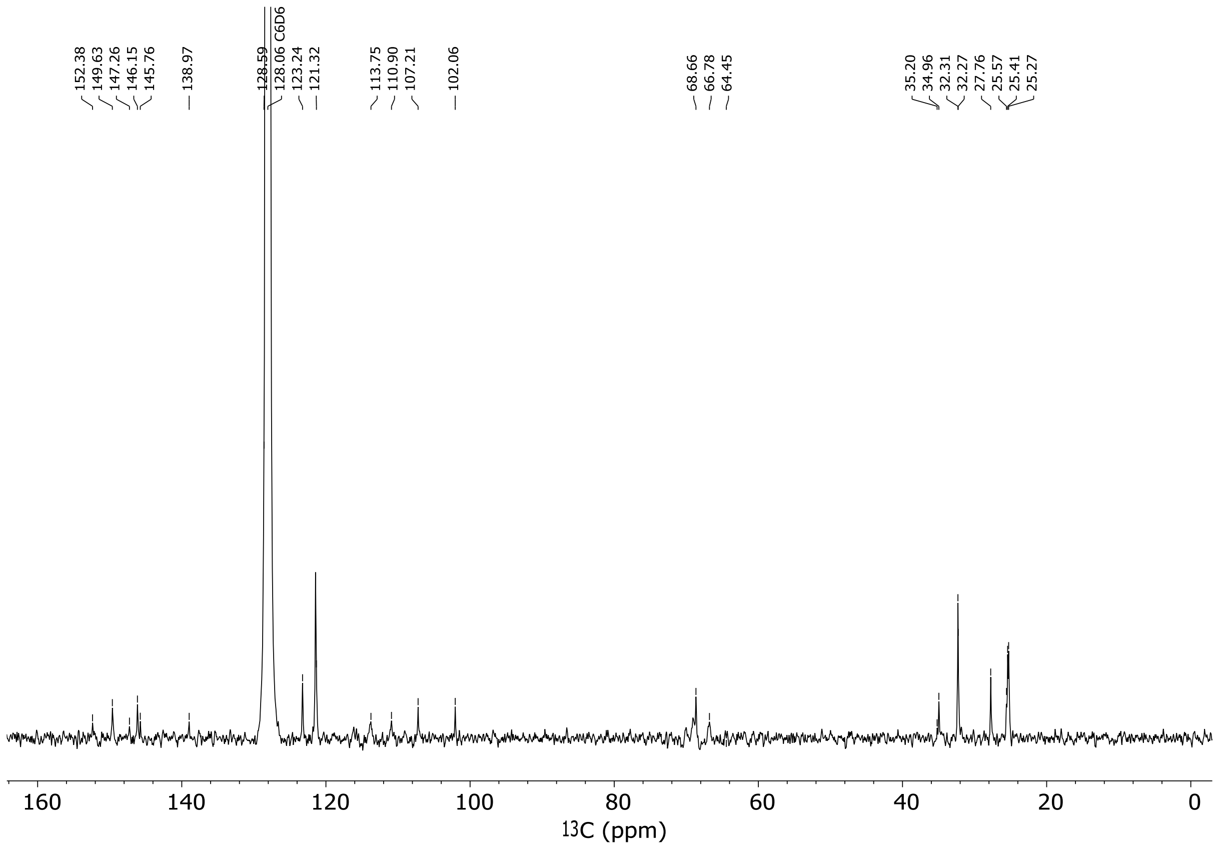

Figure S 78 *$^{13}C\{^1H\}$ NMR spectrum of **8** in C_6D_6. Note: In order to prevent crystallization of **8'** a low concentration of **8** was used resulting in the low intensity spectrum. The corresponding ^1H-^{13}C HSQC and ^1H-^{13}C HMBC are provided in the following figures.*

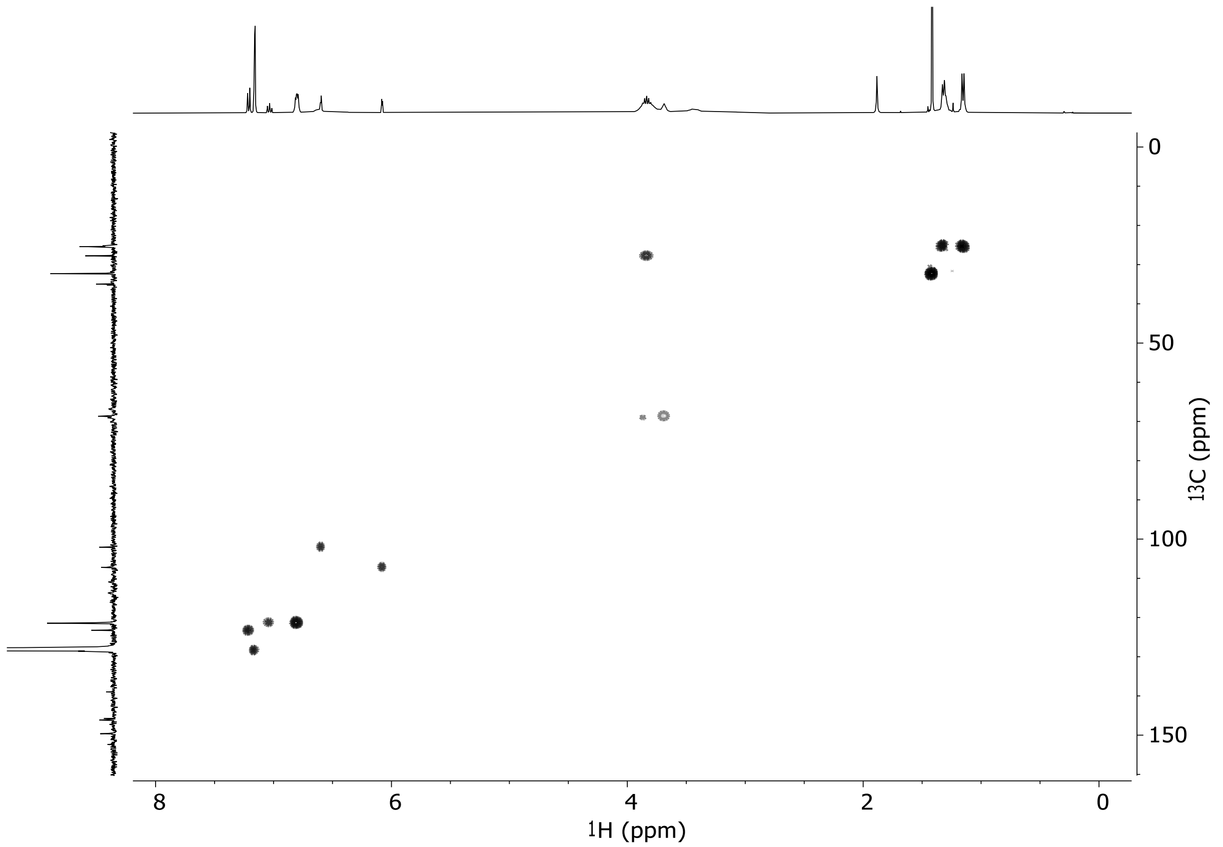

Figure S 79 *^{1}H-^{13}C HSQC NMR spectrum of **8** in C_6D_6.*

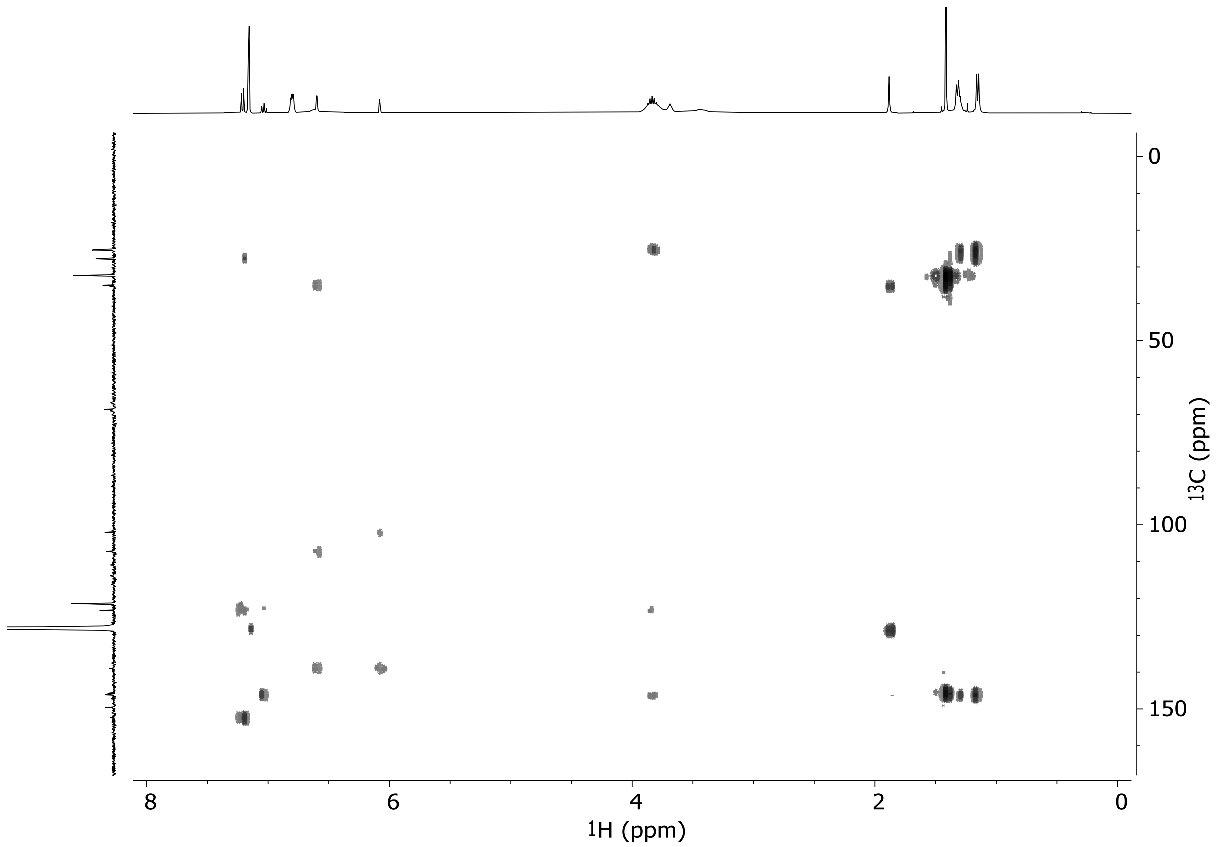

Figure S 80 *^{1}H-^{13}C HMBC NMR spectrum of **8** in C_6D_6.*

X-ray crystallographic data

***Table S 1** Crystal data and refinement details for the crystal structure analysis of **1-Dipp**, **1-Trip**.*

	**[(DippNON)Ca(thf)$_2$]**<br>**1-Dipp**	**[(TrippNON)Ca(thf)$_2$]**<br>**1-Trip**
formula	C61 H84 Ca N2 O3<br>+ C6H6	C140 H198 Ca2 N4 O6<br>+ 2 C6H6
formula weight / g·mol^{-1}	933.38	2113.17
crystal system	monoclinic	monoclinic
space group	$P2_1/c$	$P2_1$
a / Å<br>*b* / Å<br>*c* / Å	13.2262(2)<br>21.0650(3)<br>20.0381(3)	12.32450(10)<br>39.3801(4)<br>13.89760(10)
α / °<br>β / °<br>γ / °	90<br>96.246(2)<br>90	90<br>108.9800(10)<br>90
volume / Å^3<br>*Z*<br>ρ_{calc} / g·cm^{-3}	5549.67(14)<br>4<br>1.117	6378.35(10)<br>2<br>1.100
radiation (Å)	Cu Kα (1.54184)	Cu Kα (1.54184)
temperature / K	149.97(13)	100.00(10)
μ / mm^{-1}	1.304	1.186
crystal size / mm^3	0.24 x 0.158 x 0.12	0.15 x 0.13 x 0.1
F(000)	2032	2308
2θ range / °	3.963 - 76.299	3.545 - 74.706
reflection collected	54169	89088
independent reflections<br>R_{int}	11553<br>0.0531	24257<br>0.0639
data / restraints / parameters	11553 / 10 / 697	24257 / 1 / 1410
[a] goodness-of-fit on F^2	1.032	1.049
[b,c] final *R* indexes [I≥2σ (I)],<br>R_1 / ωR_2	0.0588 / 0.1523	0.0463 / 0.1112
final *R* indexes [all data]	0.0757 / 0.1701	0.0553 / 0.1151
max. peak / hole in e·Å^{-3}	0.582 / -0.286	0.396 / -0.337
CCDC reference[d]	2282778	2282783

Table S 2. *Crystal data and refinement details for the crystal structure analysis of **2**, **3** and Cs[([Dipp]NON)Ca(HMDS)]. *The refraction data for structure **2** contains a modulated component leading to poor refinement parameters (see Figure S59).*

	**[NMe$_4$]$_2$ [{(DippNON)Ca (μ$_2$-F)}$_2$]** **2***	**[K(2.2.2-crypt)] [{(DippNON)Ca(thf)}$_2$(μ$_2$-F)]** **3**	**Cs [([Dipp]NON)Ca (HMDS)]**
formula	C102 H148 Ca2 F2 N6 O2 (+0.19 Ca)	C126 H182 Ca2 F1 K1 N6 O10	C53 H80 Ca Cs N3 O Si2
formula weight / g·mol^{-1}	1615.83	2079.03	1004.37
crystal system	monoclinic	monoclinic	orthorhombic
space group	*P*n	*P*2$_1$/n	*P*2$_1$2$_1$2$_1$
a / Å<br>*b* / Å<br>*c* / Å	13.7066(1)<br>18.6723(1)<br>20.2481(2)	23.4829(1)<br>23.8922(1)<br>24.0078(1)	15.3681(1)<br>7.9451(1)<br>19.4930(1)
α / °<br>β / °<br>γ / °	90<br>104.814(1)<br>90	90<br>105.149(1)<br>90	90<br>90<br>90
volume / Å^3<br>*Z*<br>ρ_{calc} / g·cm^{-3}	5009.9(1)<br>2<br>1.071	13001.7(1)<br>4<br>1.062	5375.8(1)<br>4<br>1.241
radiation (Å)	Cu Kα (1.54184)	Cu Kα (1.54184)	Cu Kα (1.54184)
temperature / K	100.1(3)	150.0(1)	100.0(1)
μ / mm^{-1}	1.462	1.477	6.911
crystal size / mm^3	0.08 x 0.08 x 0.10	0.338 x 0.239 x 0.245	0.06 x 0.06 x 0.08
F(000)	1759	4512	2120
2θ range / °	3.271 - 74.794	2.986 - 76.234	3.348 - 74.747
reflection collected	19863	114535	66842
independent reflections<br>R_{int}	18754<br>0.0568	26937<br>0.0326	10571<br>0.0325
data / restraints / parameters	18754 / 24 / 1106	26937 / 53 / 1319	10571 / 0 / 572
[a] goodness-of-fit on F^2	1.434	1.047	1.047
[b,c] final *R* indexes [I≥2σ (I)], R_1 / ωR_2	0.1097 / 0.2935	0.0513 / 0.1438	0.0232 / 0.0597
final *R* indexes [all data]	0.1120 / 0.2976	0.0619 / 0.1536	0.0244 / 0.0601
max. peak / hole in e·Å^{-3}	2.259 / -0.564	0.997 / -0.608	1.07 / -0.56
CCDC reference[d]	2283075	2282786	2282803

[a] $S = \{\sum[\omega(F_o^2-F_c^2)^2]/(n-p)\}^{0.5}$; n = no. of reflections; p = no. of parameters. [b] $R_1 = \sum||F_0|-|F_c||/\sum|F_0|$. [c] $\omega R_2 = \{\sum[\omega(F_o^2-F_c^2)^2]/\sum[\omega(F_o^2)^2]\}^{0.5}$. [d] data are available free of charge via "www.ccdc.cam.ac.uk/data_request/cif".

Table S 2. *Crystal data and refinement details for the crystal structure analysis of **4-Dipp**, **4-Trip** and **5**.*

	**[K(DippNON)CaF(thf)]₂** **4-Dipp**	**[K(TripNON)CaF(thf)]₂** **4-Trip**	**[Cs(DippNON)CaF(thf)]₂** **5**
formula	C102 H140 Ca2 F2 K2 N4 O4 + 6 C6H6	C126 H176 Ca2 F2 K2 N4 O4 + 2 C6H6	C102 H140 Ca2 Cs2 F2 N4 O4 + 6 C6H6
formula weight / g·mol^{-1}	2151.18	2007.06	2338.81
crystal system	monoclinic	triclinic	monoclinic
space group	$P2_1/c$	$P\bar{1}$	$P2_1/c$
a / Å<br>*b* / Å<br>*c* / Å	13.9551(2)<br>23.3258(3)<br>20.5937(3)	12.2985(2)<br>14.3872(2)<br>17.7242(2)	13.9207(3)<br>23.1542(3)<br>20.6149(3)
α / °<br>β / °<br>γ / °	90<br>109.556(2)<br>90	72.094(1)<br>78.749(1)<br>75.919(1)	90<br>109.306(2)<br>90
volume / Å^3<br>*Z*<br>ρ_{calc} / g·cm^{-3}	6316.8(2)<br>2<br>1.131	2869.9(1)<br>2<br>1.161	6271.0(2)<br>2<br>1.239
radiation (Å)	Cu Kα (1.54184)	Cu Kα (1.54184)	Mo Kα (0.71073)
temperature / K	150.0(1)	100.0(1)	100.0(1)
μ / mm^{-1}	1.799	1.942	0.719
crystal size / mm^3	0.462 x 0.272 x 0.179	0.278 x 0.164 x 0.138	0.09 x 0.11 x 0.12
F(000)	2320	1088	2464
2θ range / °	3.790 - 76.293	3.292 - 77.011	1.7510 - 43.7980
reflection collected	48950	165308	68920
independent reflections<br>R_{int}	12972<br>0.0306	11738<br>0.1159	14367<br>0.0582
data / restraints / parameters	12972 / 579 / 875	11738 / 249 / 755	14367 / 1631 / 924
[a] goodness-of-fit on F^2	1.019	1.065	1.085
[b,c] final *R* indexes [I≥2σ (I)], R_1 / ωR_2	0.0426 / 0.1170	0.0576 / 0.1545	0.0578 / 0.1174
final *R* indexes [all data]	0.0529 / 0.1271	0.0799 / 0.1745	0.0748 / 0.1267
max. peak / hole in e·Å^{-3}	0.314 / -0.313	0.672 / -0.642	1.079 / -0.838
CCDC reference[d]	2282794	2282801	2282807

[a] $S = \{\sum[\omega(F_o^2-F_c^2)^2]/(n-p)\}^{0.5}$; n = no. of reflections; p = no. of parameters. [b] $R_1 = \sum||F_0|-|F_c||/\sum|F_0|$. [c] $\omega R_2 = \{\sum[\omega(F_o^2-F_c^2)^2]/\sum[\omega(F_o^2)^2]\}^{0.5}$. [d] data are available free of charge via "www.ccdc.cam.ac.uk/data_request/cif".

Table S 2. *Crystal data and refinement details for the crystal structure analysis of* ***6****,* ***7****, and* ***8'****.*

	**[(K 18-crown-6) (DippNON)CaF(thf)]** **6**	**[(K benzo-18-crown-6) (DippNON)CaF(thf)]** **7**	**[(K dibenzo-18-crown-6) K(DippNON)CaF$_2$]$_2$** **8'**
formula	C63 H94 Ca F K N2 O8	C67 H94 Ca F K N2 O8 + 2 C6H6	C134 H172 Ca2 F4 K4 N4 O14
formula weight / g·mol^{-1}	1105.59	1309.84	2375.31
crystal system	monoclinic	triclinic	monoclinic
space group	$P2_1/n$	$P\bar{1}$	$P2_1/n$
a / Å<br>*b* / Å<br>*c* / Å	24.7501(3)<br>12.0012(1)<br>27.4877(3)	16.8088(2)<br>20.5830(5)<br>22.8246(5)	18.376(2)<br>21.2033(14)<br>20.191(3)
α / °<br>β / °<br>γ / °	90<br>114.810(1)<br>90	81.403(2)<br>73.978(2)<br>79.128(2)	90<br>101.146(12)<br>90
volume / Å^3<br>*Z*<br>ρ_{calc} / g·cm^{-3}	7411.1(2)<br>4<br>0.991	7414.0(3)<br>4<br>1.173	7718.7(14)<br>2<br>1.022
radiation (Å)	Cu Kα (1.54184)	Cu Kα (1.54184)	Cu Kα (1.54184)
temperature / K	200.0(1)	100.0(1)	150.0(1)
μ / mm^{-1}	1.603	1.680	2.055
crystal size / mm^3	0.12 x 0.05 x 0.04	0.19 x 0.41 x 0.44	0.274 x 0.098 x 0.072
F(000)	2392	2824	2536
2θ range / °	2.020 - 75.897	3.260 - 74.171	3.636 - 76.750
reflection collected	15108	212945	59469
independent reflections<br>R_{int}	10592<br>0.0438	30160<br>0.0748	15857<br>0.0966
data / restraints / parameters	10592 / 783 / 971	30160 / 2080 / 2023	15857 / 2 / 763
[a] goodness-of-fit on F^2	1.070	1.026	0.968
[b,c] final *R* indexes [I≥2σ (I)], R_1 / ωR_2	0.0592 / 0.2014	0.0516 / 0.1396	0.0717 / 0.1898
final *R* indexes [all data]	0.0782 / 0.1858	0.0614 / 0.1396	0.1423 / 0.2392
max. peak / hole in e·Å^{-3}	0.216 / -0.363	0.775 / -0.566	0.322 / -0.323
CCDC reference[d]	2283071	2282808	2282811

[a] $S = \{\sum[\omega(F_o^2-F_c^2)^2]/(n-p)\}^{0.5}$; n = no. of reflections; p = no. of parameters. [b] $R_1 = \sum||F_0|-|F_c||/\sum|F_0|$. [c] $\omega R_2 = \{\sum[\omega(F_o^2-F_c^2)^2]/\sum[\omega(F_o^2)^2]\}^{0.5}$. [d] data are available free of charge via "www.ccdc.cam.ac.uk/data_request/cif".

Table S 2. *Crystal data and refinement details for the crystal structure analysis of **9**.*

	**$[K(DippNON)CaH(thf)]_2$**<br>**9**
formula	C102 H142 Ca2 K2 N4 O4 + 6 C6H6
formula weight / g·mol^{-1}	2115.19
crystal system	monoclinic
space group	$P2_1/n$
a / Å<br>b / Å<br>c / Å	13.9341(1)<br>23.1953(1)<br>20.6154(2)
α / °<br>β / °<br>γ / °	90<br>109.653(1)<br>90
volume / Å^3<br>Z<br>ρ_{calc} / g·cm^{-3}	6274.9(1)<br>2<br>1.120
radiation (Å)	Cu Kα (1.54184)
temperature / K	100.0(1)
μ / mm^{-1}	1.780
crystal size / mm^3	0.12 x 0.05 x 0.02
F(000)	2288
2θ range / °	2.944 - 75.767
reflection collected	65207
independent reflections<br>R_{int}	12943<br>0.0456
data / restraints / parameters	12943 / 471 / 820
[a] goodness-of-fit on F^2	1.089
[b,c] final R indexes [I≥2σ (I)],<br>R_1 / ωR_2	0.0414 / 0.1165
final R indexes [all data]	0.0446 / 0.1183
max. peak / hole in e·Å^{-3}	0.486 / -0.534
CCDC reference[d]	2312195

B alerts

$(^{Dipp}NON)H_2\cdot IMe_4$

- PLAT097_ALERT_2_B

PROBLEM: Large Reported Max. (Positive) Residual Density 0.82 eA-3

RESPONSE: lone pair of IME4 pointed towards protons of amine.

$[NMe_4]_2[\{(DippNON)Ca(\mu_2\text{-}F)\}_2]$ (**2**)

- PLAT094_ALERT_2_B

PROBLEM: Ratio of Maximum / Minimum Residual Density .... 4.01 Report

RESPONSE: The residual electron density is a function of the modulation that we can see in the structure. This has been partially modeled by the inclusion of a second set of Ca atoms, but this does not fully account for the residual density.

- PLAT097_ALERT_2_B

PROBLEM: Large Reported Max. (Positive) Residual Density 2.26 eA-3

RESPONSE: The residual electron density is a function of the modulation that we can see in the structure. This has been partially modeled by the inclusion of a second set of Ca atoms, but this does not fully account for the residual density.

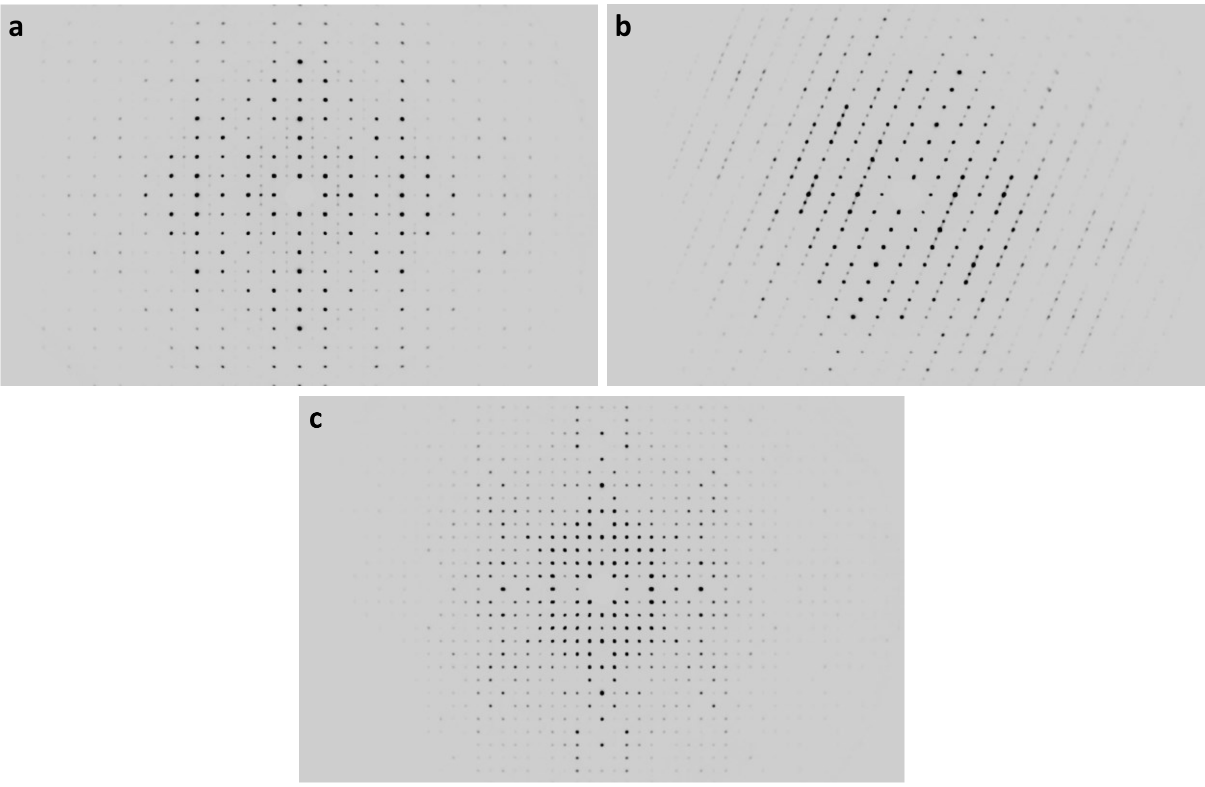

***Figure S 81** Unwarp images for the data of structure **2**, viewed in the directions (0 k l) (a), (h 0 l) (b), and (h k 0) (c), showing the modulated contributions.*

References

1. Cruz, C. A., Emslie, D. J. H., Harrington, L. E., Britten, J. F. & Robertson, C. M. Extremely Stable Thorium(IV) Dialkyl Complexes Supported by Rigid Tridentate 4,5-Bis(anilido)xanthene and 2,6-Bis(anilidomethyl)pyridine Ligands. *Organometallics* **26**, 692–701 (2007).
2. Motolko, K. S. A., Emslie, D. J. H. & Jenkins, H. A. Yttrium and Aluminum Alkyl Complexes of a Rigid Bis-Anilido NON-Donor Ligand: Synthesis and Hydroamination Catalysis. *Organometallics* **36**, 1601–1608 (2017).
3. McMullen, J. S., Huo, R., Vasko, P., Edwards, A. J. & Hicks, J. Anionic Magnesium and Calcium Hydrides: Transforming CO into Unsaturated Disilyl Ethers. *Angew. Chem.* **135**, e202215218 (2023).
4. Neese, F. The ORCA program system. *WIREs Comput. Mol. Sci.* **2**, 73–78 (2012).
5. Neese, F., Wennmohs, F., Becker, U. & Riplinger, C. The ORCA quantum chemistry program package. *J. Chem. Phys.* **152**, 224108 (2020).
6. Neese, F. Software update: the ORCA program system, version 4.0. *WIREs Comput. Mol. Sci.* **8**, e1327 (2018).
7. Furness, J. W., Kaplan, A. D., Ning, J., Perdew, J. P. & Sun, J. Accurate and Numerically Efficient r2SCAN Meta-Generalized Gradient Approximation. *J. Phys. Chem. Lett.* **11**, 8208–8215 (2020).
8. Furness, J. W., Kaplan, A. D., Ning, J., Perdew, J. P. & Sun, J. Correction to "Accurate and Numerically Efficient r2SCAN Meta-Generalized Gradient Approximation". *J. Phys. Chem. Lett.* **11**, 9248–9248 (2020).
9. Grimme, S., Hansen, A., Ehlert, S. & Mewes, J.-M. r2SCAN-3c: A "Swiss army knife" composite electronic-structure method. *J. Chem. Phys.* **154**, 064103 (2021).

10. Caldeweyher, E., Bannwarth, C. & Grimme, S. Extension of the D3 dispersion coefficient model. *J. Chem. Phys.* **147**, 034112 (2017).

11. Caldeweyher, E. *et al.* A generally applicable atomic-charge dependent London dispersion correction. *J. Chem. Phys.* **150**, 154122 (2019).

12. Kruse, H. & Grimme, S. A geometrical correction for the inter- and intra-molecular basis set superposition error in Hartree-Fock and density functional theory calculations for large systems. *J. Chem. Phys.* **136**, 154101 (2012).

13. E. D. Glendening, J, K. Badenhoop, A. E. Reed, J. E. Carpenter, J. A. Bohmann, C. M. Morales, P. Karafiloglou, C. R. Landis, and F. Weinhold. *NBO 7.0*. (Theoretical Chemistry Institute, University of Wisconsin, Madison, 2018).

14. Lu, T. & Chen, F. Multiwfn: A multifunctional wavefunction analyzer. *J. Comput. Chem.* **33**, 580–592 (2012).

15. Todd A. Keith, TK Gristmill Software, Overland Park KS, USA, 2019 (aim.tkgristmill.com). *AIMAll (Version 19.10.12)*.

16. Roesky, H. W. & Keller, K. Trimethyltin fluoride: A new fluorinating reagent for the preparation of silicon fluorides. *J. Fluor. Chem.* **89**, 3–4 (1998).

17. Lorberth, J. Alkyltin tetrafluoroborates. *J. Organomet. Chem.* **17**, 151–154 (1969).

18. Panda, T. K. *et al.* Rare Earth and Alkaline Earth Metal Complexes with Me2Si-Bridged Cyclopentadienyl-Imidazolin-2-Imine Ligands and Their Use as Constrained-Geometry Hydroamination Catalysts. *Eur. J. Inorg. Chem.* **2008**, 4270–4279 (2008).

19. Ojeda-Amador, A. I., Martínez-Martínez, A. J., Kennedy, A. R. & O'Hara, C. T. Structural Studies of Cesium, Lithium/Cesium, and Sodium/Cesium Bis(trimethylsilyl)amide (HMDS) Complexes. *Inorg. Chem.* **55**, 5719–5728 (2016).

20. Bytheway, I., Gillespie, R. J., Tang, T.-H. & Bader, R. F. W. Core Distortions and Geometries of the Difluorides and Dihydrides of Ca, Sr, and Ba. *Inorg. Chem.* **34**, 2407–2414 (1995).

21. Barrett, A. G. M., Crimmin, M. R., Hill, M. S., Hitchcock, P. B. & Procopiou, P. A. Trifluoromethyl Coordination and C-F Bond Activation at Calcium. *Angew. Chem. Int. Ed.* **46**, 6339–6342 (2007).

22. Nembenna, S. *et al.* A Well-Defined Hydrocarbon-Soluble Calcium Monofluoride, [LCaF(thf)$_2$]: The Application of Soluble Calcium Derivatives for Surface Coating. *Angew. Chem. Int. Ed.* **46**, 2512–2514 (2007).

23. Wilson, A. S. S., Hill, M. S., Mahon, M. F., Dinoi, C. & Maron, L. Dehydrohalogenation of halobenzenes and C(sp3)-X (X = F, OPh) bond activation by a molecular calcium hydride. *Tetrahedron* **82**, 131931 (2021).

24. Schuhknecht, D., Spaniol, T. P., Yang, Y., Maron, L. & Okuda, J. Reactivity of a Molecular Calcium Hydride Cation ([CaH]$^+$) Supported by an NNNN Macrocycle. *Inorg. Chem.* **59**, 9406–9415 (2020).

25. N. Keyzer, E. *et al.* Synthesis of Ca(PF$_6$)$_2$, formed via nitrosonium oxidation of calcium. *Chem. Commun.* **53**, 4573–4576 (2017).

26. B. Deacon, G., C. Junk, P. & J. Moxey, G. Synthesis of fluoro(aryloxo)alkaline earth metal cages by C–F bond activation. *Dalton Trans.* **39**, 5620–5622 (2010).

27. Liu, F.-Q., Stalke, D. & Roesky, H. W. (C$_5$Me5)TiF$_2$—A Versatile Building Block for Generating Large Soluble Dimetallic Aggregates. *Angew. Chem. Int. Ed. Engl.* **34**, 1872–1874 (1995).

28. Demsar, A. *et al.* Calcium fluoride incorporated in soluble organometallics: adduct formation and solution dynamics. *J. Chem. Soc. Dalton Trans.* 4043–4048 (1998) doi:10.1039/A806209I.

29. Pevec, A., Demsar, A., Gramlich, V., Petricek, S. & Roesky, H. W. Reactions of molecular CaF_2 with [(C_5Me_5)TiF_3] and [(C_5Me_4Et)TiF_3]: symbiosis between ionic solids and organometallic compounds. *J. Chem. Soc. Dalton Trans.* 2215–2216 (1997).

30. Pavan, B., Ceresoli, D., Tecklenburg, M. M. J. & Fornari, M. First principles NMR study of fluorapatite under pressure. *Solid State Nucl. Magn. Reson.* **45–46**, 59–65 (2012).

31. Ashur, I., Allouche-Arnon, H. & Bar-Shir, A. Calcium Fluoride Nanocrystals: Tracers for In Vivo 19F Magnetic Resonance Imaging. *Angew. Chem. Int. Ed.* **57**, 7478–7482 (2018).

32. Kläring, P., Jungton, A.-K., Braun, T. & Müller, C. Synthesis, Structure and Reactivity of Iridium Hydrido Fluorido Complexes. *Eur. J. Inorg. Chem.* **2012**, 1430–1436 (2012).

33. Bornemann, D. *et al.* Deoxygenative Fluorination of Phosphine Oxides: A General Route to Fluorinated Organophosphorus(V) Compounds and Beyond. *Angew. Chem. Int. Ed.* **59**, 22790–22795 (2020).

34. Scattolin, T., Deckers, K. & Schoenebeck, F. Direct Synthesis of Acyl Fluorides from Carboxylic Acids with the Bench-Stable Solid Reagent (Me4N)SCF3. *Org. Lett.* **19**, 5740–5743 (2017).

35. Bamford, K. L., Chitnis, S. S., Qu, Z. & Stephan, D. W. Interactions of C−F Bonds with Hydridoboranes: Reduction, Borylation and Friedel–Crafts Alkylation. *Chem. – Eur. J.* **24**, 16014–16018 (2018).

36. Wang, L. & Cornella, J. A Unified Strategy for Arylsulfur(VI) Fluorides from Aryl Halides: Access to Ar-SOF3 Compounds. *Angew. Chem. Int. Ed.* **59**, 23510–23515 (2020).
